# Iodine(III) promotes cross-dehydrogenative coupling of N-hydroxyphthalimide and unactivated C(sp^3^)–H bonds

**DOI:** 10.1038/s42004-021-00480-8

**Published:** 2021-03-31

**Authors:** Fufang Wu, Xuanzhen Han, Xuejian Li, Xiaobao Shen, Chang Wang, Zhimei Tian, Bin Cheng, Jingbin Zhang, Liangquan Sheng, Hongbin Zhai

**Affiliations:** 1 Engineering Research Centre of Biomass Conversion and Pollution Prevention Control of Anhui Provincial Department of Education, Fuyang Normal University, Fuyang, China; 2 State Key Laboratory of Chemical Oncogenomics, Shenzhen Engineering Laboratory of Nano Drug Slow-Release, Peking University Shenzhen Graduate School, Shenzhen, China; 3 Institute of Marine Biomedicine, Shenzhen Polytechnic, Shenzhen, China; 4 Youcare Pharmaceutical Group CO, LTD, Fuyang, China

**Keywords:** Synthetic chemistry methodology, Synthetic chemistry methodology

## Abstract

Cross-dehydrogenative coupling reactions provide a method to construct new chemical bonds by direct C–H activation without any pre-functionalization. Compared to functionalization of a C–H bond α- to ether oxygen, α- to carbonyl, or at a benzylic position, functionalization of unactivated hydrocarbons is difficult and often requires high temperatures, a transition-metal catalyst, or a superstoichiometric quantity of volatile, toxic, and explosive *tert-*butylhydroperoxide. Here, a cross-dehydrogenative C–O coupling reaction of N-hydroxyphthalimide with unactivated alkanes, nitriles, ethers, and thioethers has been realized by using iodobenzene diacetate as the radical initiator. The current protocol enables efficient functionalization of unactivated hydrocarbons and nitriles through inert C(sp^3^)–H bond activation under mild reaction conditions. O-substituted NHPI derivatives are generated in good yields under metal-free conditions.

## Abstract (web summary)

Robust and selective C–H functionalisation of alkanes remains a challenge. Here, iodine(III) mediates the radical coupling of N-hydroxyphthalimide and unactivated C(sp^3^)–H bonds in a range of cyclic and acyclic alkanes.

## Introduction

Cross-dehydrogenation coupling (CDC), a type of chemical bond construction by direct dehydrogenation, has emerged as an important approach in chemical synthesis because of its excellent efficiency, atomic economy, and wide substrate scope^1–4^. Catalysts based on transition metals such as copper, iron, ruthenium, and manganese are typically used in CDC reactions^5–7^. However, issues associated with these metallic catalysts, such as high cost, harsh reaction conditions, toxicity, and metal residues, have restricted their application in organic synthesis^8,9^. Therefore, the development of efficient, simple, environmentally friendly, and metal-free CDC reactions has become a major trend^10^.

**Fig. 1:**
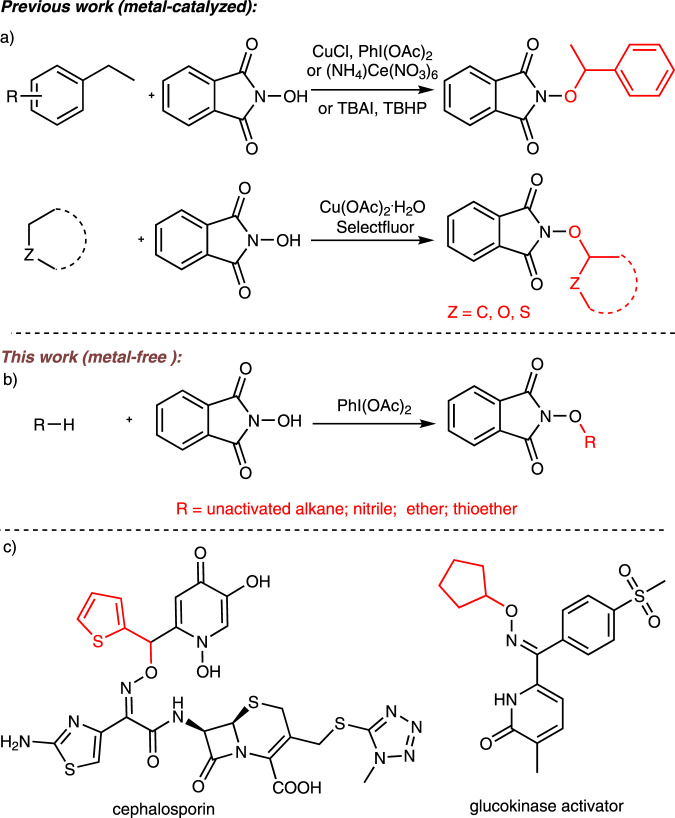
Cross-dehydrogenative C–O coupling using NHPI. **a** Approaches for the cross-coupling reactions under transition metal catalyst or *tert*-butylhydroperoxide. **b** Cross-dehydrogenative C–O coupling reaction of N-hydroxyphthalimide with unactivated C(sp^3^)–H by using iodobenzene diacetate. **c** Structures of cephalosporin and glucokinase activator.

In 1985, the Masui group first applied N-hydroxyphthalimide (NHPI) as a metal-free oxidant to the oxidation of olefins via a free radical reaction^11^. The ‘non-persistent’ nitrogen-oxygen radical, i.e. phthalimide nitrogen-oxygen (PINO) radical^12^, is often used to activate inert hydrocarbon bonds due to its high activity^13^. Moreover, oxygen-substituted NHPI derivatives could be converted into alkoxyamines via hydrazinolysis^14^. Alkoxyamines can be further used to synthesize new cephalosporins, oxiconazoles, glucokinase activators, and other organic compounds with antibacterial activity^15–17^. Disadvantages of conventional synthetic methods for accessing these compounds include poor atom economy, tedious synthetic procedures, and unsatisfactory selectivity. Recent researches on the CDC reactions involving NHPI have focused mainly on activated C(sp^3^)–H bonds. Reaction sites are typically at benzylic positions or at the α position of carbonyl moieties or ethers^18–39^. The CDC reaction between NHPI and alkanes with inert C(sp^3^)–H bonds has rarely been studied, and only few examples have been reported so far (Fig. 1)^40–43^. For example, copper nitrate trihydrate-catalyzed functionalization of C(sp^3^)–H bonds adjacent to the oxygen atoms of ethers was realized with oxygen as the co-oxidant in 2017^37^. Functionalization either α- to the oxygen atom of ethers or at the benzylic position of benzyl derivatives was accomplished with tetrabutylammonium iodide and *tert-*butylhydroperoxide under sonication^31^. It is noteworthy that the substrates in the previous reports did not involve acyclic alkanes or nitriles, and transition metal catalysts or superstoichiometric quantities of (volatile, toxic and explosive) *tert*-butylhydroperoxide were often required.

Hypervalent iodine can be used as an initiator to trigger various chemical reactions, functioning like a transition metal catalyst, and this reagent has been widely applied to drug synthesis and total synthesis of natural products^44–48^. Considering the potential value of R–ONH_2_ compounds, we herein report an approach to constructing C–O bonds in unactivated alkanes, cyanides, ethers, and thioethers via a CDC reaction with NHPI in the presence of iodobenzene diacetate as a radical initiator (Fig. 1). The current method features a number of advantages, such as mild metal-free reaction conditions, high synthetic efficiency, environmentally friendliness, and applicability to a wide range of substrates.

## Results and discussion

### Reaction optimization

**Fig. 2:**
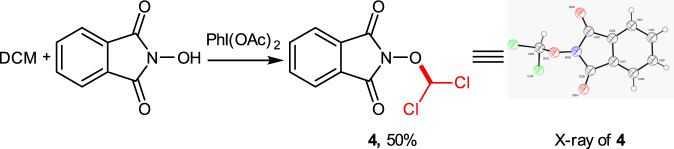
Reaction of DCM with NHPI. DCM as both the solvent and the substrate.

The project was commenced with the reaction of cyclohexane with NHPI at room temperature, in which the effect of oxidants [*tert*-butyl hydroperoxide (TBHP), PhI(OCOCF_3_)_2_, H_2_O_2_, and PhI(OAc)_2_] was investigated (Table 1, entries 1–5). The NHPI derivative **3** was formed in 40% yield only when PhI(OAc)_2_ was applied as the oxidant (entry 5). The solvents [chloroform, water, N,N-dimethylacetamide (DMA), 1,2-dichloroethane (DCE), acetonitrile (MeCN), ethyl acetate (EA), chlorobenzene (PhCl), dichloromethane (DCM), and benzene (PhH)] were next screened (entries 6–14). When PhI(OAc)_2_ was used as the oxidant, DCM (entry 13) and benzene (entry 14) gave the best results. The reaction with PhI(OCOCF_3_)_2_ as the oxidant in DCM and PhH was then investigated, and the desired product **3** was obtained in 42 and 49% yield, respectively (entries 15, 16). The influence of the temperature (0 ^o^C and 60 ^o^C) on the reaction was examined with DCM as the solvent and PhI(OAc)_2_ as the oxidant, and no better result was obtained (entries 17, 18). Notably, when DCM was used as the solvent, compound **4** was formed (15%) along with **3**; the formation of **4** was obviously due to the reaction of the solvent molecule itself with NHPI (see Fig. 2 for the structure). The bond dissociation enthalpies (BDEs) for cyclohexane (cyclohexane-H) and DCM (DCM-H) were calculated by Gaussian 16 at B3LYP/6-31 G basis set level and found to be 402.3 and 416.5 kJ mol^−1^, respectively, which indicates that cyclohexane should be slightly more prone to undergo this kind of reaction.

**Table 1:** Optimisation of the reaction conditions^a^.


Entry	1:2:Oxidant	Oxidant	Solvent	T (°C)	Yield^b^
1^c^	20:1:2	TBHP	–	rt	N.D.
2	2:1:2	TBHP	H_2_O	rt	N.D.
3^c^	20:1:2	PhI(OCOCF_3_)_2_	–	rt	trace
4^c^	20:1:2	H_2_O_2_	–	rt	N.D.
5^c^	20:1:2	PhI(OAc)_2_	–	rt	40
6	2:1:2	PhI(OAc)_2_	CHCl_3_	rt	39
7	2:1:2	PhI(OAc)_2_	H_2_O	rt	16
8	2:1:2	PhI(OAc)_2_	DMA	rt	N.D.
9	2:1:2	PhI(OAc)_2_	DCE	rt	43
10	2:1:2	PhI(OAc)_2_	MeCN	rt	46
11	2:1:2	PhI(OAc)_2_	EA	rt	32
12	2:1:2	PhI(OAc)_2_	C_6_H_5_Cl	rt	42
13	2:1:2	PhI(OAc)_2_	DCM	rt	61
14	2:1:2	PhI(OAc)_2_	PhH	rt	66
15	2:1:2	PhI(OCOCF_3_)_2_	DCM	rt	42
16	2:1:2	PhI(OCOCF_3_)_2_	PhH	rt	49
17	2:1:2	PhI(OAc)_2_	DCM	0	25
18^d^	2:1:2	PhI(OAc)_2_	DCM	60	54

^a^General procedure: the reaction was carried out with NHPI (1 mmol), cyclohexane (2 mmol), oxidant (2 mmol), and solvent (2 mL) at room temperature for 2 h.

^b^Isolated yield.

^c^Cyclohexane (20 mmol) was used as the substrate and solvent.

^d^The reaction was conducted in reflux.

When the reaction was performed with DCM as both the solvent and the substrate, compound **4** was obtained in 50% yield (Fig. 2). Although no additional coupling products were produced when benzene was adopted as the solvent, considering the toxicity of benzene, DCM was chosen as the solvent for most of the subsequent experiments.

### Substrate scope

**Fig. 3:**
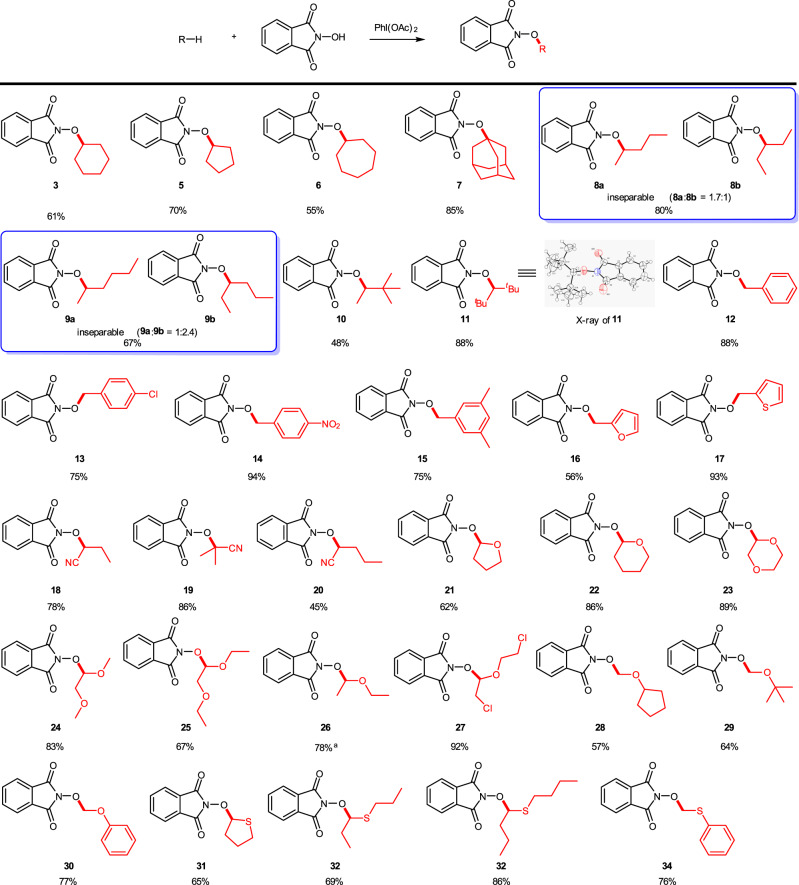
CDC coupling of various C–H reagents with NHPI. General procedure: the reactions were carried out with NHPI (1 mmol), alkane (2 mmol), PhI(OAc)_2_ (2 mmol) in DCM (2 mL) at room temperature for 2 h. Isolated yield. ^a^The substrate (20 mmol) was used as the solvent.

The substrate scope was then explored under the optimal reaction conditions obtained (see Table 1, entry 15), and the experimental results are summarized in Fig. 3. First, the reactions of the cycloalkanes with NHPI were investigated. The C–O CDC reactions of cyclopentane, cycloheptane, and amantadine with NHPI proceeded smoothly and afforded compounds (**5**–**7**) in the yields of 55–85%. It should be mentioned that no reports have been disclosed concerning this kind of C–H activation for acyclic alkanes so far. The reactions of acyclic alkanes (e.g., *n*-pentane and *n*-hexane) with NHPI were then tested and inseparable oxidation products **8a**/**8b** (1.7/1) and **9a**/**9b** (1/2.4) were obtained, respectively, while single products were delivered from the reaction of 2,2-dimethylbutane and 2,2,4,4-tetramethylpentane in 48% (**10**) and 88% (**11**) yields, respectively. In these cases, no activation of the primary C(sp^3^)–H bond occurred. Further studies revealed that toluene, trimethylbenzene, *p*-nitrotoluene, *p*-chlorotoluene, 2-methylfuran, and 2-methylthiophene were suitable substrates as well, and the corresponding oxidation products (**12**–**17**) were afforded in 56–94% yields. The benzyl C(sp^3^)–H bond was involved in the CDC reaction for these substrates, and the chloro substituent on the phenyl ring does not affect the desired transformation essentially.

Cyano compounds play an important role in drug synthesis^49^, and several research groups have realised direct oxidation of α–C(sp^3^)–H in alkyl nitrile with metal catalysts^50,51^. Hence, alkyl nitrile substrates were next studied under the current transition metal-free conditions. For butyronitrile, isobutyronitrile, and pentonitrile, the corresponding α–C(sp^3^)–H bond oxidation products (**18**–**20**) were generated in the yields of 45–86%. The CDC reaction of various ethers and sulfides was also investigated. Cyclic ethers (**21**–**23**), acyclic ethers (**24**–**30**), thioethers (**31**–**34**), and even haloether (**27**) were converted into the corresponding oxidation products smoothly.

### Synthetic applications

**Fig. 4:**
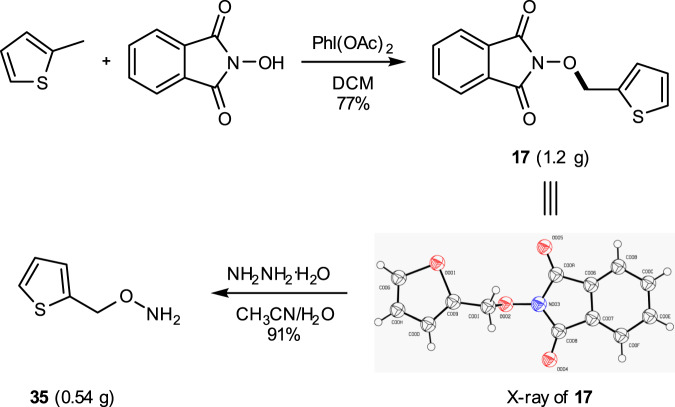
A gram-scale experiment. Gram-scale with 2-methylthiophene and hydrazinolysis.

A gram-scale reaction was performed to demonstrate the application of the current synthetic method. O-(Thiophen-2-ylmethyl) hydroxylamine is a key intermediate for the synthesis of new cephalosporins. The CDC reaction was scaled up to gram-scale with 2-methylthiophene as the substrate and compound **17** was obtained in 77% yield (Fig. 4). Alkoxyamine **35** was then obtained via hydrazinolysis of **17**.

**Fig. 5:**
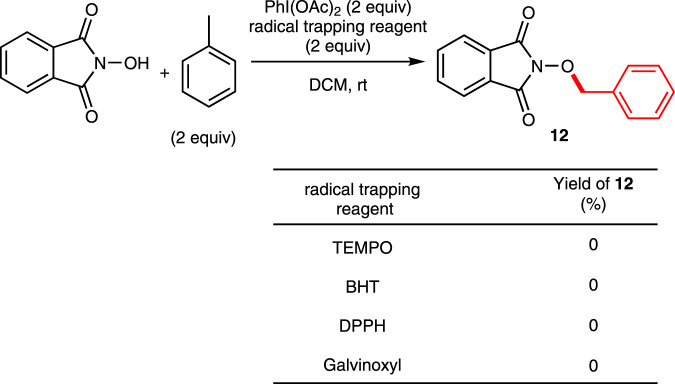
Effect of radical trapping reagents to the reaction mixture. PhI(OAc)_2_ (0.54 mmol), radical trapping reagent (0.54 mmol) and NHPI (0.27 mmol) were added to a solution of toluene (0.54 mmol) in DCM (2 mL).

Relevant mechanistic studies were next performed (Fig. 5). The reaction of toluene (as the substrate) was found to be totally inhibited upon addition of 2,2,6,6-tetramethylpiperidinooxy (TEMPO), 2,6-di-tert-butyl-4-methylphenol (BHT), 2,2-diphenyl-1-picrylhydrazyl (DPPH), or Galvinoxyl. These results indicated that the current transformation might proceed via a radical reaction pathway.

**Fig. 6:**
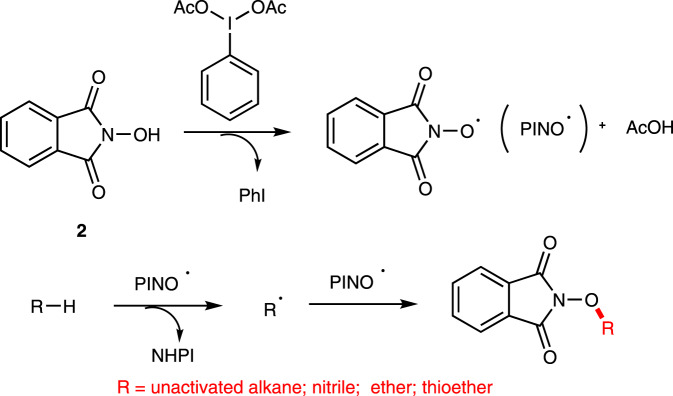
Proposed reaction mechanism. Oxidation of NHPI with PhI(OAc)_2_ affords PINO radical.

A plausible mechanism is proposed for the current reaction (Fig. 6). Oxidation of NHPI (**2**) with PhI(OAc)_2_ affords PINO radical^35^, which reacts with the substrate to form an alkyl radical via hydrogen abstraction. Finally, the alkyl radical couples with PINO radical to deliver the final product.

In conclusion, selective C–H functionalisation of alkanes remains a great challenge. PhI(OAc)_2_ has been applied to mediating radical coupling of N-hydroxyphthalimide and unactivated C(sp^3^)–H bonds in a range of cyclic and acyclic alkanes. The present reaction was conducted at room temperature which was compatible with various solvents. A variety of cycloalkanes, acyclic alkanes, cyanides, ethers, and thioethers reacted smoothly with NHPI avoiding volatile, toxic and explosive reagents, providing a direct and simple access to O-substituted NHPI derivatives.

## Methods

### General Information

All experiments were conducted under air, using commercially purchased analytical reagents and solvents which do not require further purification. ^1^HNMR and ^13^CNMR spectra were recorded on a Bruker spectrometer (at 400 and 100 MHz, respectively). TMS was used as reference for chemical shifts. High-resolution mass spectrometry (HRMS) was recorded on an Agilent Technologies LC-TOF instrument. X-ray crystallography of compounds **4**, **11**, and **17** were performed on a Bruker Smart Apex CCD area detector diffractometer using graphite-monochromated Mo Kα radiation. The regards to the suitability and safety warnings of PhH: avoid contact with the skin, the eyes; wear suitable protective clothing, gloves, eye/ face protection; avoid release to the environment.

### General procedure for the synthesis of R-ONH_2_ compounds

PhI(OAc)_2_ (2 mmol) and NHPI (1 mmol) were added to a solution of a substrate (2 mmol) in DCM (2 mL), and the reaction mixture was stirred at room temperature for 2 h and filtered. The filtrate was concentrated under reduced pressure to give a crude product, which was purified by flash silica gel column chromatography (petroleum ether/EtOAc, 50:1 to 20:1) to give the product.

### Procedure for compound 35

Water (36 mL) and N-hydrazine hydrate (1.8 g, 54 mmol) were added to a solution of 2-(thiophen-2-ylmethoxy)isoindoline-1,3-dione (1.2 g, 4.63 mmol) in acetonitrile (50 mL). The mixture was stirred at room temperature for 4 h. The crude product was purified by flash silica gel column chromatography (petroleum ether/EtOAc, 5:1) to give the product (0.54 g, 91%) as a brown solid.

### Reaction of Toluene with NHPI in the presence of radical trapping reagent

PhI(OAc)_2_ (0.54 mmol), radical trapping reagent (0.54 mmol), and NHPI (0.27 mmol) were added to a solution of toluene (0.54 mmol) in DCM (2 mL), and the reaction mixture was stirred at room temperature for 2 h and detected by TLC.

### Methods of the calculation of BDEs

The bond dissociation enthalpies (BDEs) were calculated by Gaussian 16 at B3LYP/6-31 G basis set level.

The BDEs for cyclohexane (cyclohexane-H) was calculated as:

[−234.992855 Hatree (Cyclohexane radical)] + [−0.497912 Hatree (H radical)] − [−235.644013 Hatree (Cyclohexane)] = 0.153246 Hatree = 402.3 kJ mol^−1^.

The BDEs for cyclohexane (DCM-H) was calculated as:

[−958.957469 Hatree (DCM radical)] + [−0.497912 Hatree (H radical)] − [−959.614026 Hatree (DCM)] = 0.158645 Hatree = 416.5 kJ mol^−1^.

## Supplementary information

Supplementary Information

Description of Additional Supplementary Files

Supplementary Data 1

Supplementary Data 2

Supplementary Data 3

Supplementary Data 4

Supplementary Data 5

## Data Availability

The authors declare that the data supporting the findings of this study are available within the paper and its Supplementary Information file, and from the corresponding authors upon reasonable request. The characterization data are available in Supplementary Note 1 and NMR spectra are available in Supplementary Figs. 1~66. The supplementary crystallographic data (Supplementary Data 1) reported in this study have been deposited at the Cambridge Crystallographic Data Centre (CCDC), under deposition number 2021450~2021452. These data can be obtained free of charge from The Cambridge Crystallographic Data Centre via https://www.ccdc.cam.ac.uk/. The calculated results are available in Supplementary Table 4. and XYZ co-ordinates for all optimized structures see Supplementary Data 2. The crystallographic informations of compounds **4**, **11** and **17** are available in Supplementary Data 3–5.
