# Supplementary material for: Iodine(III) promotes cross-dehydrogenative coupling of N-hydroxyphthalimide and unactivated C(sp3)–H bonds
Source: Commun Chem. 2021 Mar 31;4:46. doi: 10.1038/s42004-021-00480-8 (PMC9814821; doi:10.1038/s42004-021-00480-8)
Supplement: Supplementary file 1 — Supplementary Information [file 42004_2021_480_MOESM1_ESM.pdf]

Supporting Information

Iodine(III) promotes cross-dehydrogenative coupling of N-hydroxyphthalimide and unactivated C(sp^3)-H bonds

Fufang Wu,[1*] Xuanzhen Han,[1,2] Xuejian Li,[1] Xiaobao Shen,[1] Chang Wang,[1] Zhimei Tian,[1] Bin Cheng,[3] Jingbin Zhang,[4] Liangquan Sheng[1*] and Hongbin Zhai[2*]

[1]Engineering Research Centre of Biomass Conversion and Pollution Prevention Control of Anhui Provincial Department of Education, Fuyang Normal University, Fuyang 236037, China. [2]State Key Laboratory of Chemical Oncogenomics, Shenzhen Engineering Laboratory of Nano Drug Slow-Release, Peking University Shenzhen Graduate School, Shenzhen 518055, China. [3]Institute of Marine Biomedicine, Shenzhen Polytechnic, Shenzhen 518055, China. [4]Youcare Pharmaceutical Group CO, LTD, Fuyang 236033, China. *Email: fufang_wu@foxmail.com; E-mail: shenglq@fync.edu.cn; E-mail: zhaihb@pku.edu.cn.*

NMR Spectra

3 (^{1}H, $CDCl_3$)

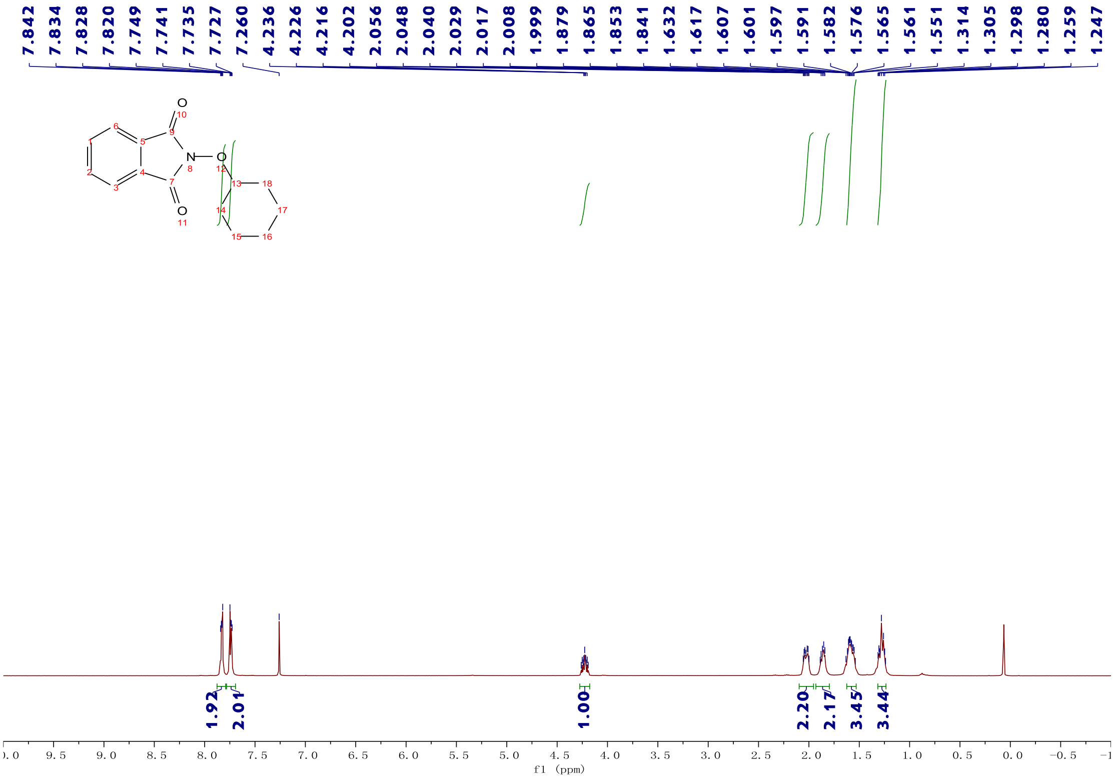

Supplementary Figure 1. ^{1}H NMR Spectra of **3**

3 (^{13}C, $CDCl_3$)

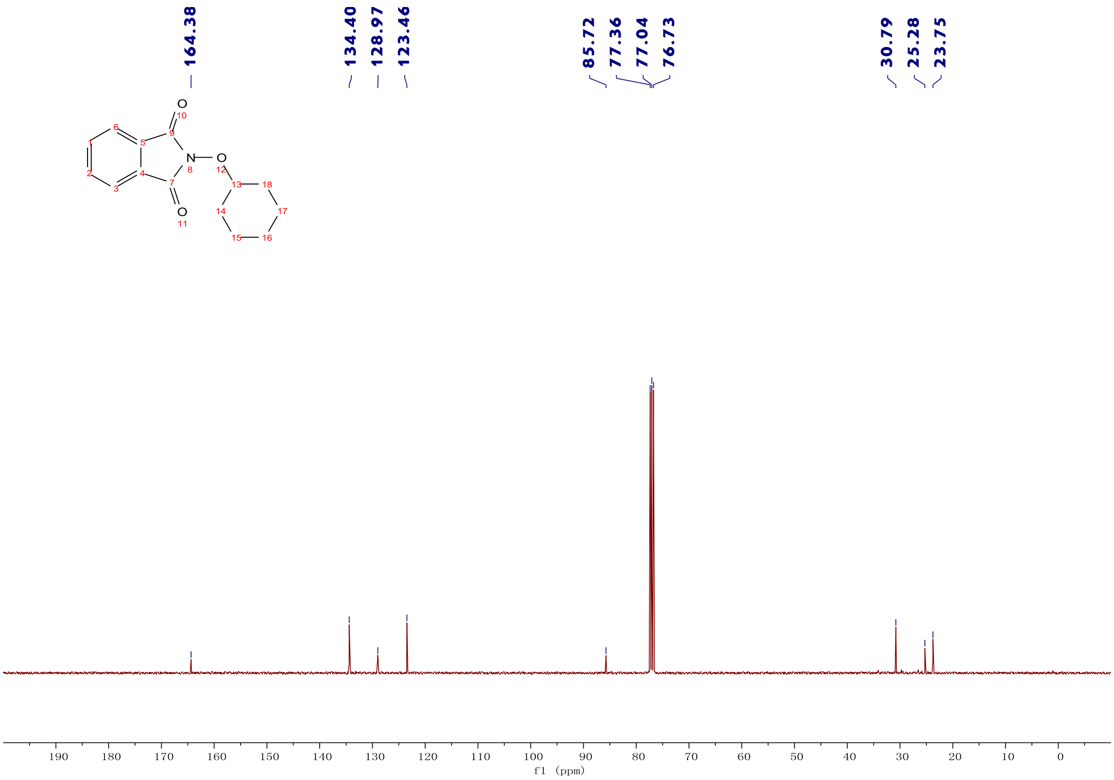

Supplementary Figure 2. ^{13}C NMR Spectra of **3**

4 (^{1}H, $CDCl_3$)

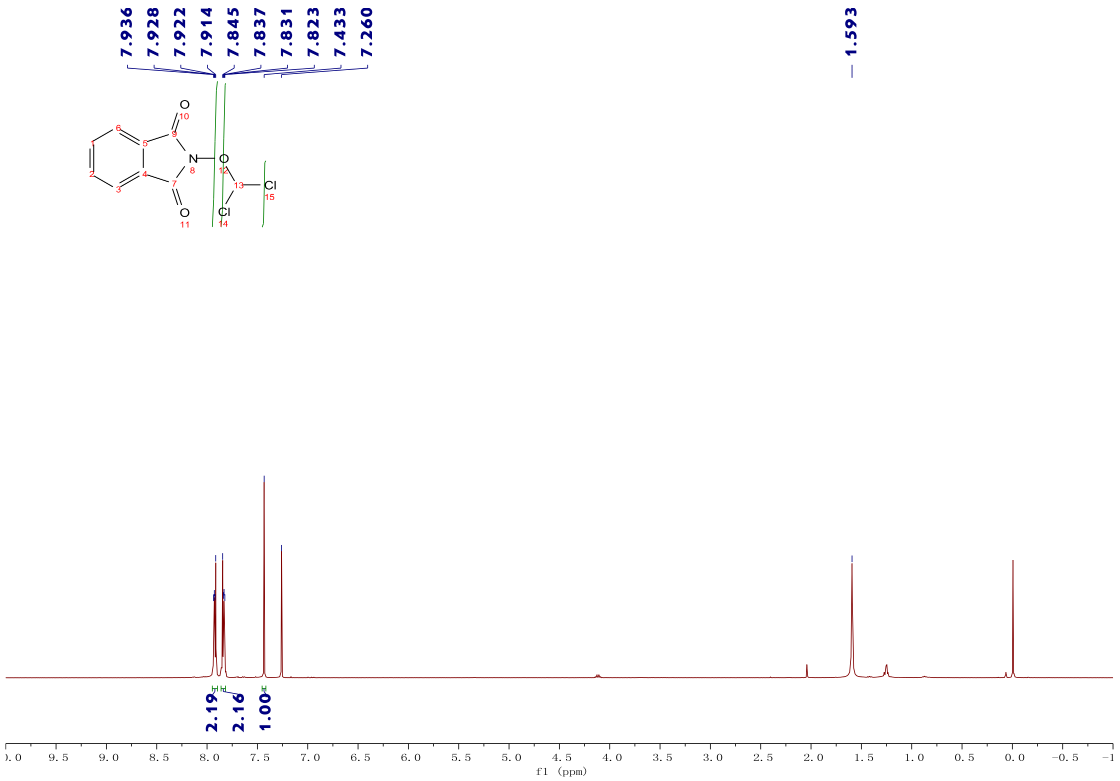

Supplementary Figure 3. ^{1}H NMR Spectra of **4**

4 (^{13}C, $CDCl_3$)

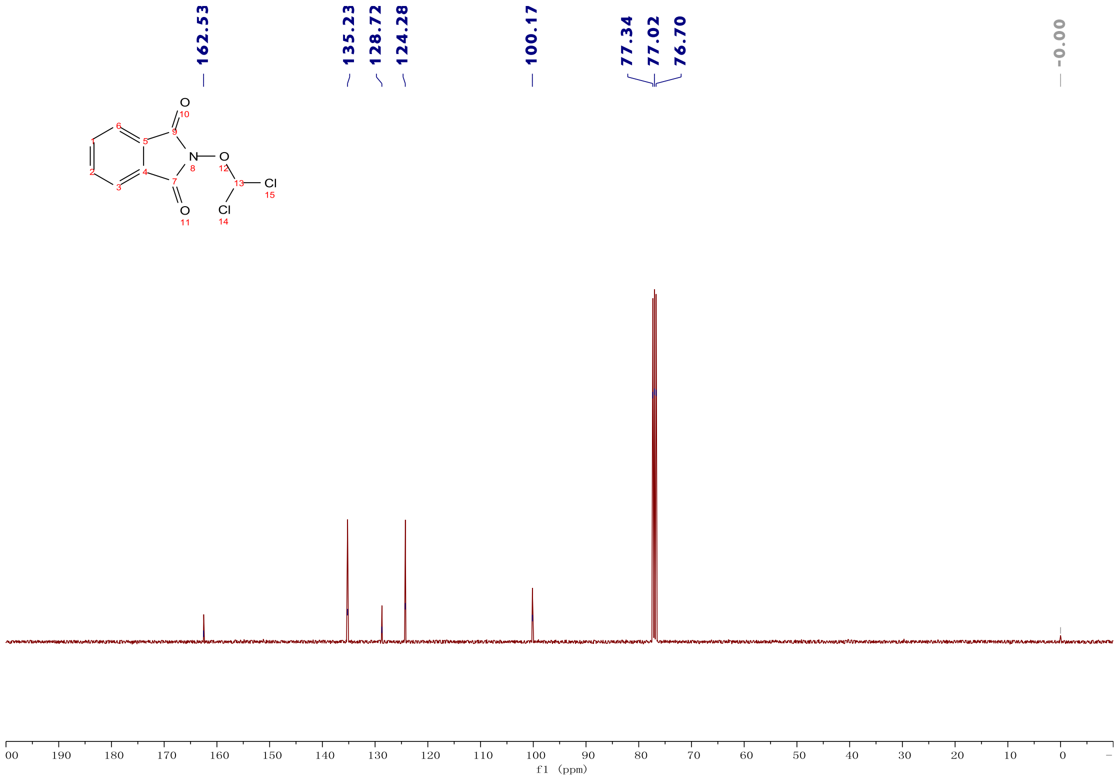

Supplementary Figure 4. ^{13}C NMR Spectra of **4**

5 (^{1}H, $CDCl_3$)

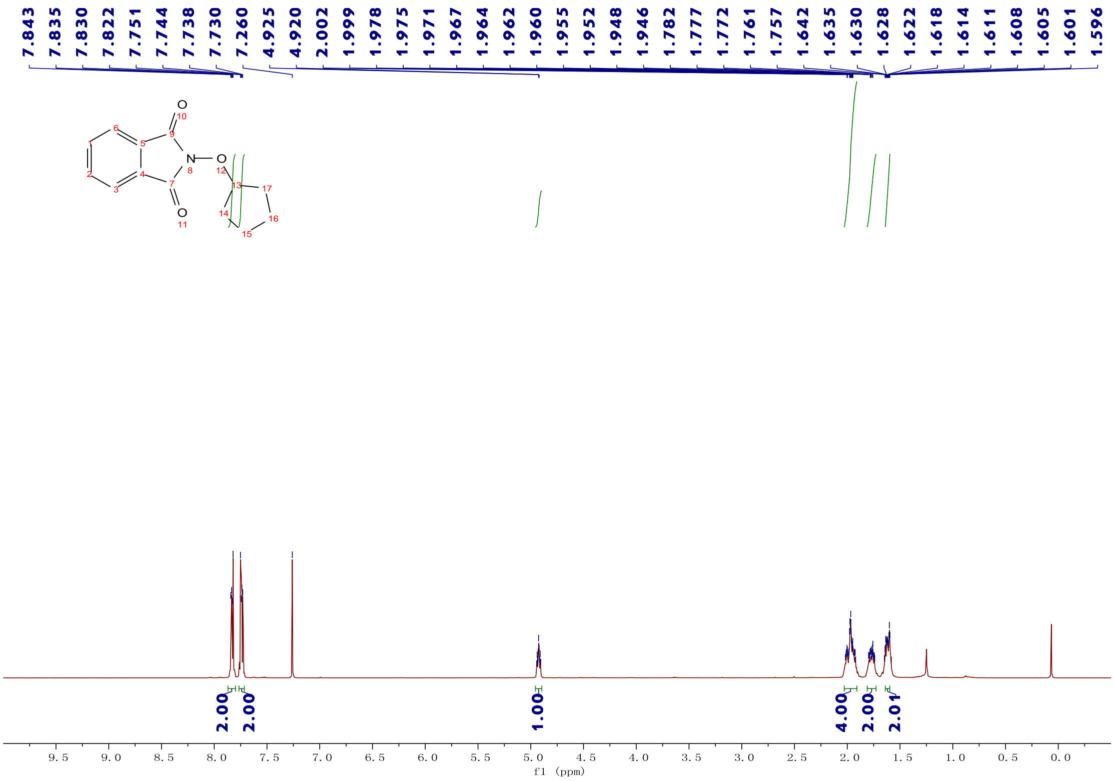

Supplementary Figure 5. ^{1}H NMR Spectra of **5**

5 (^{13}C, $CDCl_3$)

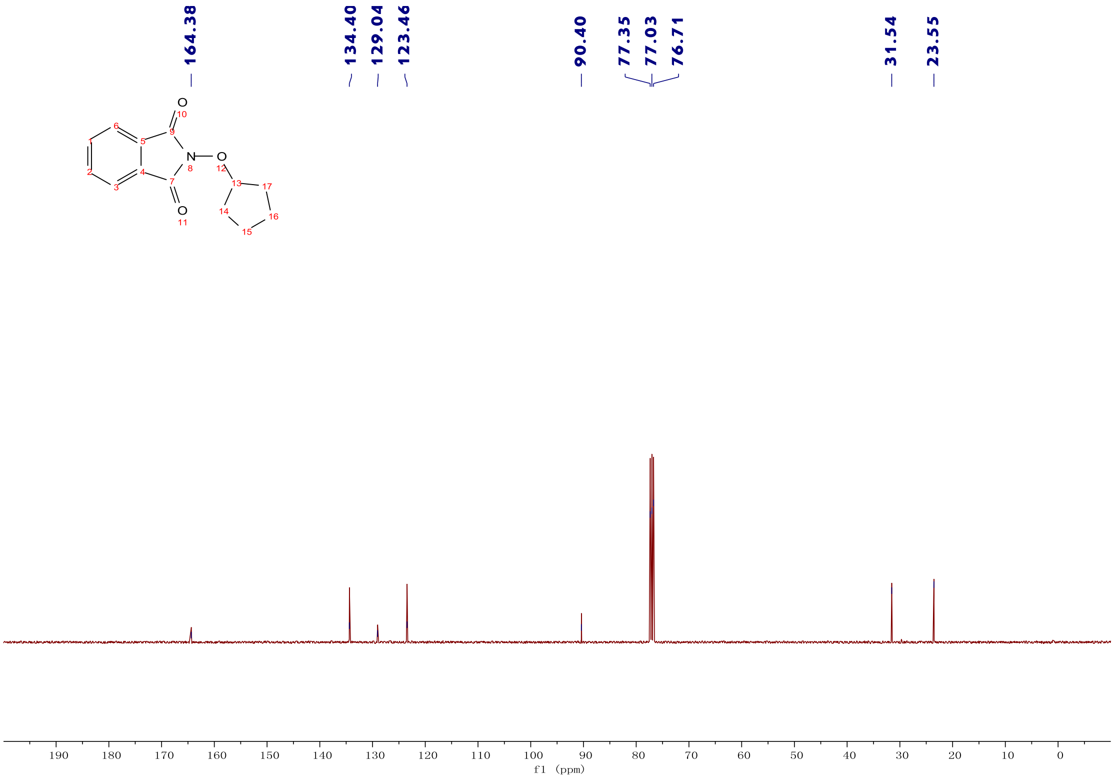

Supplementary Figure 6. ^{13}C NMR Spectra of **5**

6 (^{1}H, $CDCl_3$)

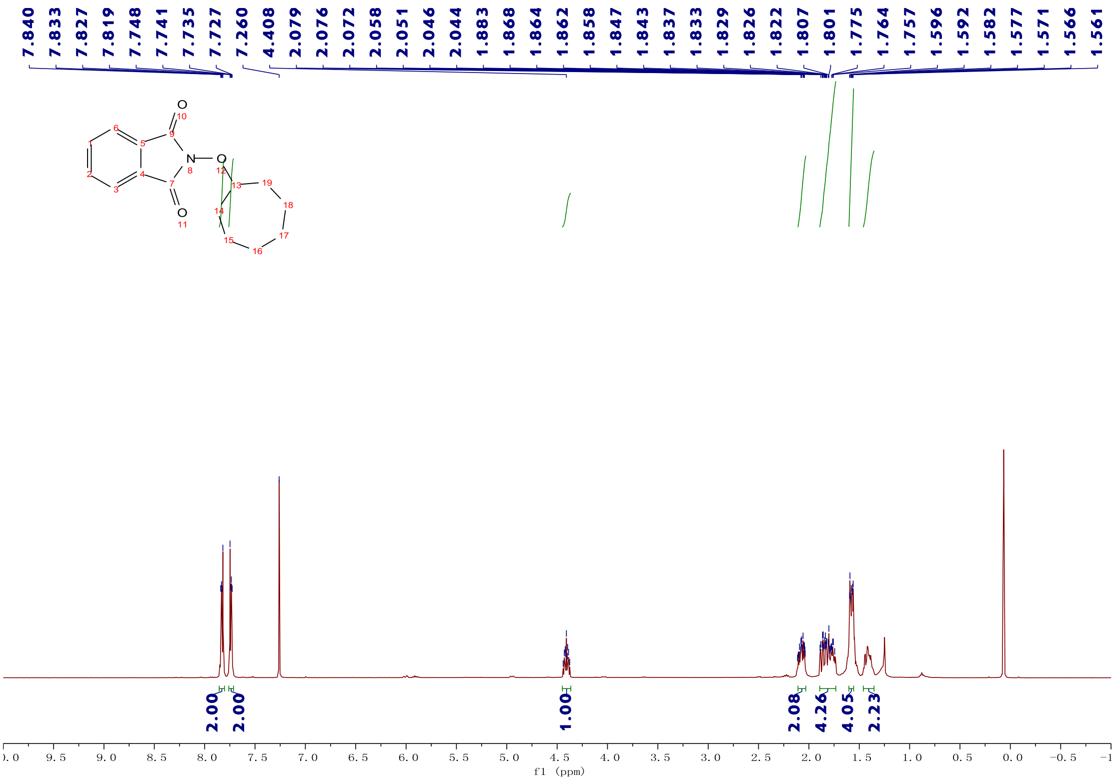

Supplementary Figure 7. ^{1}H NMR Spectra of **6**

6 (^{13}C, $CDCl_3$)

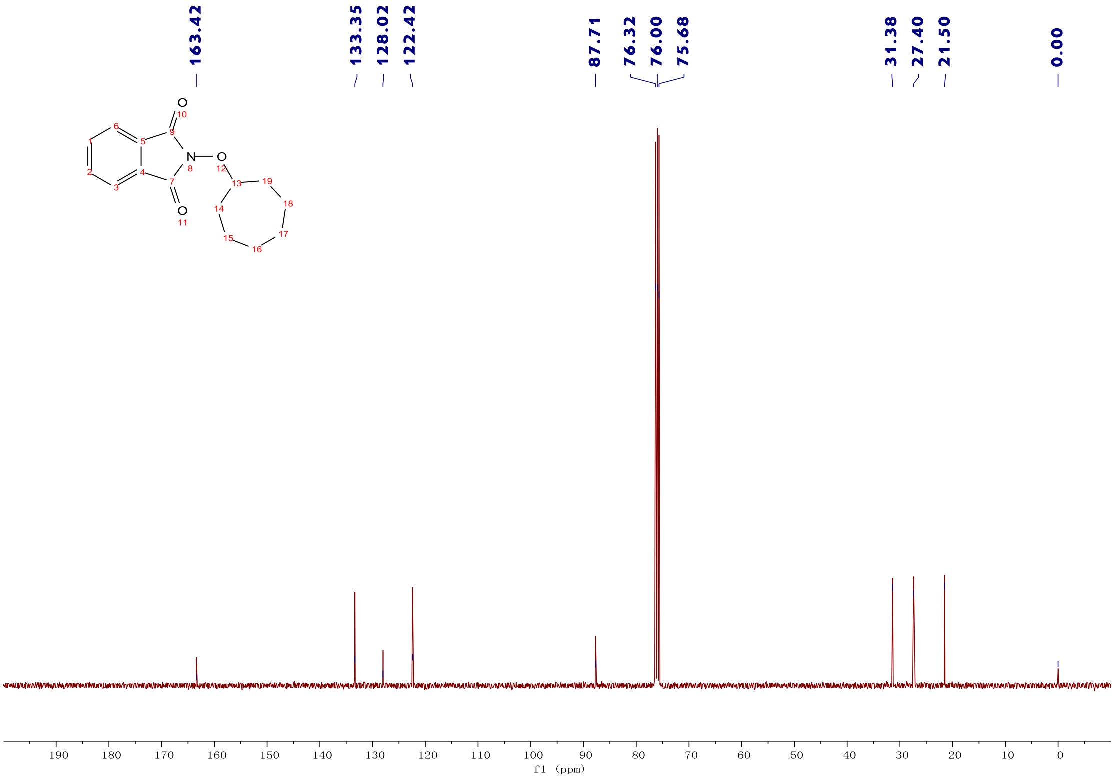

Supplementary Figure 8. ^{13}C NMR Spectra of **6**

7 (^{1}H, $CDCl_3$)

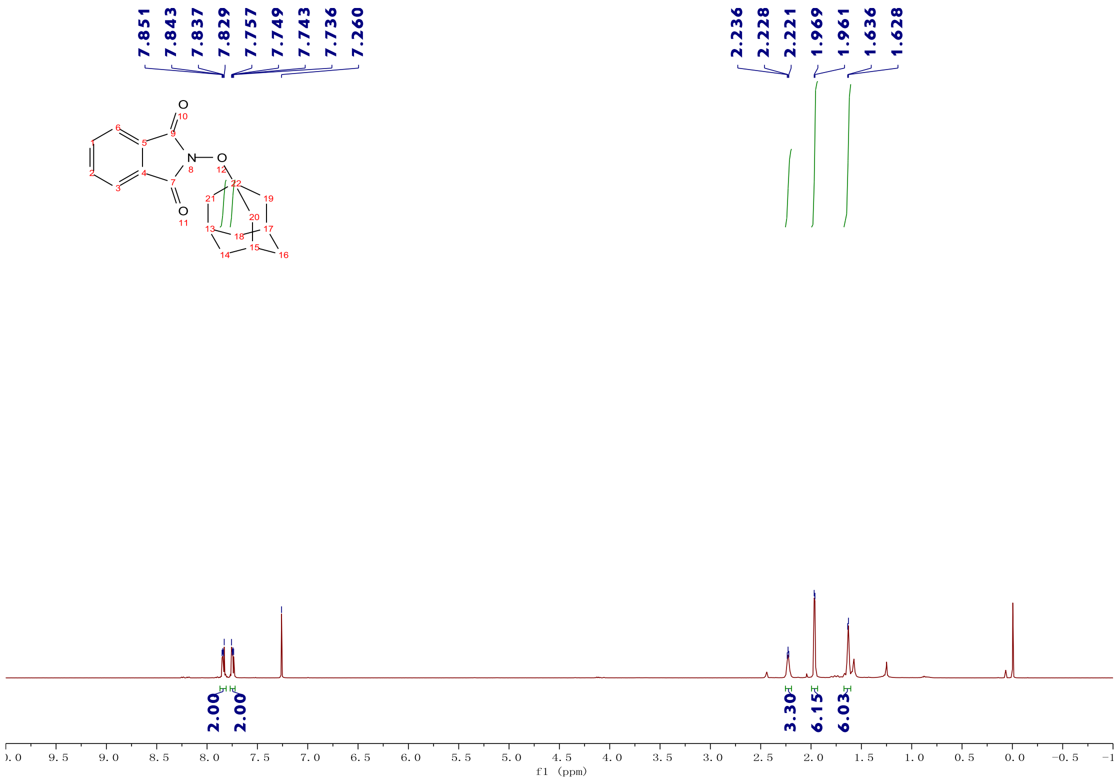

Supplementary Figure 9. ^{1}H NMR Spectra of **7**

7 (^{13}C, $CDCl_3$)

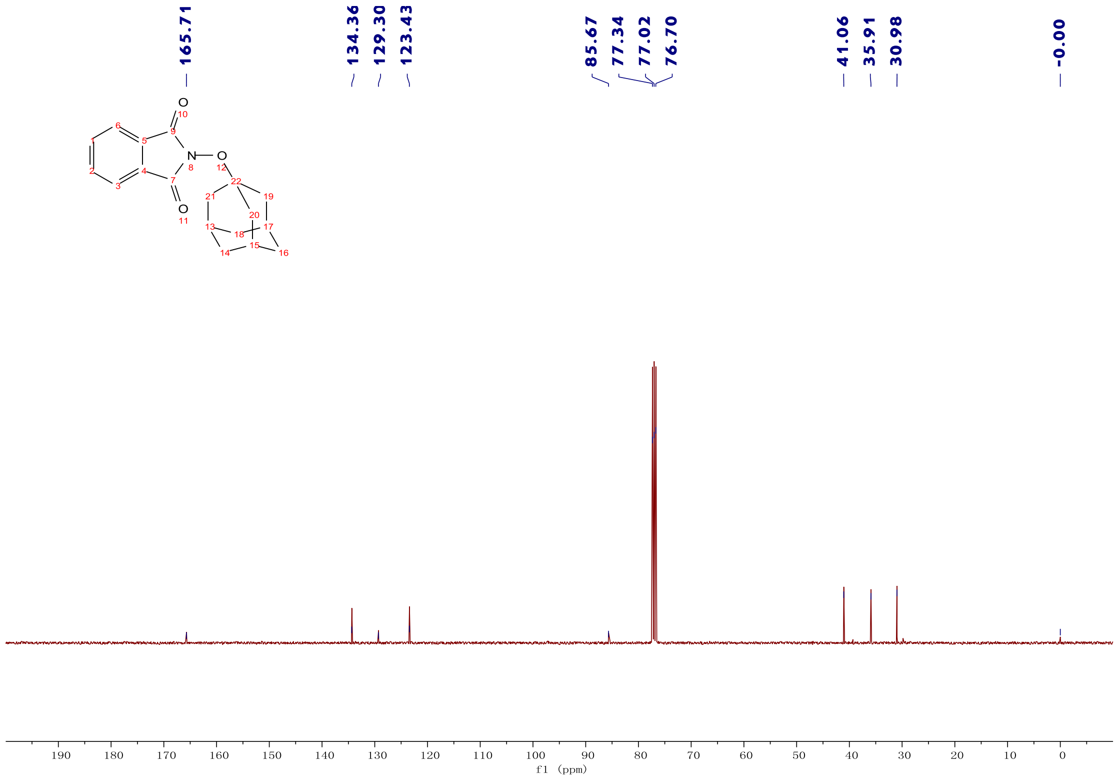

Supplementary Figure 10. ^{13}C NMR Spectra of **7**

8 (^{1}H, $CDCl_3$)

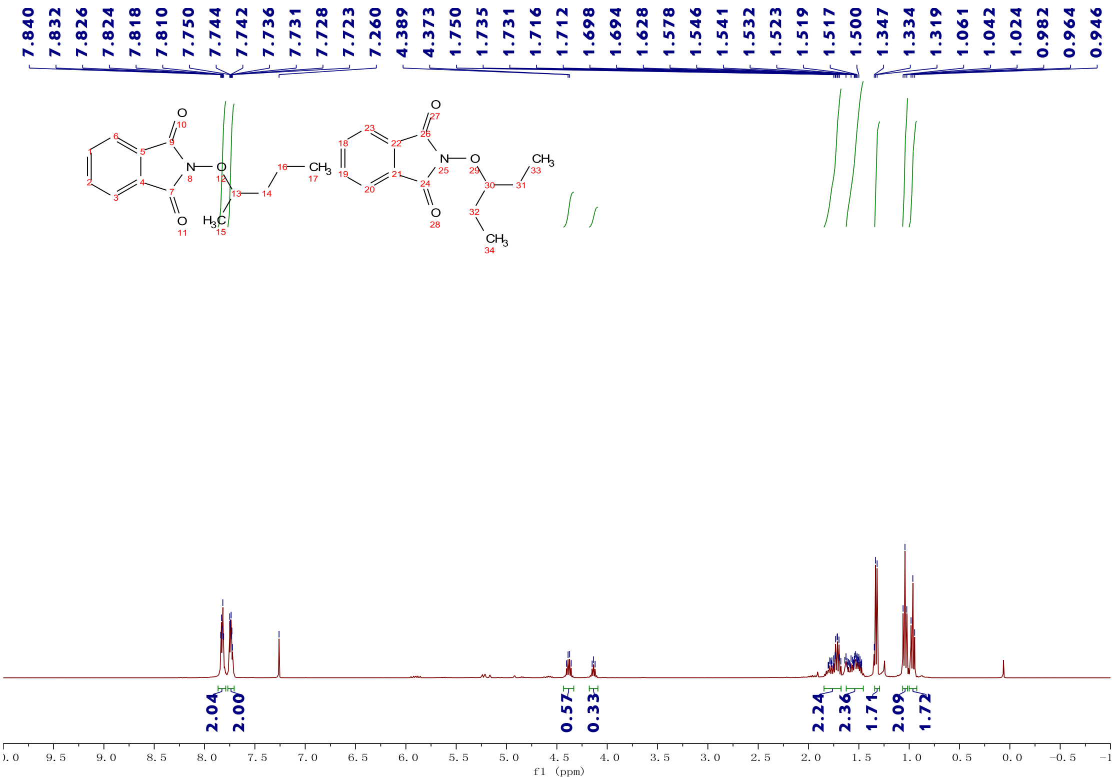

Supplementary Figure 11. ^{1}H NMR Spectra of **8**

8 (^{13}C, $CDCl_3$)

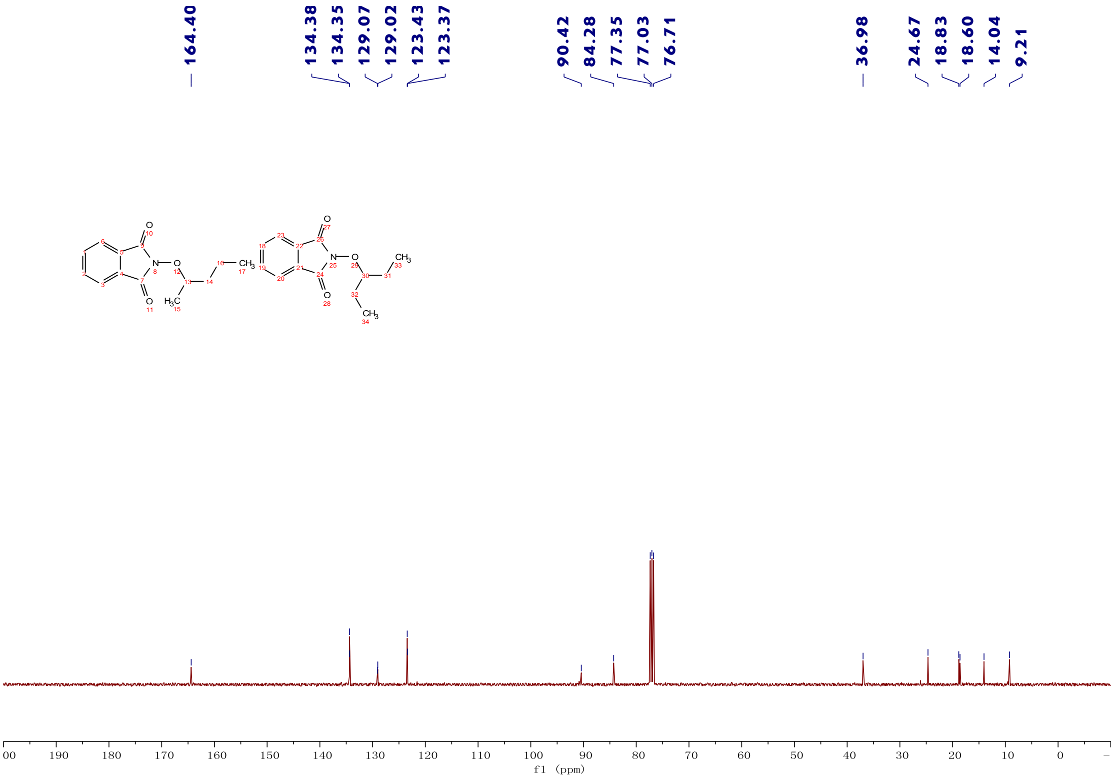

Supplementary Figure 12. ^{13}C NMR Spectra of **8**

9 (^{1}H, $CDCl_3$)

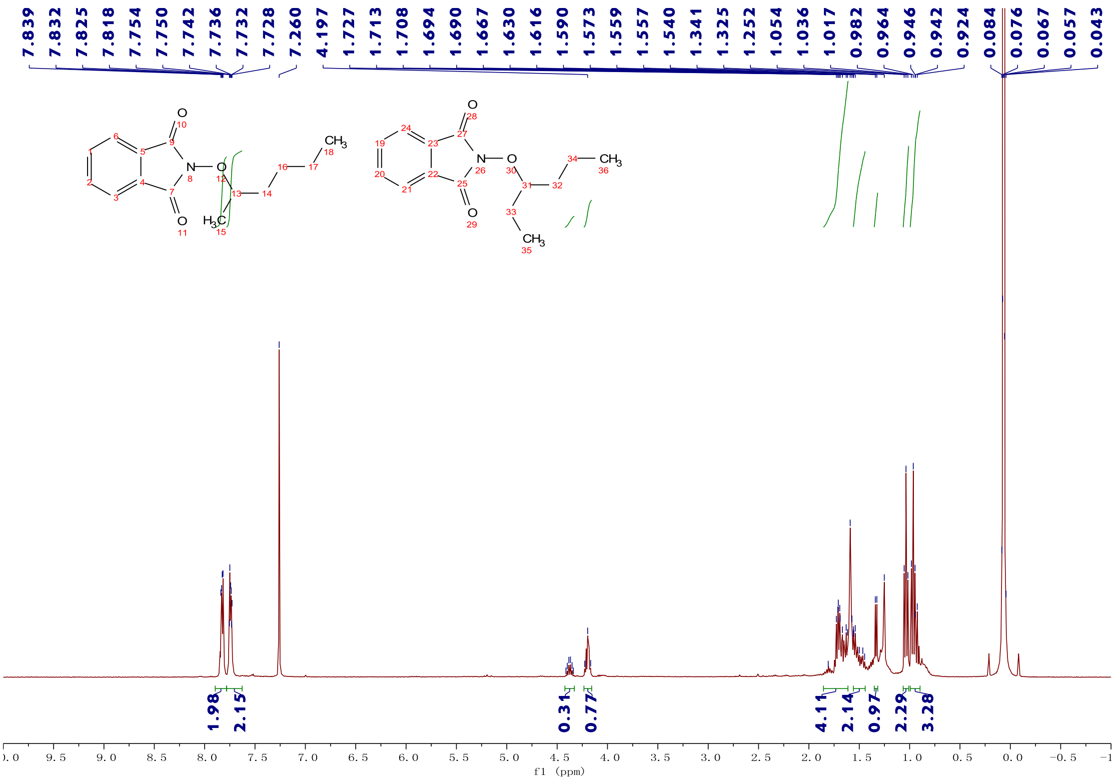

Supplementary Figure 13. ^{1}H NMR Spectra of **9**

9 (^{13}C, $CDCl_3$)

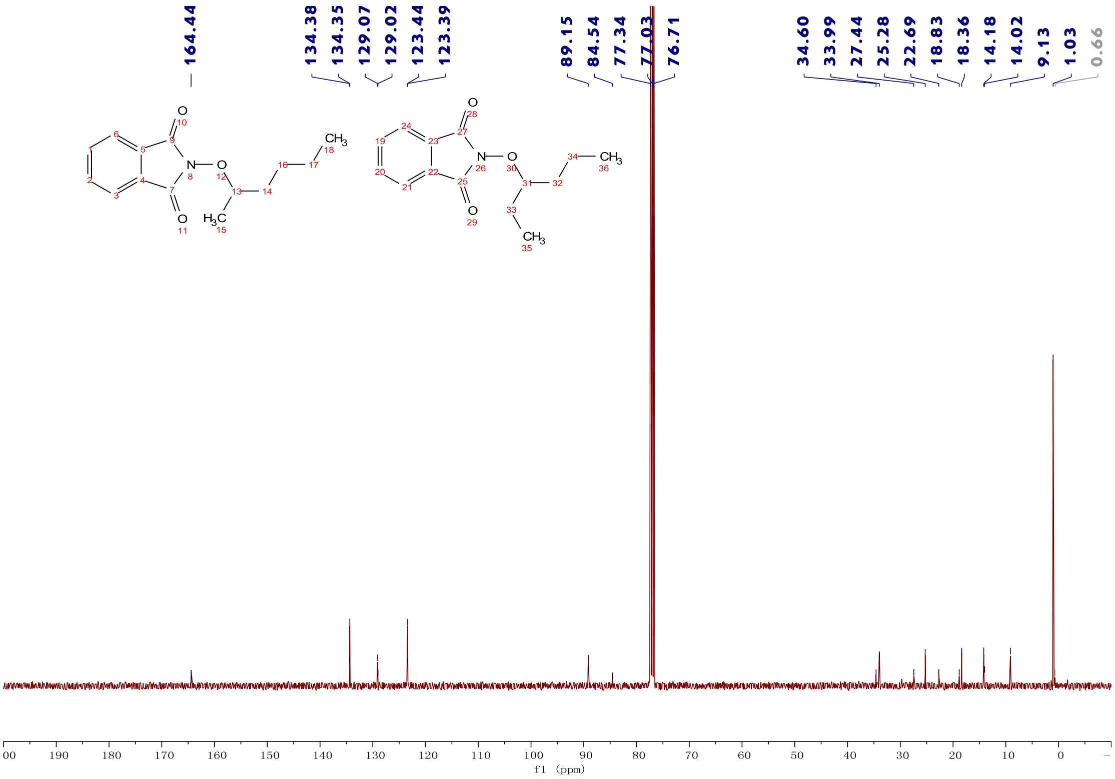

Supplementary Figure 14. ^{13}C NMR Spectra of **9**

10 (^{1}H, CDCl3)

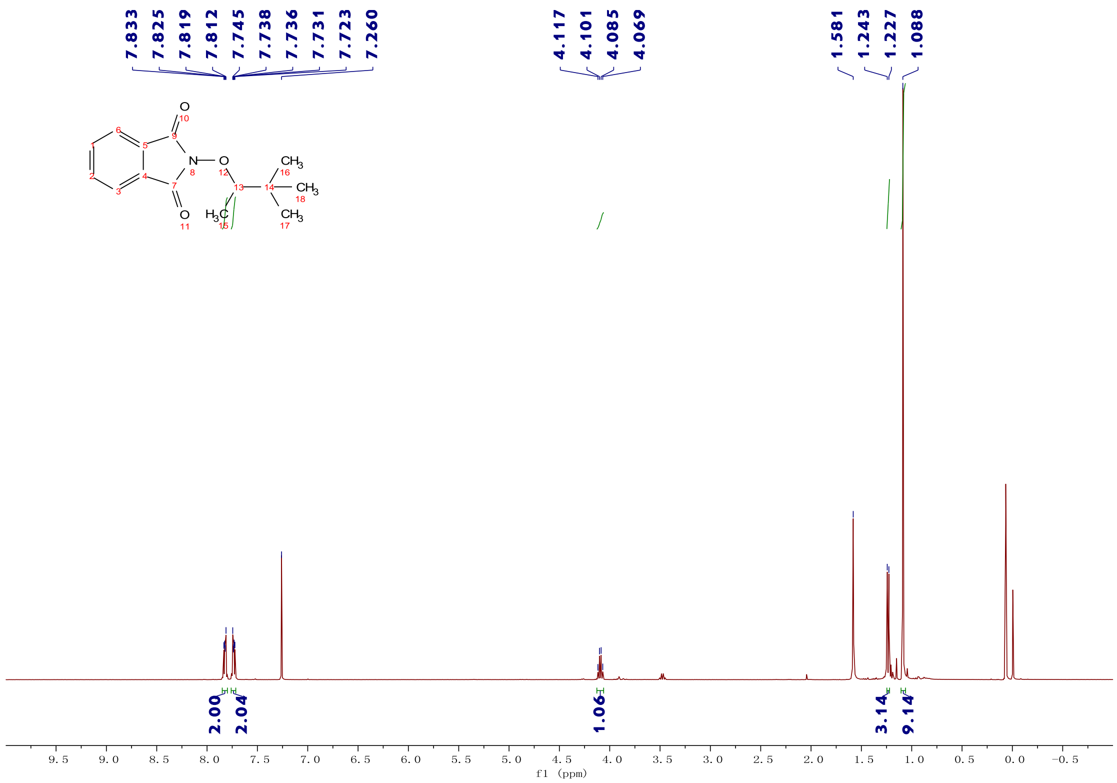

Supplementary Figure 15. ^{1}H NMR Spectra of **10**

10 (^{13}C, CDCl3)

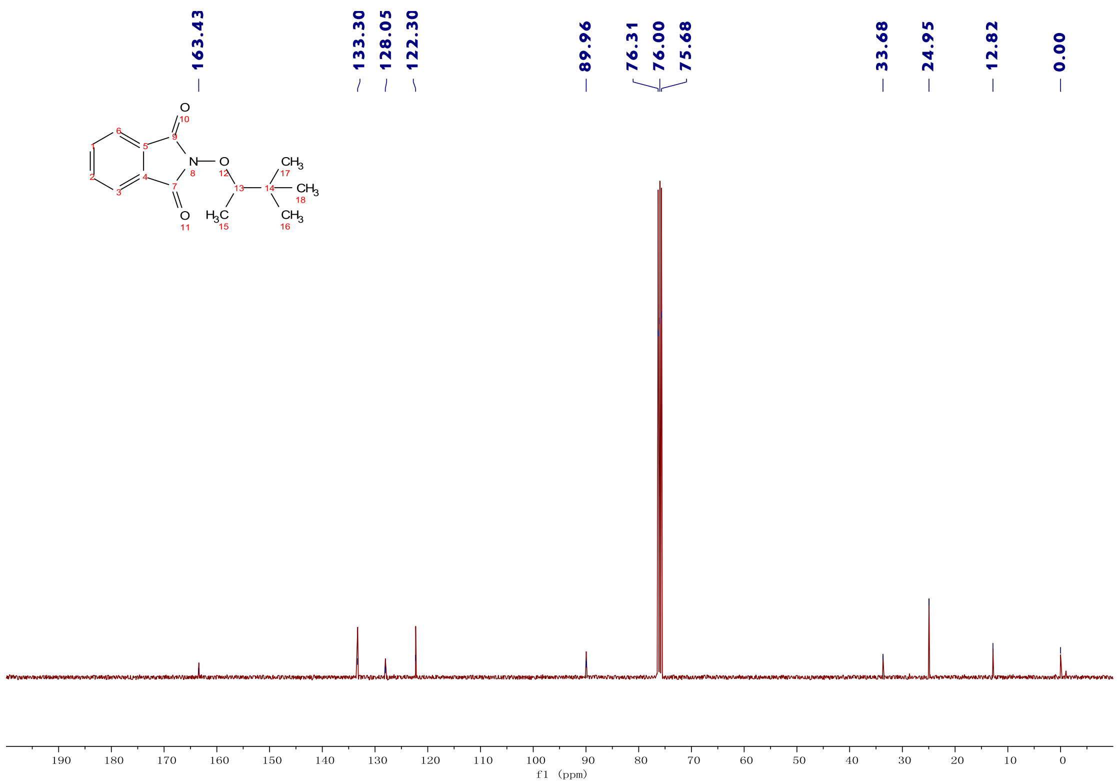

Supplementary Figure 16. ^{13}C NMR Spectra of **10**

11 (^{1}H, CDCl3)

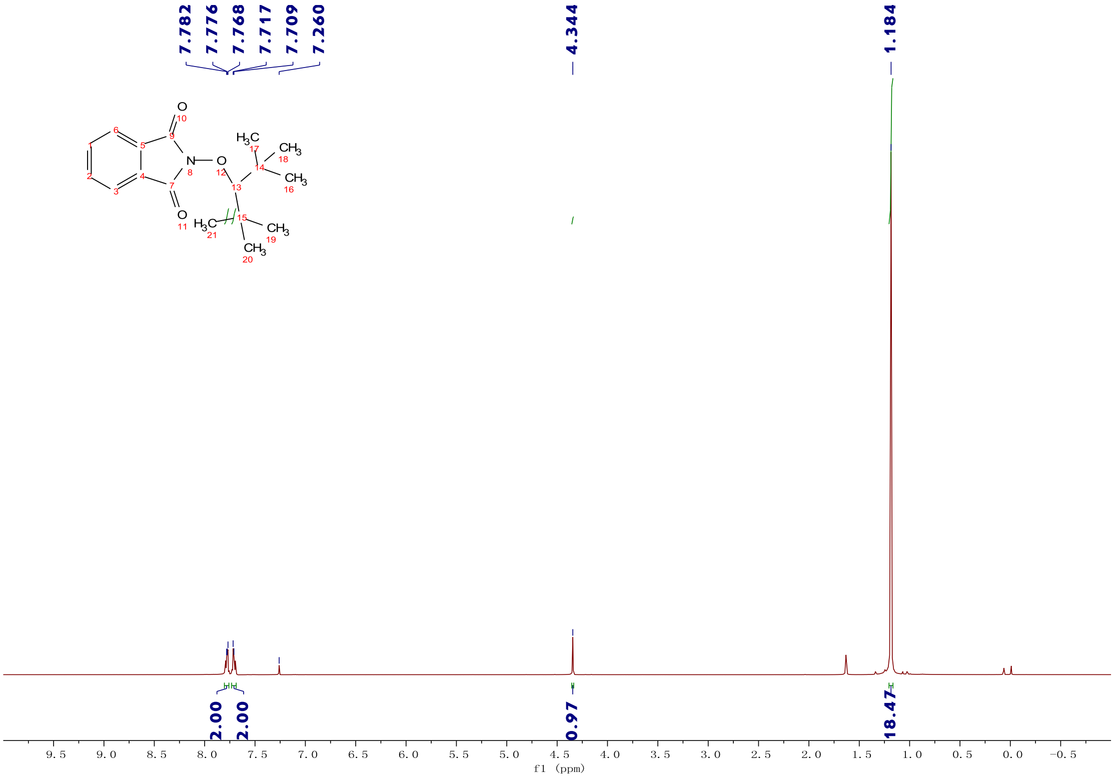

Supplementary Figure 17. ^{1}H NMR Spectra of **11**

11 (^{13}C, CDCl3)

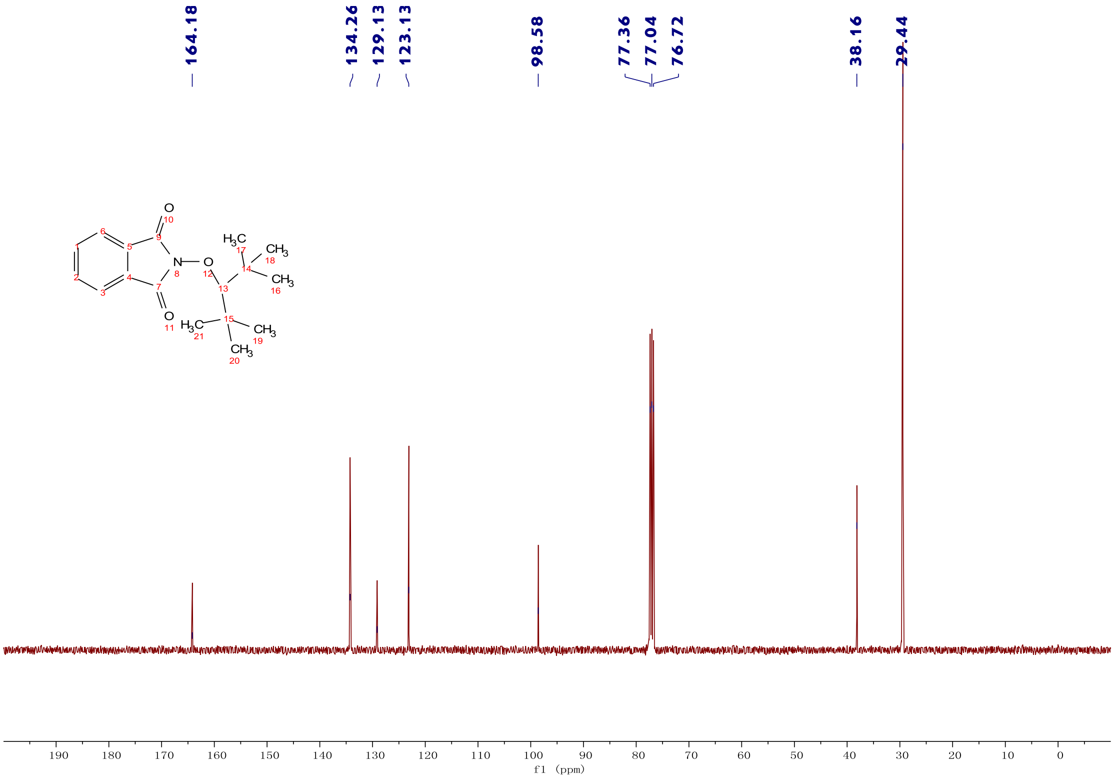

Supplementary Figure 18. ^{13}C NMR Spectra of **11**

12 (^{1}H, $CDCl_3$)

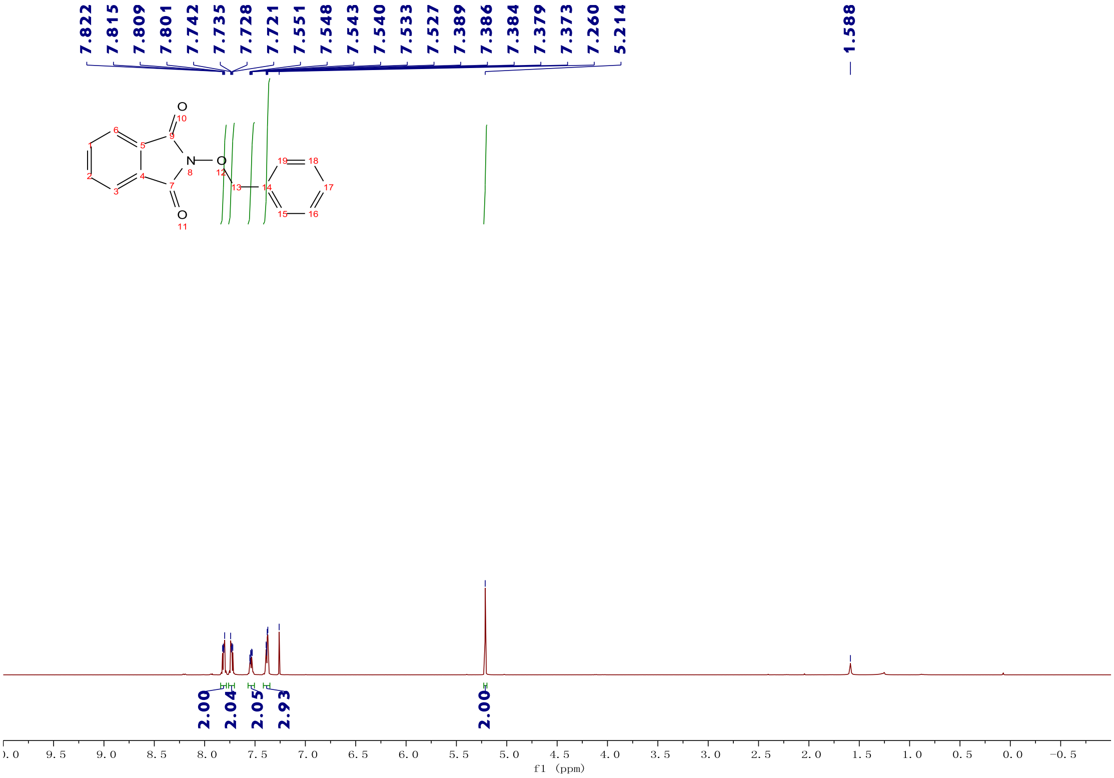

Supplementary Figure 19. ^{1}H NMR Spectra of **12**

12 (^{13}C, $CDCl_3$)

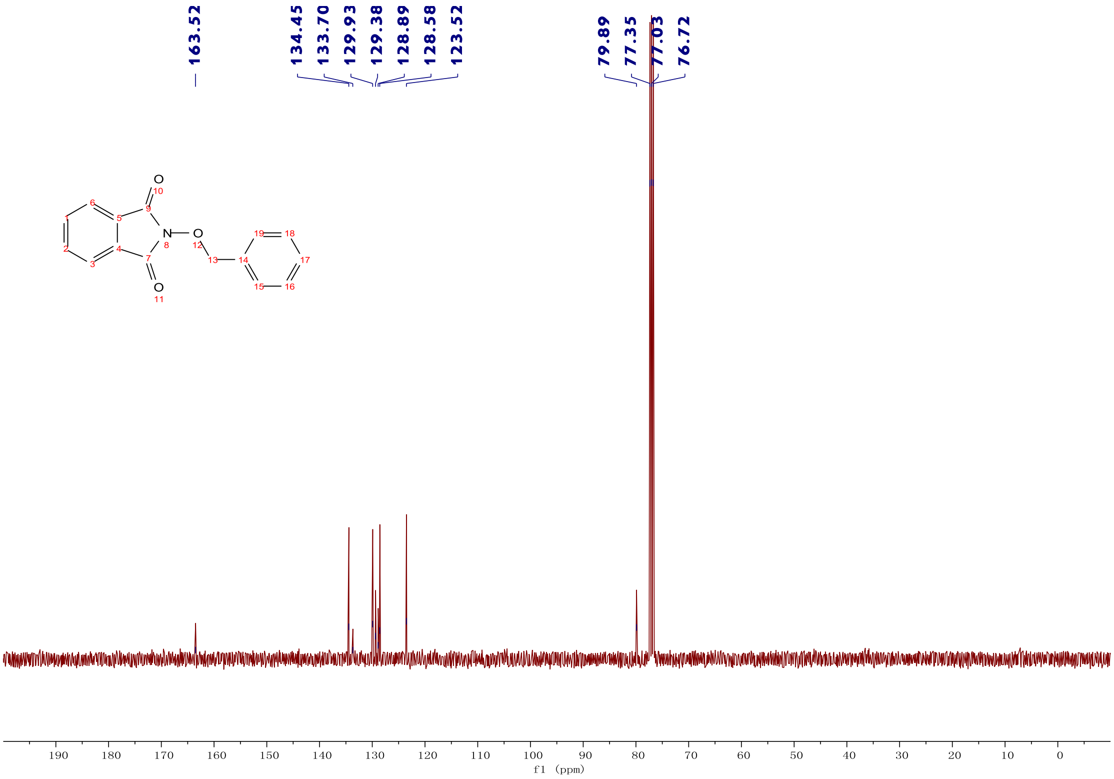

Supplementary Figure 20. ^{13}C NMR Spectra of **12**

13 (^{1}H, $CDCl_3$)

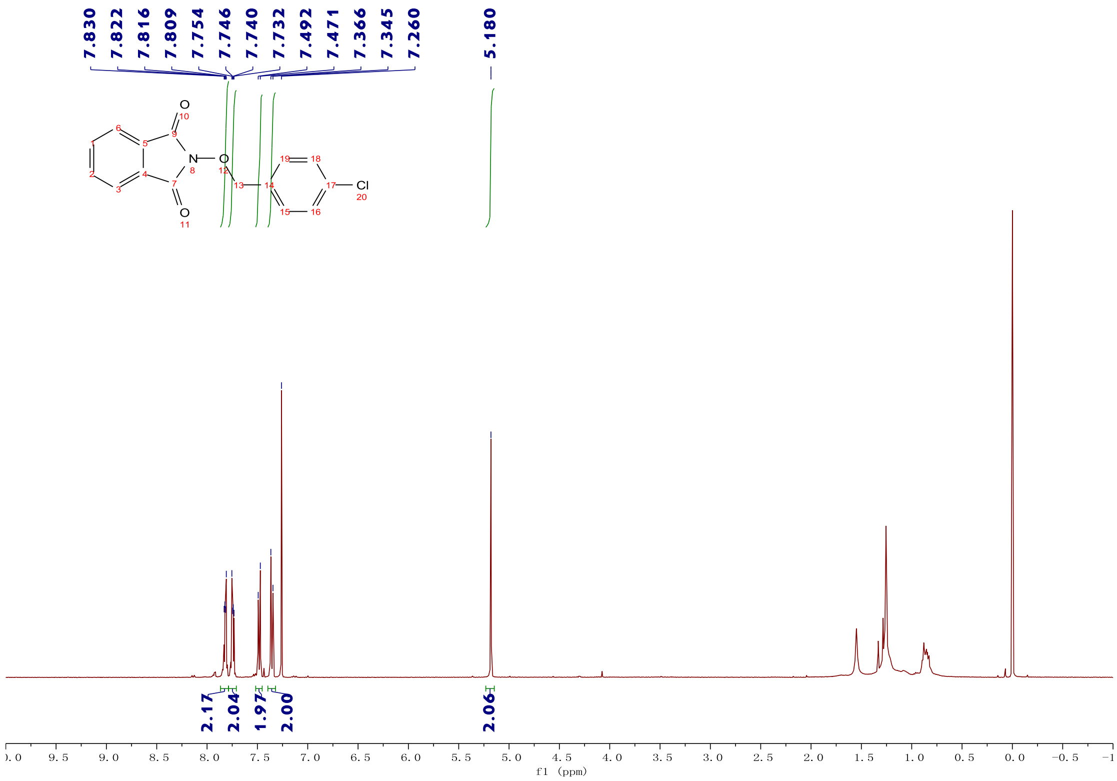

Supplementary Figure 21. ^{1}H NMR Spectra of **13**

13 (^{13}C, $CDCl_3$)

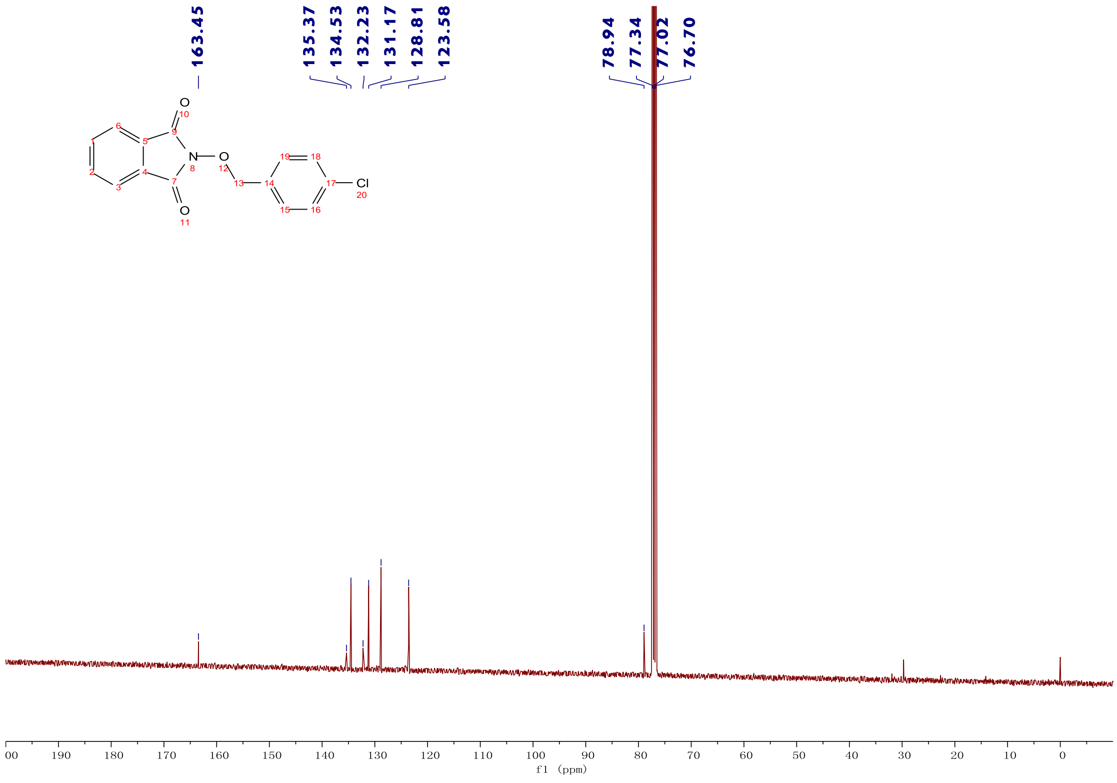

Supplementary Figure 22. ^{13}C NMR Spectra of **13**

14 (^{1}H, $CDCl_3$)

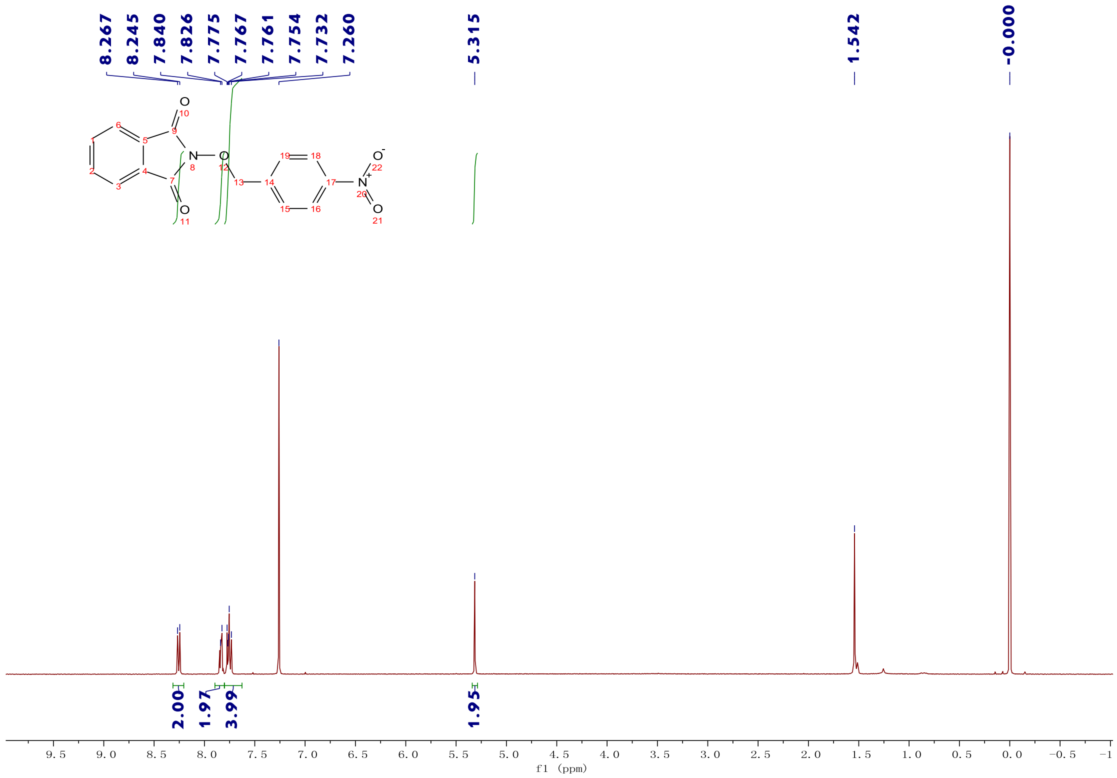

Supplementary Figure 23. ^{1}H NMR Spectra of **14**

14 (^{13}C, $CDCl_3$)

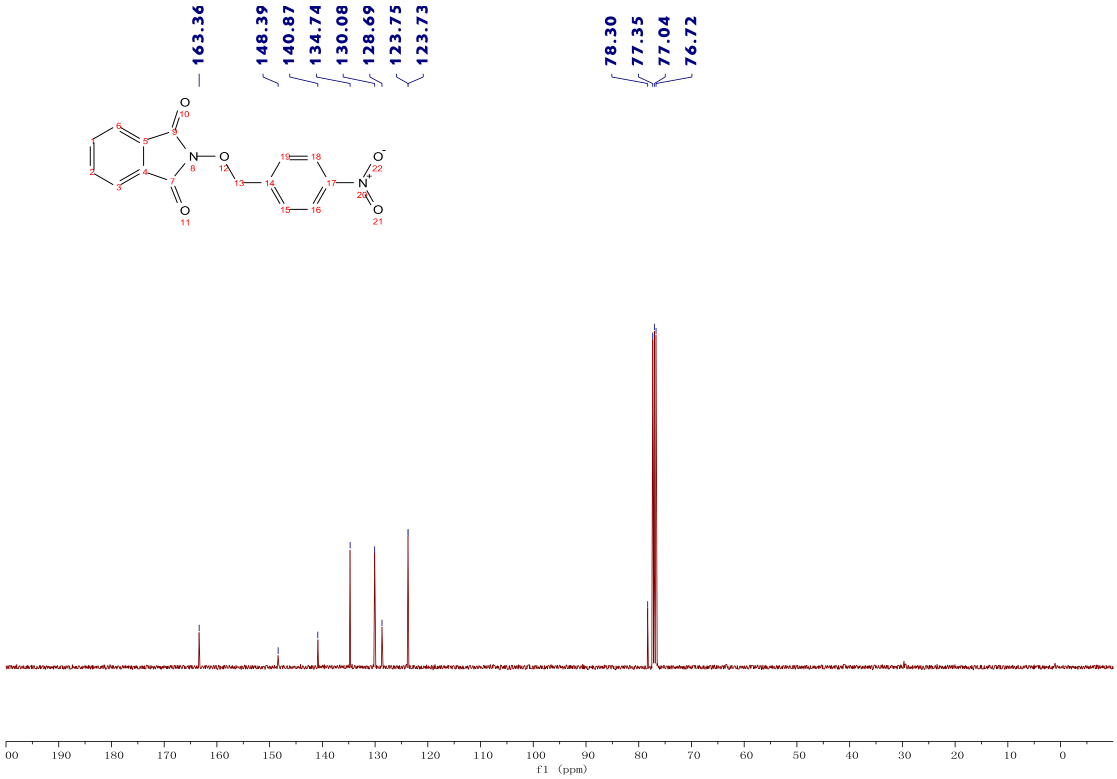

Supplementary Figure 24. ^{13}C NMR Spectra of **14**

15 (^{1}H, CDCl3)

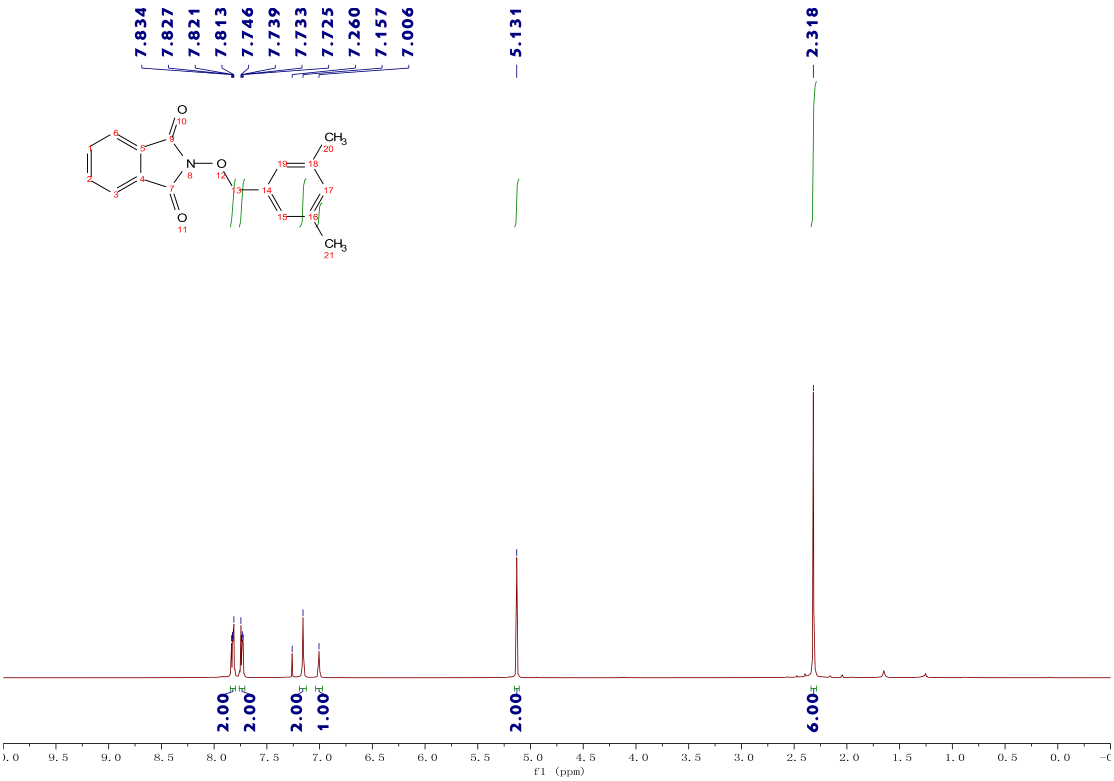

Supplementary Figure 25. ^{1}H NMR Spectra of **15**

15 (^{13}C, CDCl3)

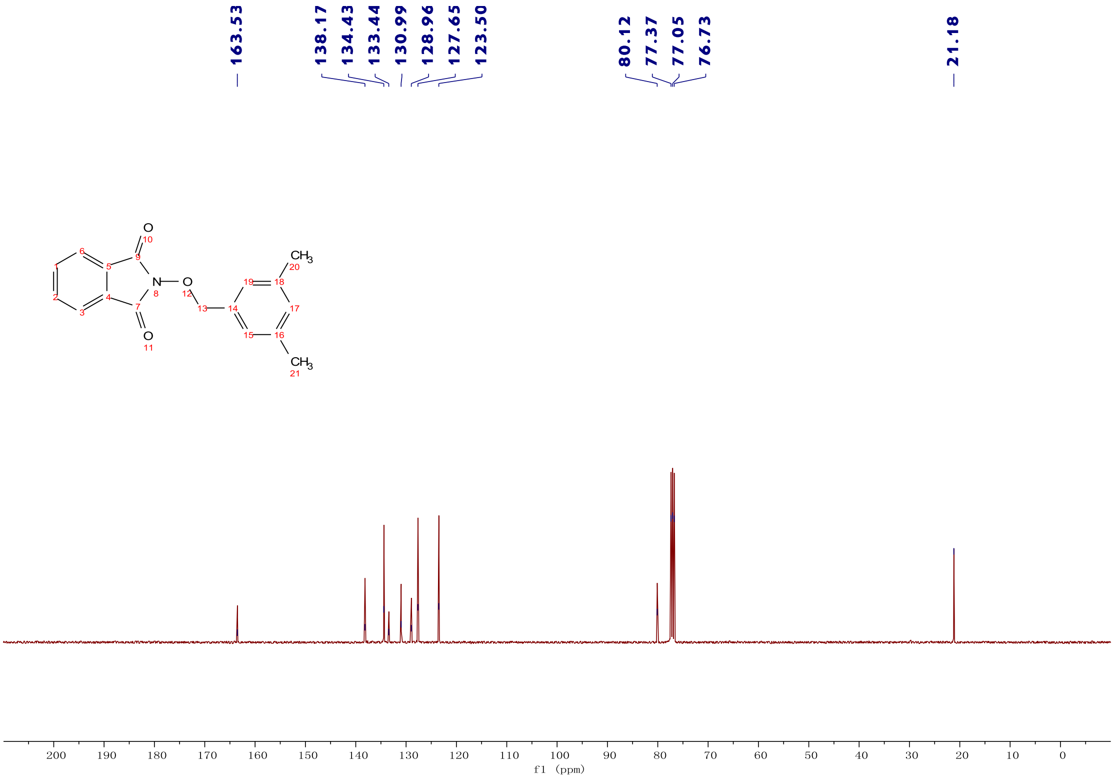

Supplementary Figure 26. ^{13}C NMR Spectra of **15**

16 (^{1}H, $CDCl_3$)

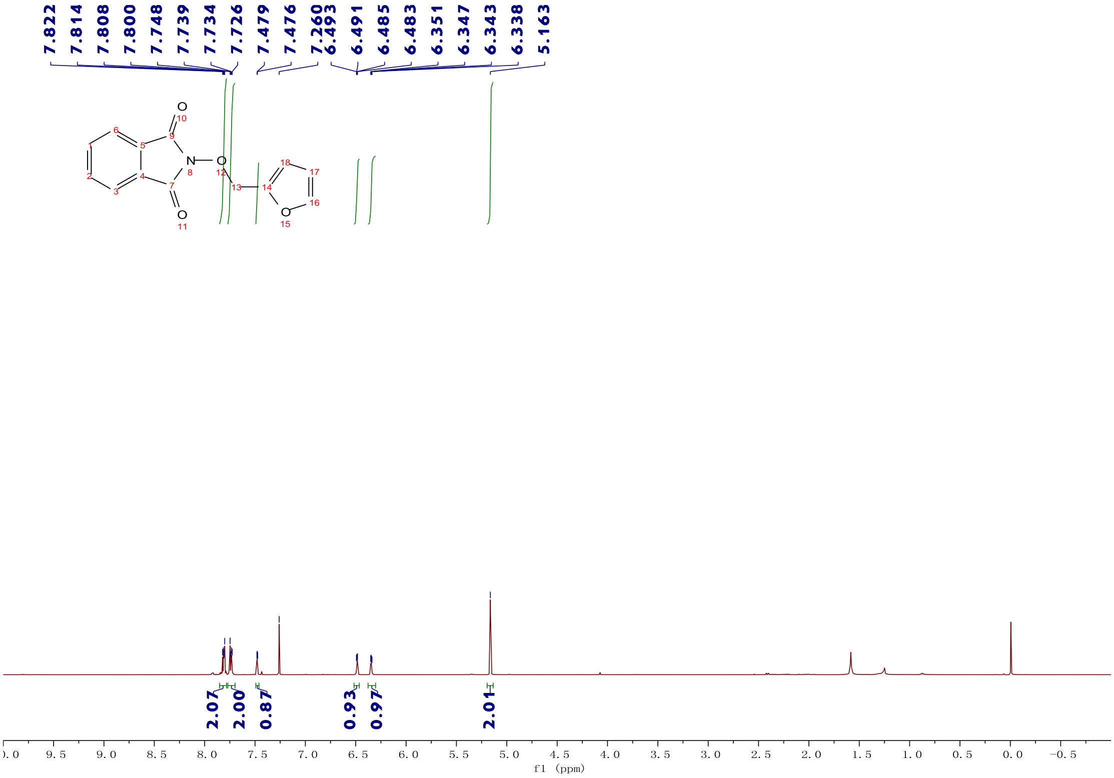

Supplementary Figure 27. ^{1}H NMR Spectra of **16**

16 (^{13}C, $CDCl_3$)

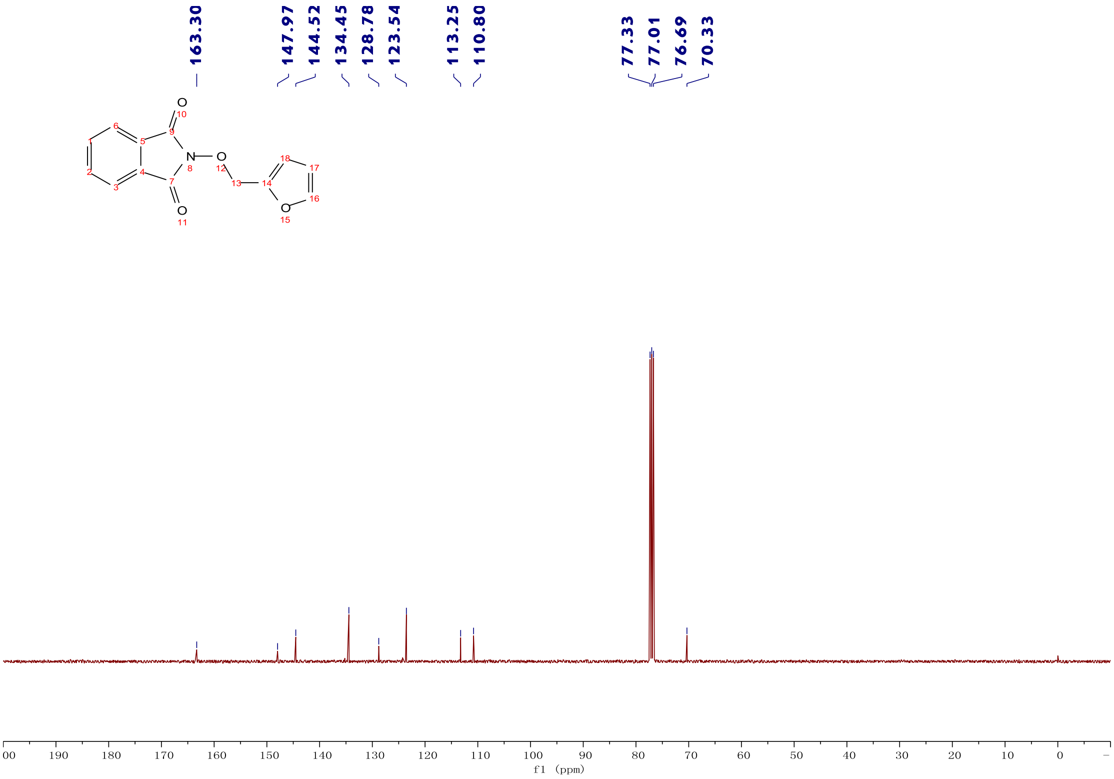

Supplementary Figure 28. ^{13}C NMR Spectra of **16**

17 (^{1}H, $CDCl_3$)

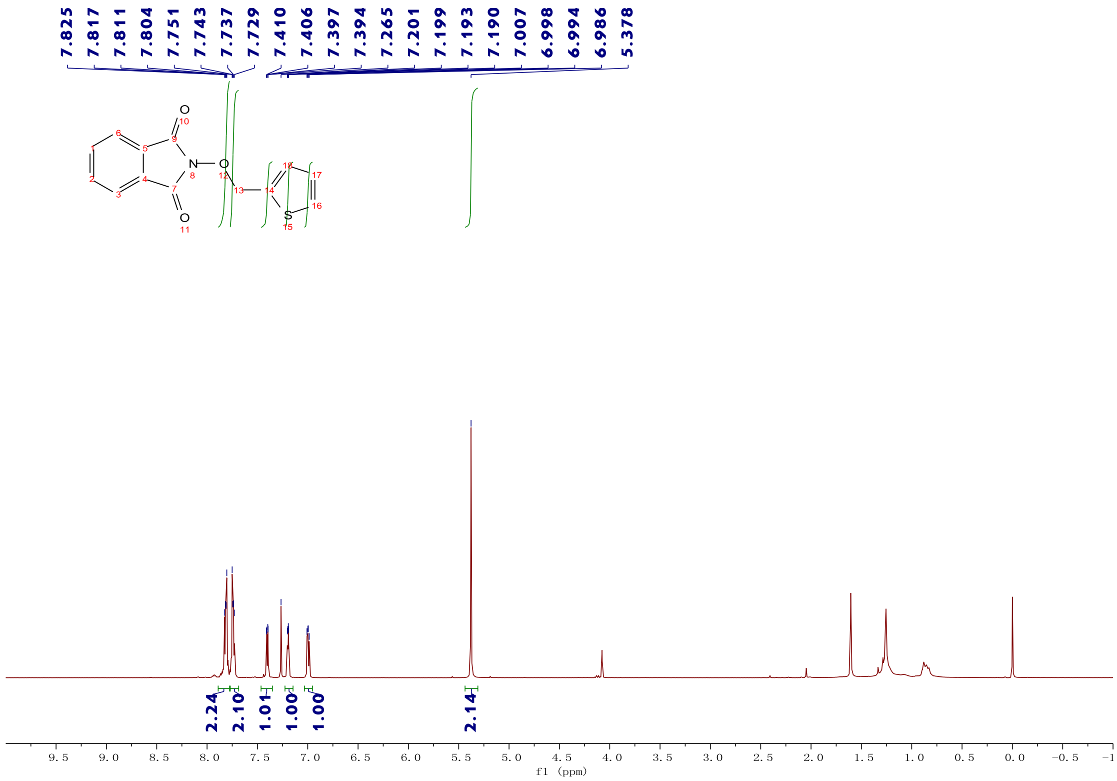

Supplementary Figure 29. ^{1}H NMR Spectra of **17**

17 (^{13}C, $CDCl_3$)

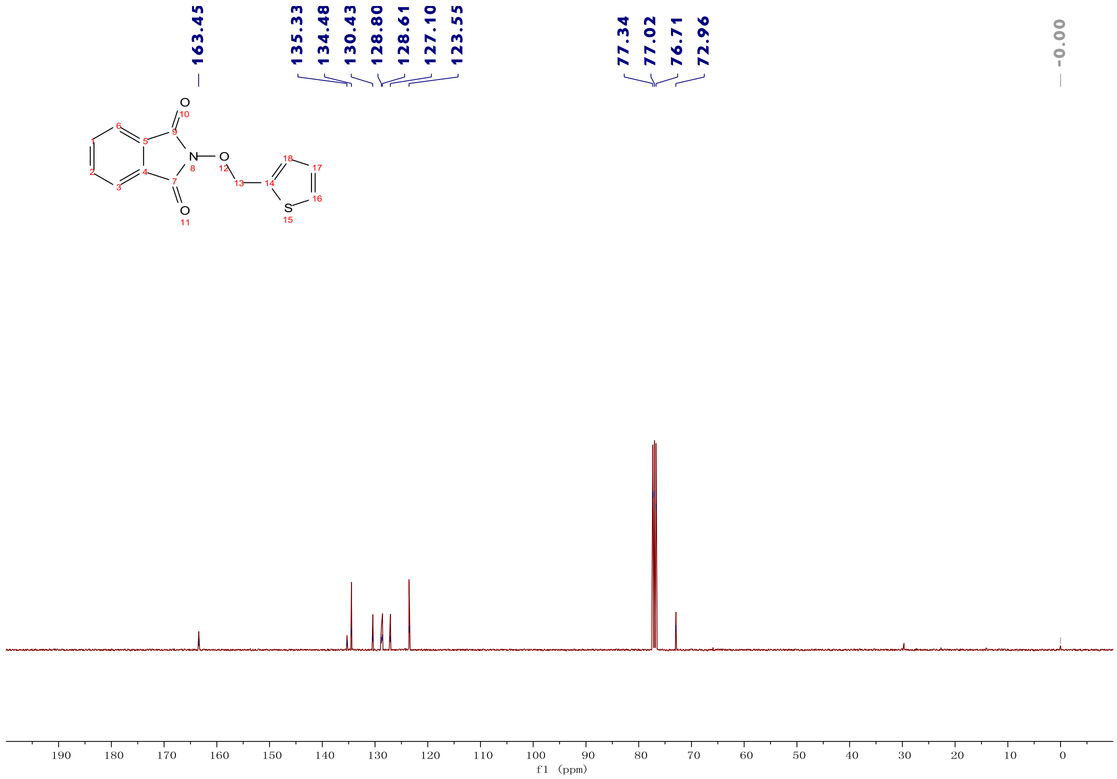

Supplementary Figure 30. ^{13}C NMR Spectra of **17**

18 (^{1}H, $CDCl_3$)

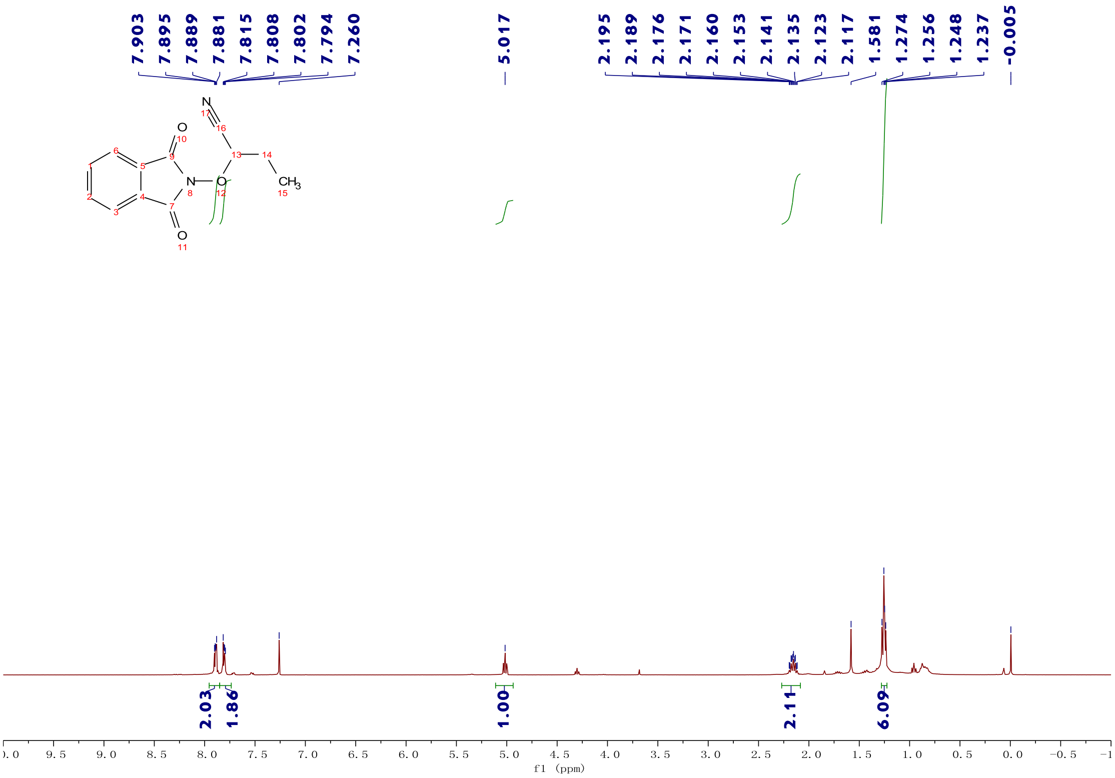

Supplementary Figure 31. ^{1}H NMR Spectra of **18**

18 (^{13}C, $CDCl_3$)

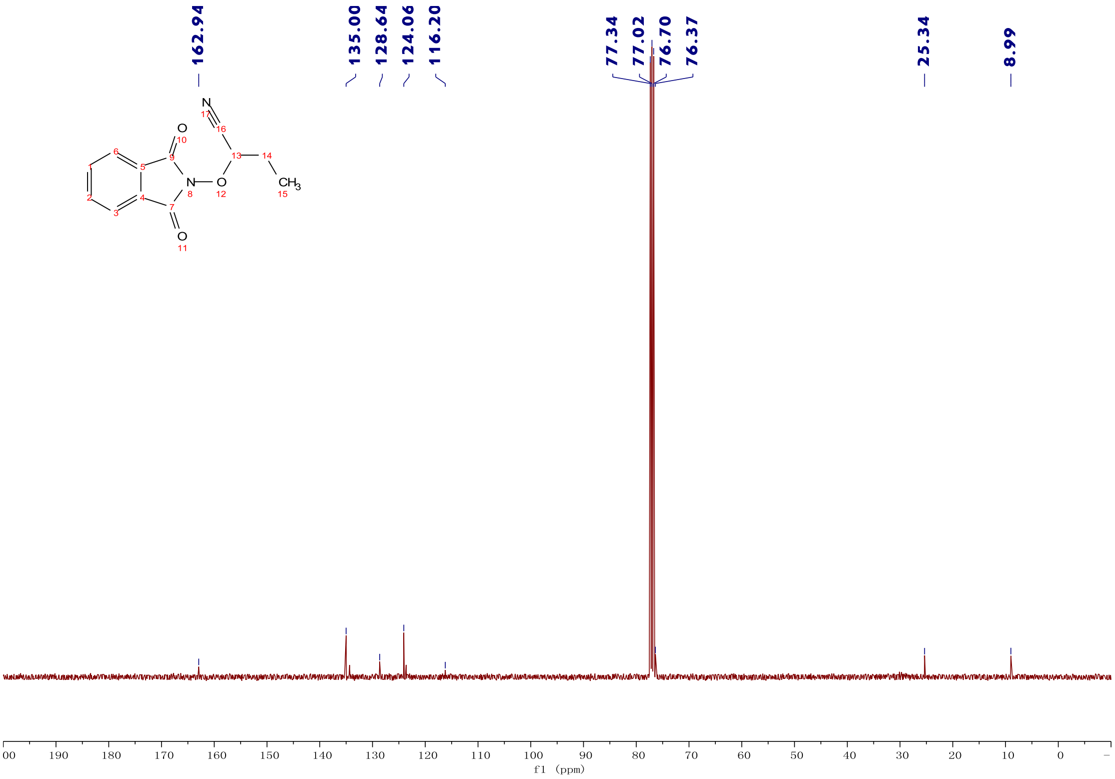

Supplementary Figure 32. ^{13}C NMR Spectra of **18**

19 (^{1}H, CDCl$_3$)

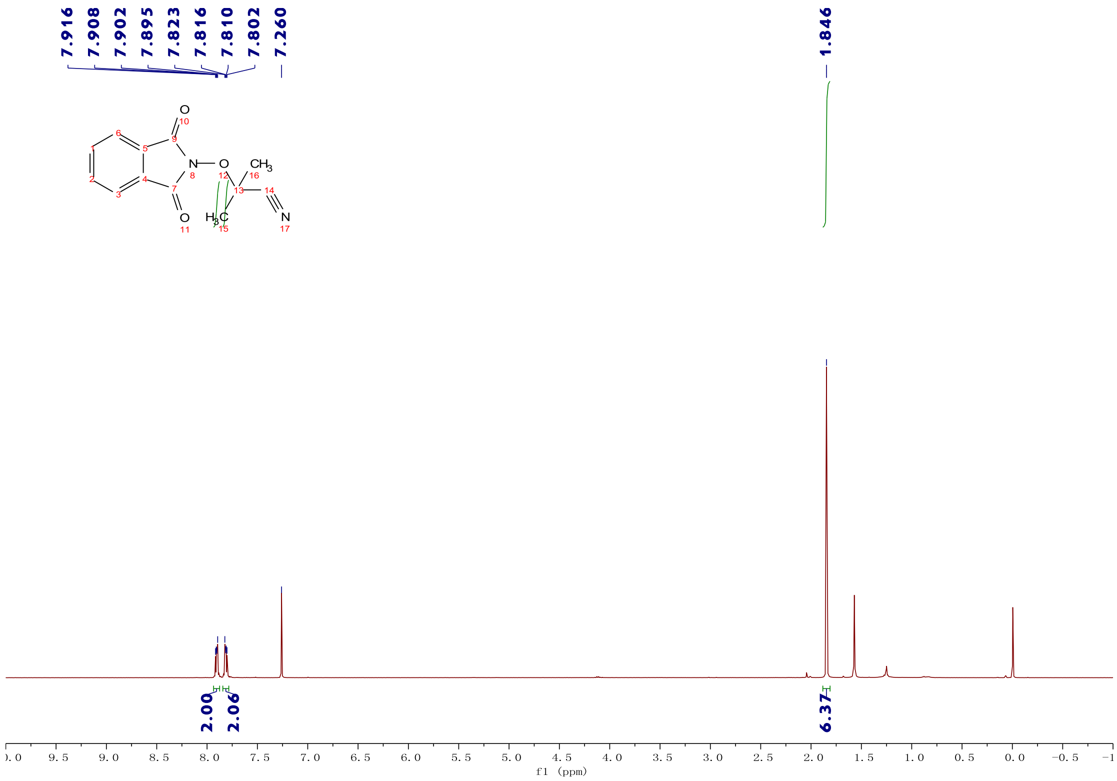

Supplementary Figure 33. ^{1}H NMR Spectra of **19**

19 (^{13}C, CDCl$_3$)

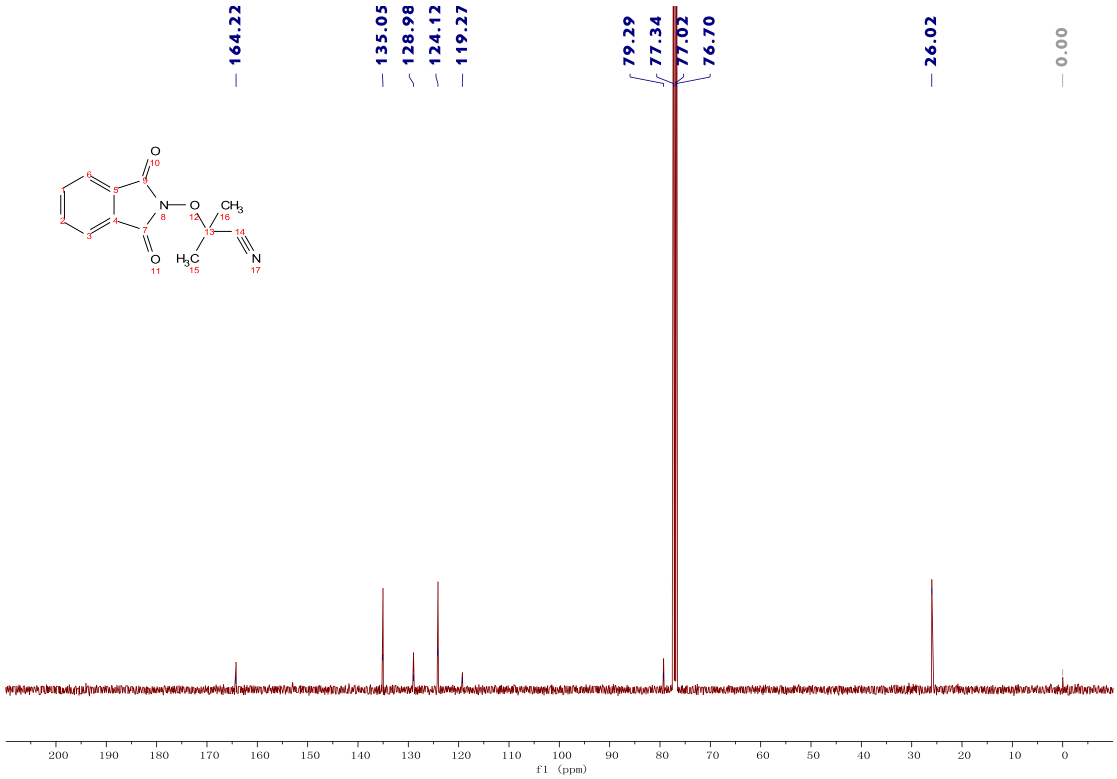

Supplementary Figure 34. ^{13}C NMR Spectra of **19**

20 (^{1}H, $CDCl_3$)

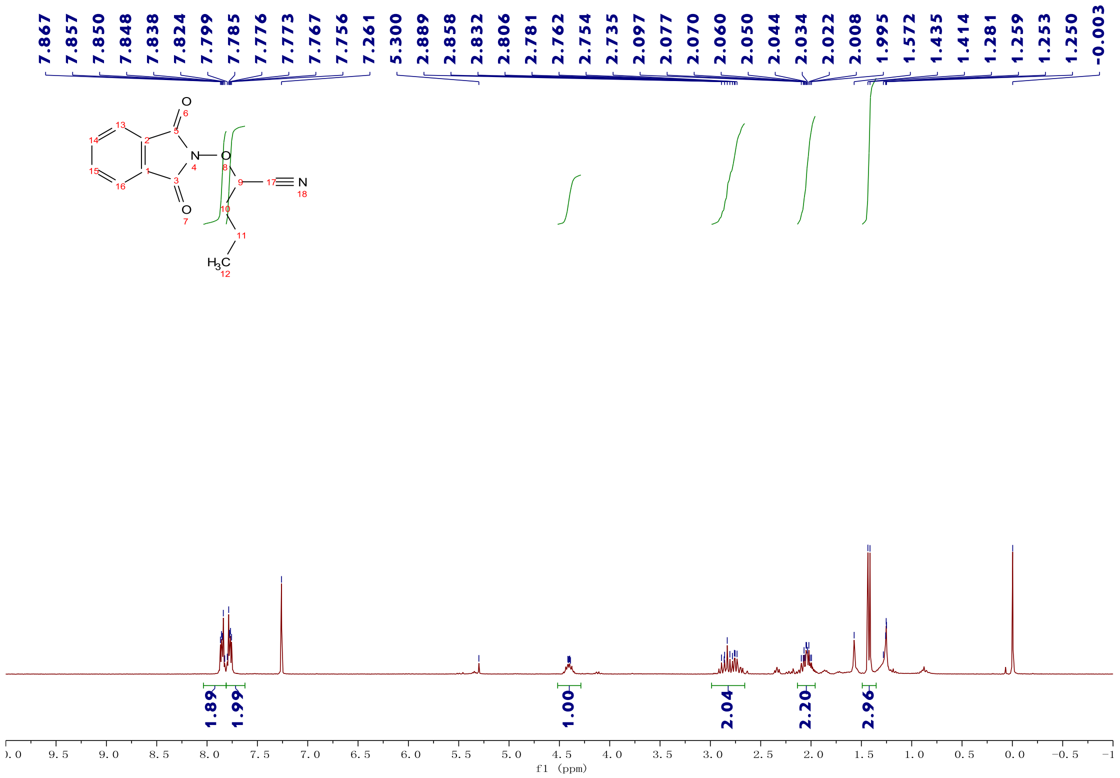

Supplementary Figure 35. ^{1}H NMR Spectra of **20**

20 (^{13}C, $CDCl_3$)

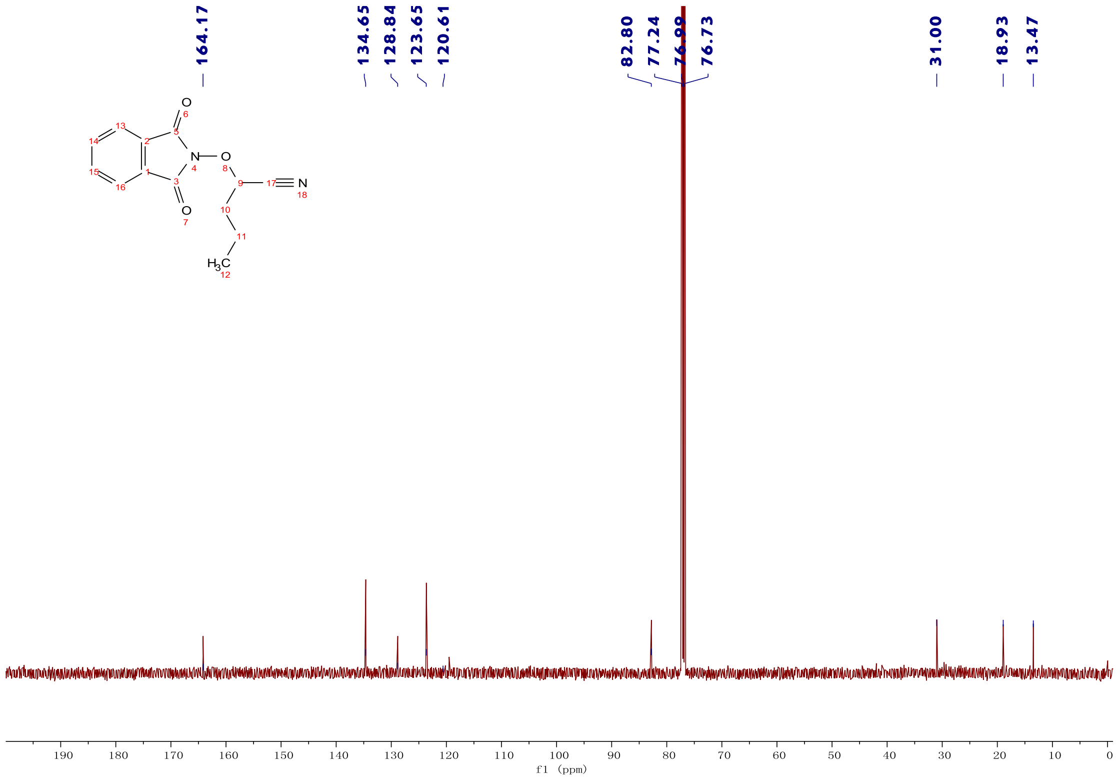

Supplementary Figure 36. ^{13}C NMR Spectra of **20**

21 (^1H, $CDCl_3$)

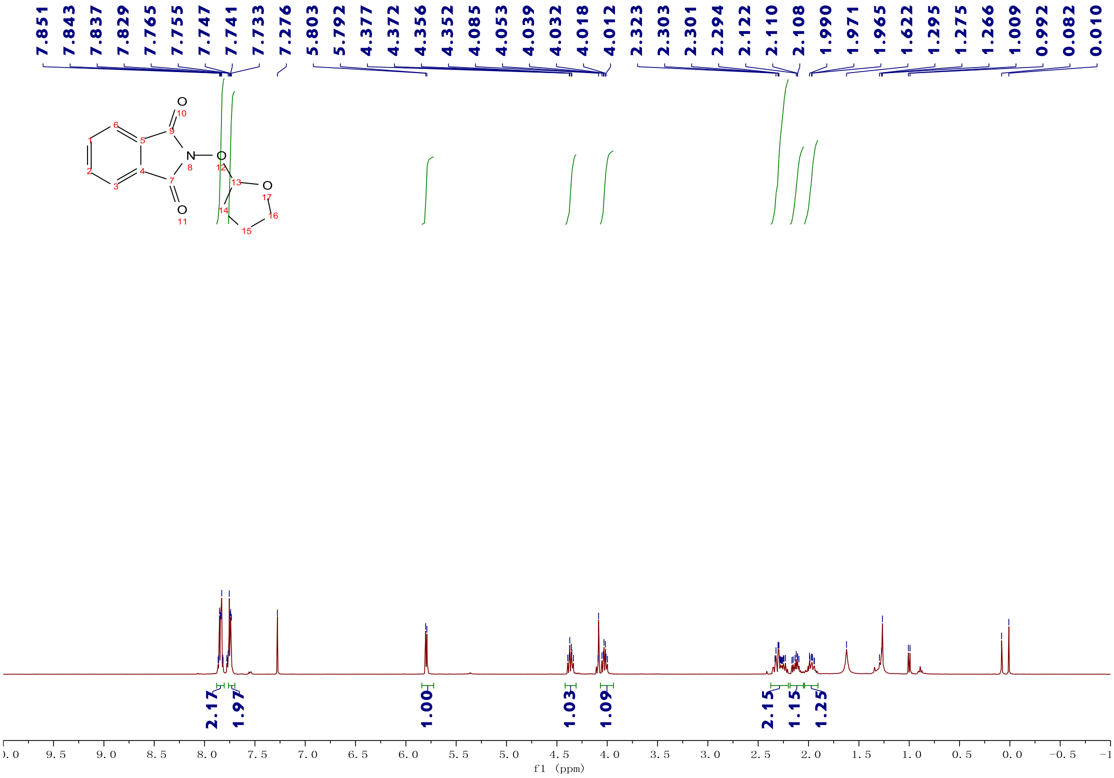

Supplementary Figure 37. ^1H NMR Spectra of **21**

21 (^{13}C, $CDCl_3$)

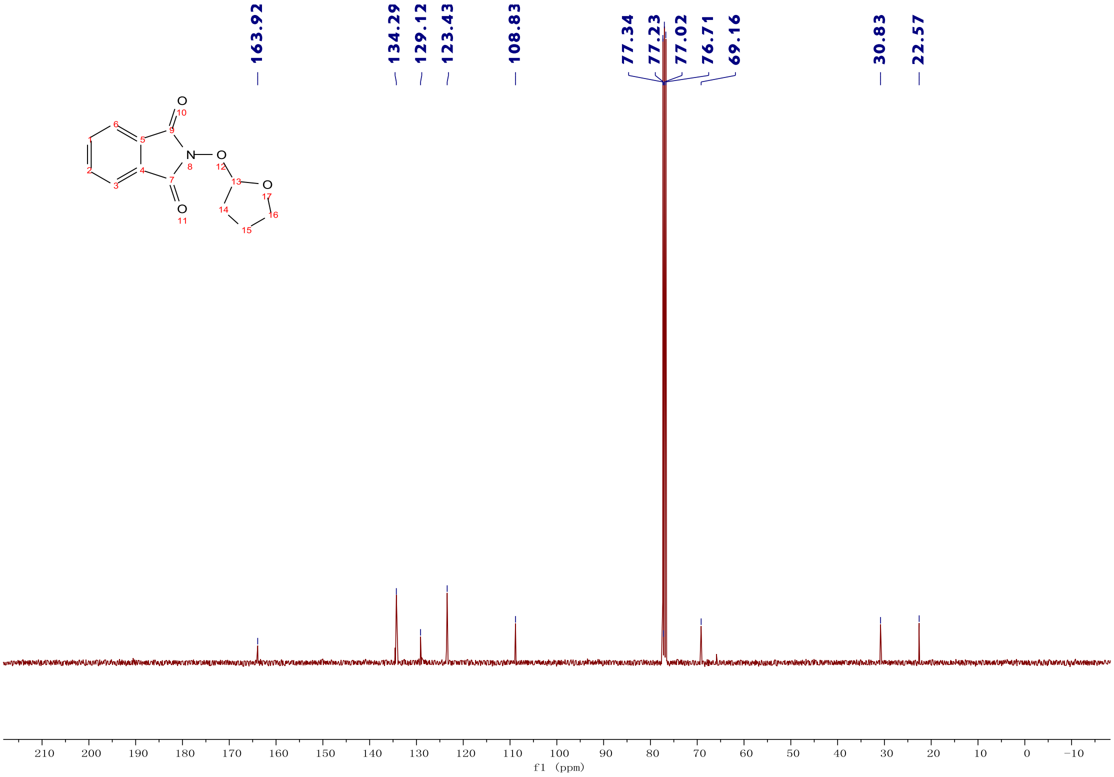

Supplementary Figure 38. ^{13}C NMR Spectra of **21**

22 (^{1}H, $CDCl_3$)

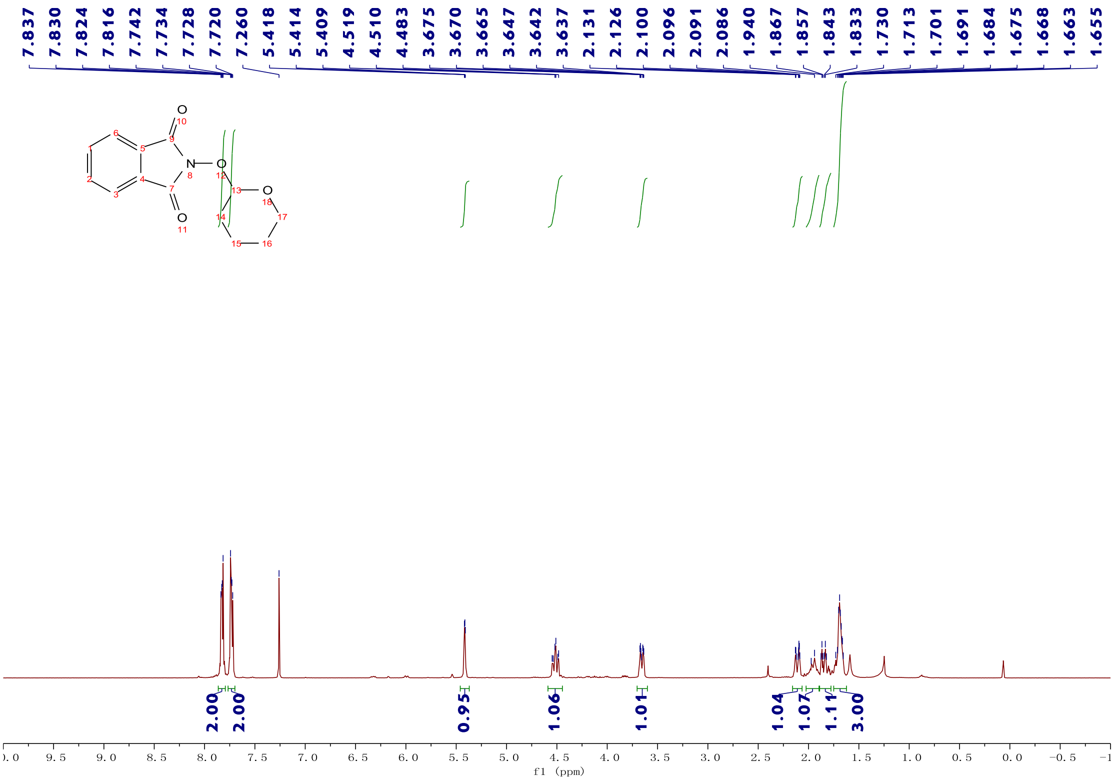

Supplementary Figure 39. ^{1}H NMR Spectra of **22**

22 (^{13}C, $CDCl_3$)

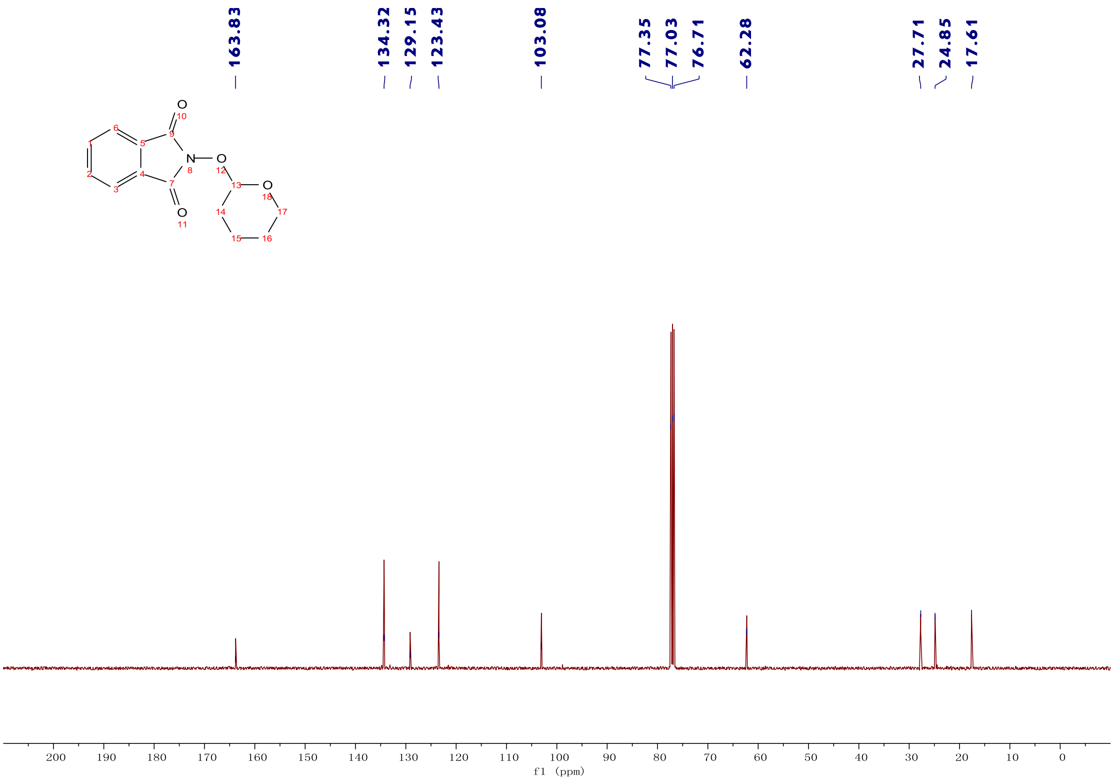

Supplementary Figure 40. ^{13}C NMR Spectra of **22**

23 (^{1}H, CDCl$_3$)

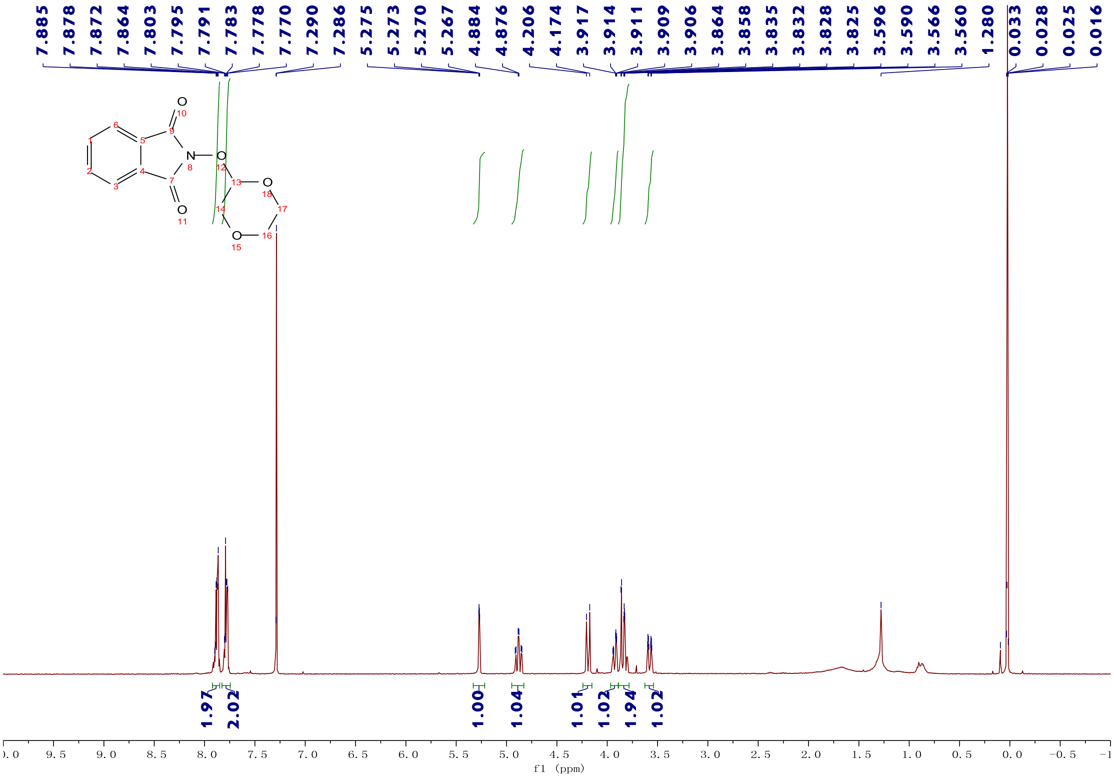

Supplementary Figure 41. ^{1}H NMR Spectra of **23**

23 (^{13}C, CDCl$_3$)

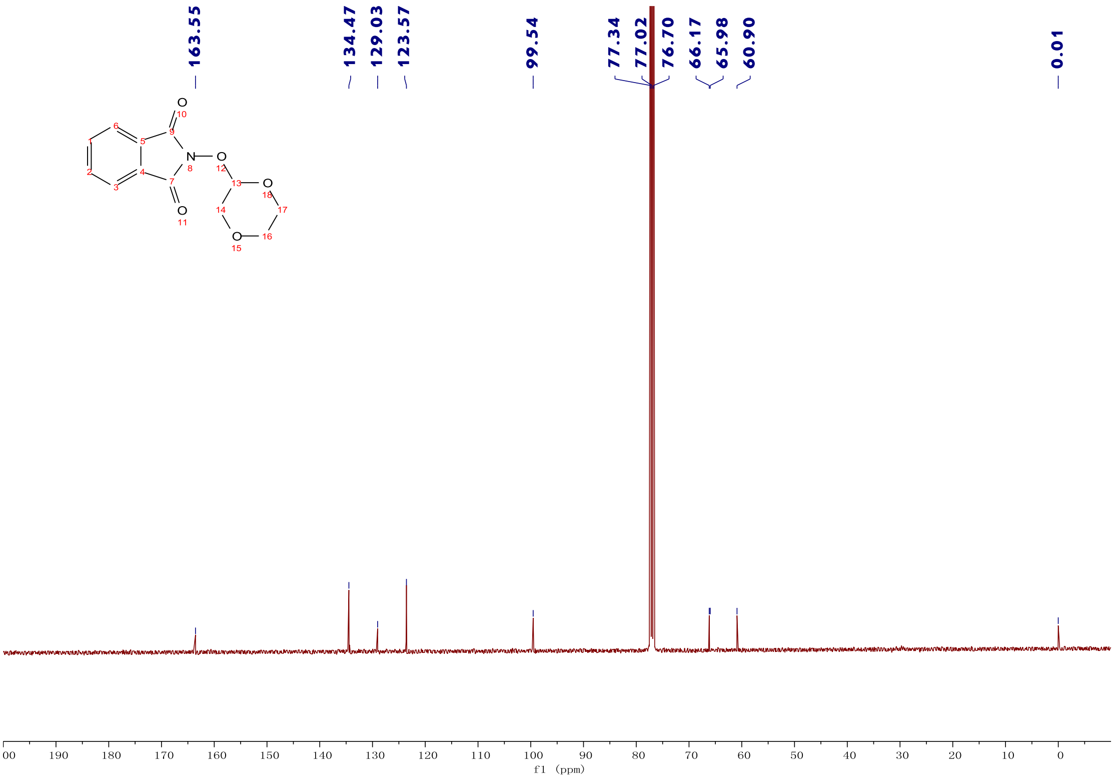

Supplementary Figure 42. ^{13}C NMR Spectra of **23**

24 (^{1}H, $CDCl_3$)

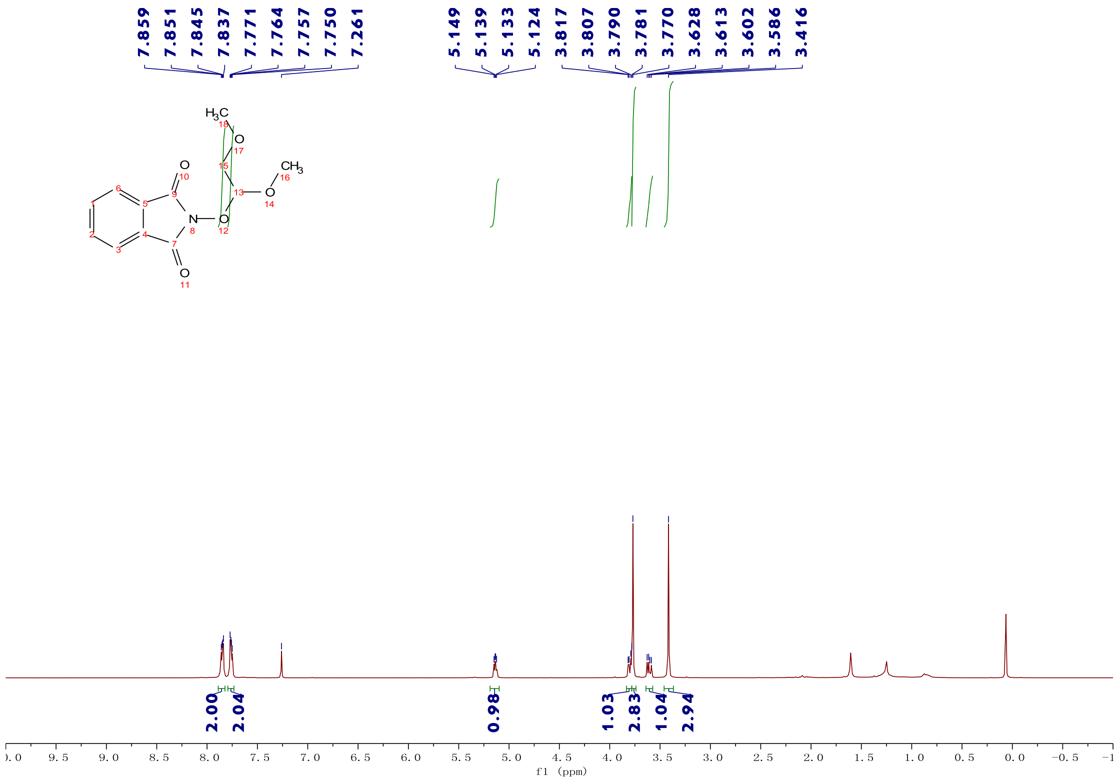

Supplementary Figure 43. ^{1}H NMR Spectra of **24**

24 (^{13}C, $CDCl_3$)

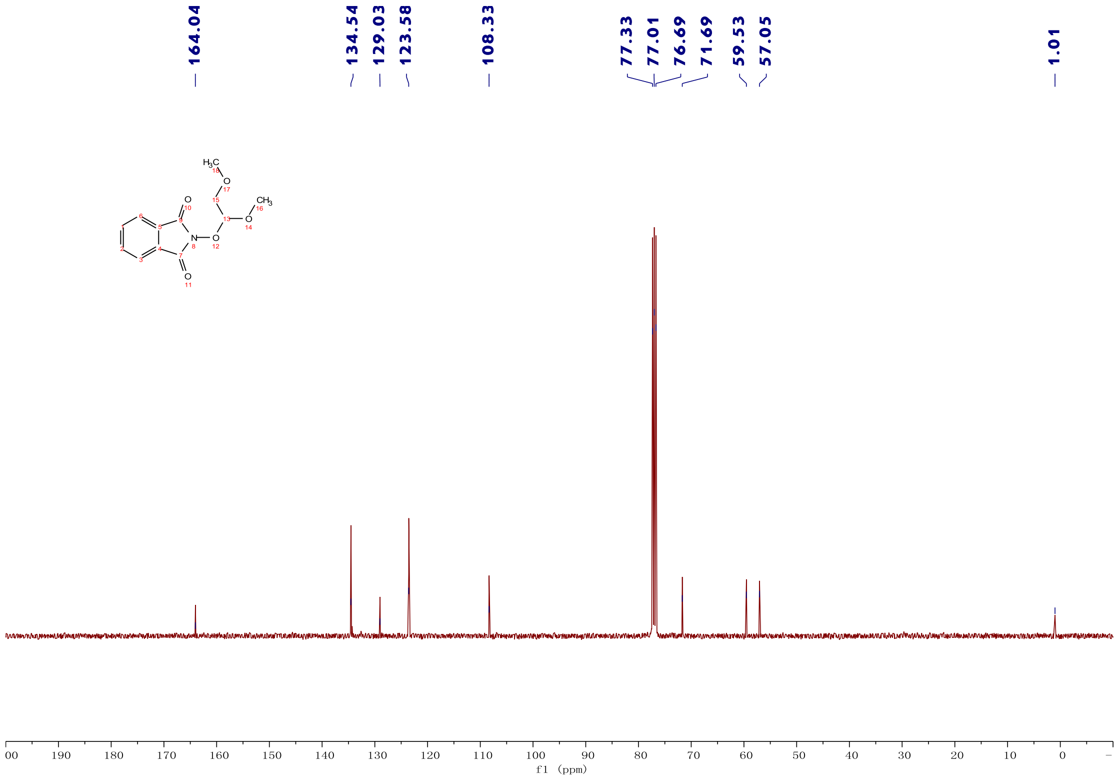

Supplementary Figure 44. ^{13}C NMR Spectra of **24**

25 (^1H, $CDCl_3$)

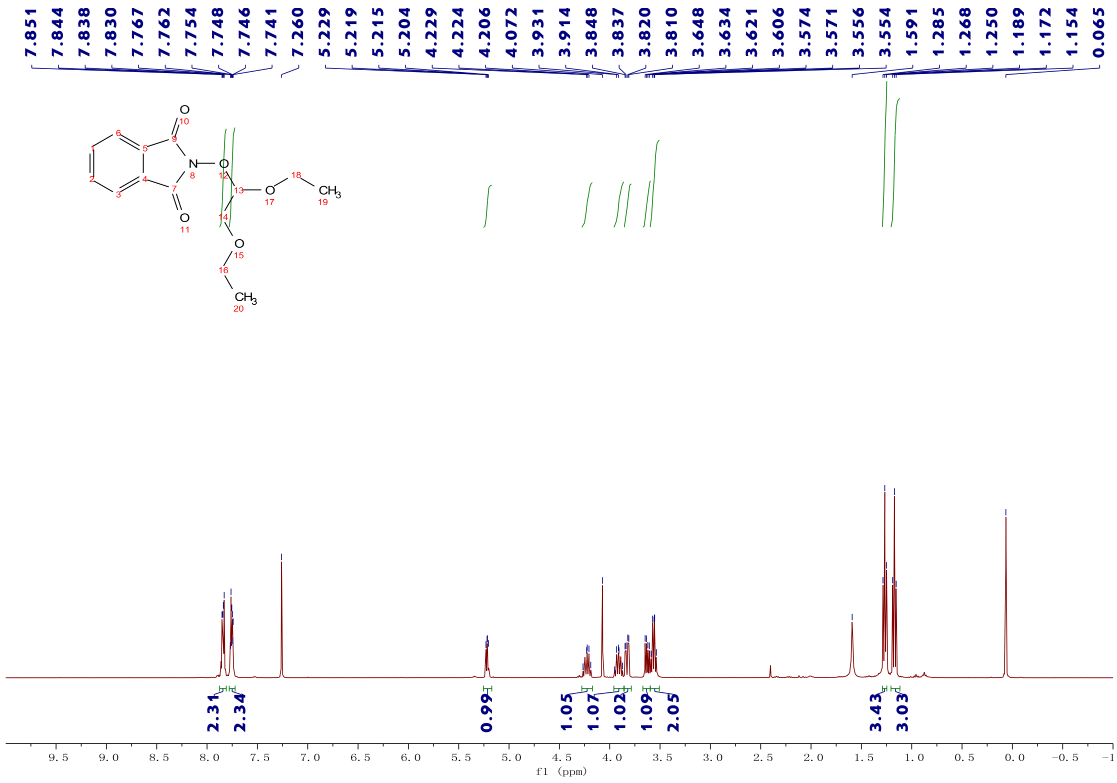

Supplementary Figure 45. ^1H NMR Spectra of **25**

25 (^{13}C, $CDCl_3$)

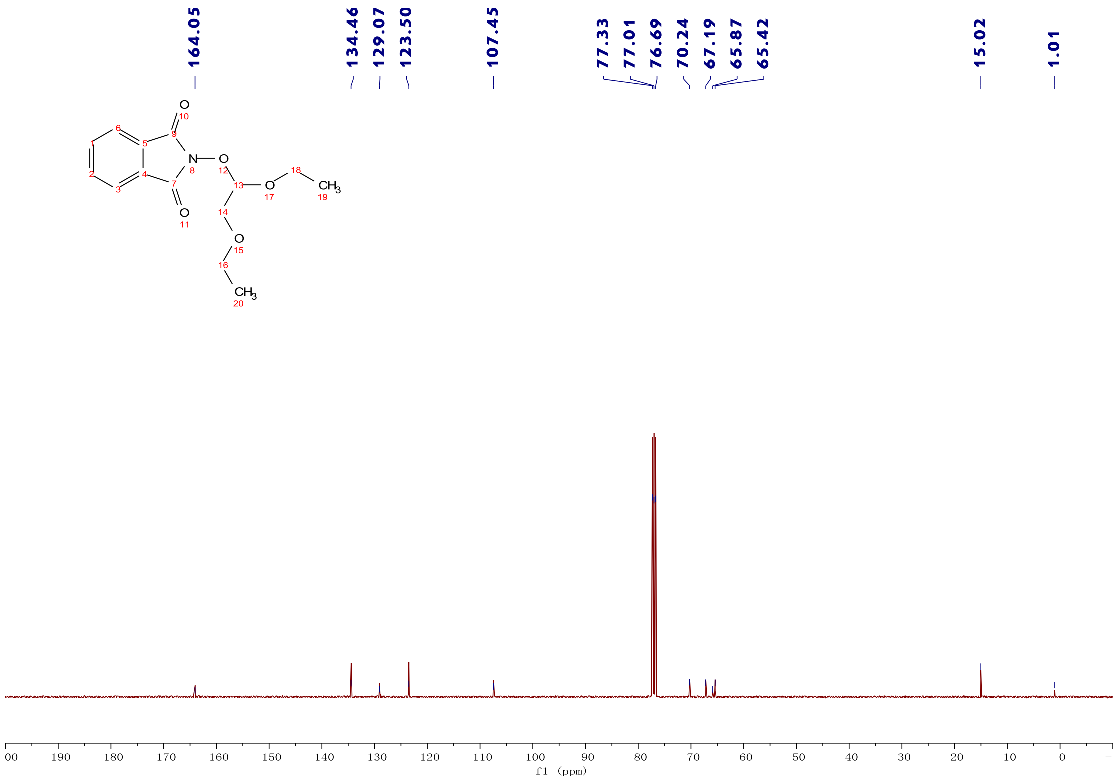

Supplementary Figure 46. ^{13}C NMR Spectra of **25**

26 (^{1}H, $CDCl_3$)

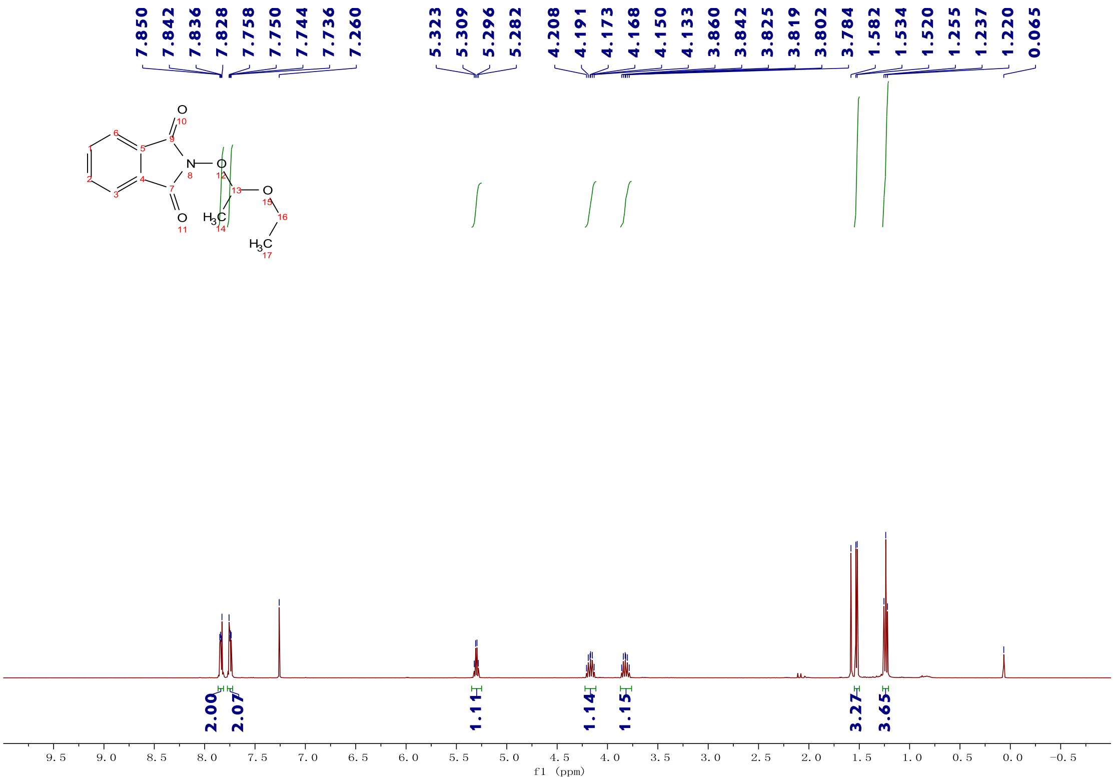

Supplementary Figure 47. ^{1}H NMR Spectra of **26**

26 (^{13}C, $CDCl_3$)

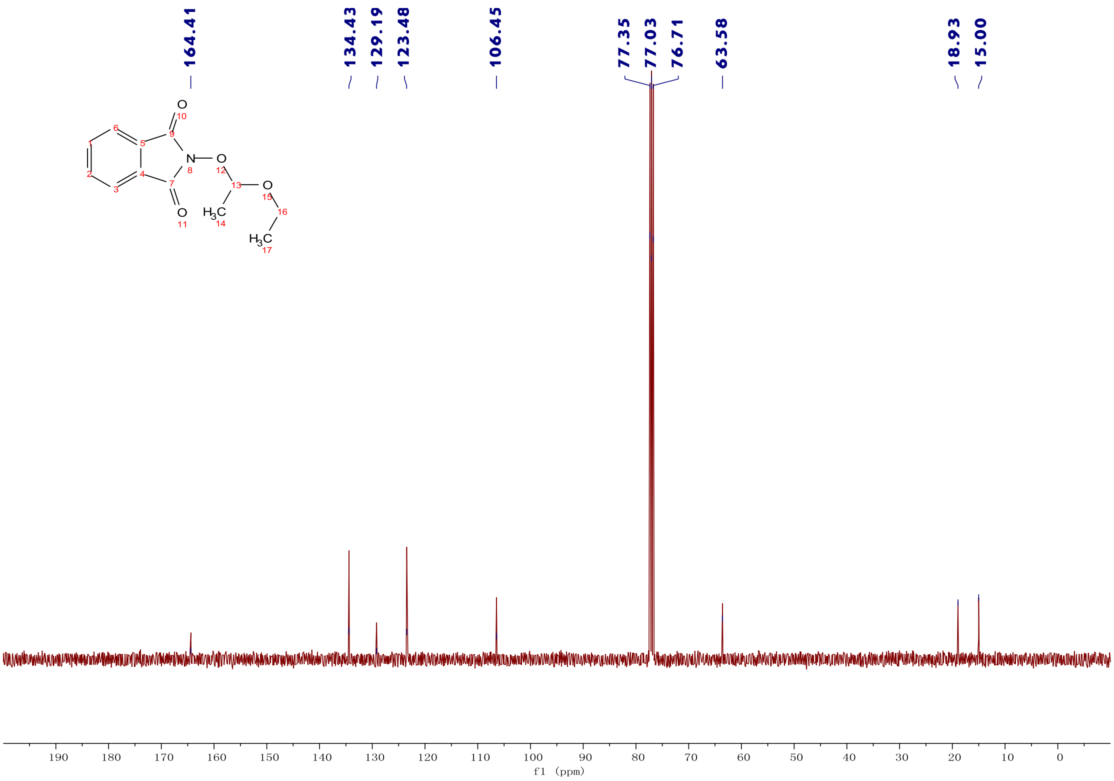

Supplementary Figure 48. ^{13}C NMR Spectra of **26**

27 (^{1}H, $CDCl_3$)

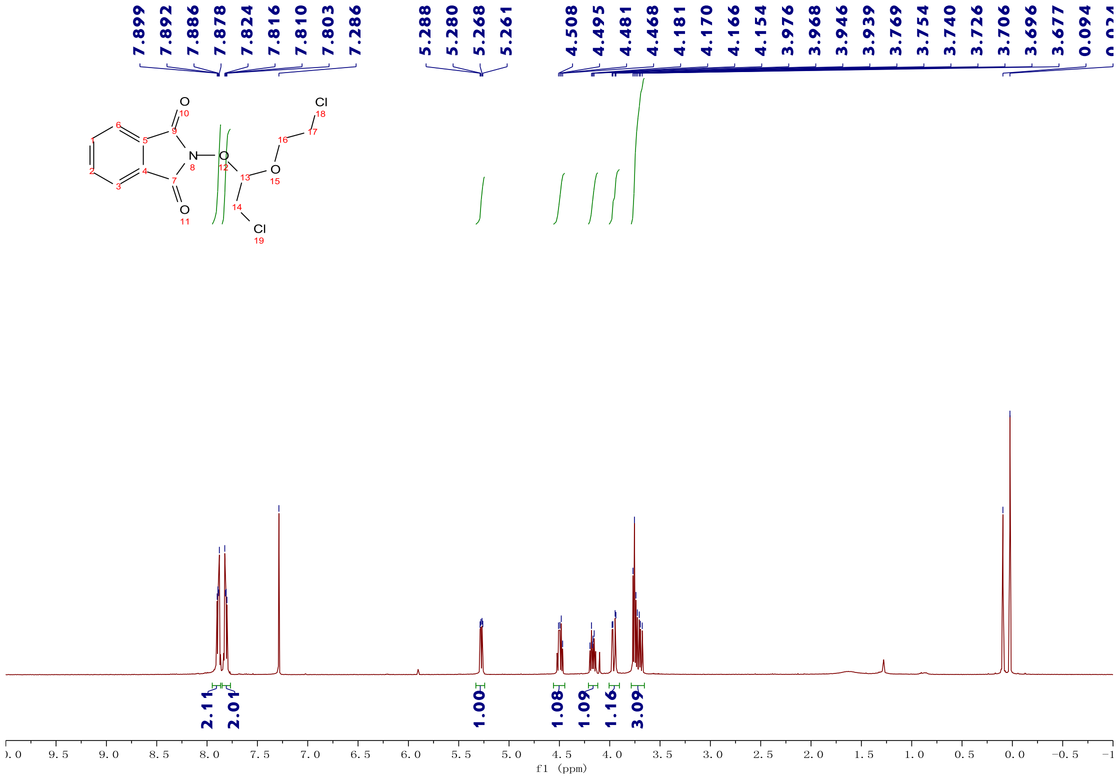

Supplementary Figure 49. ^{1}H NMR Spectra of **27**

27 (^{13}C, $CDCl_3$)

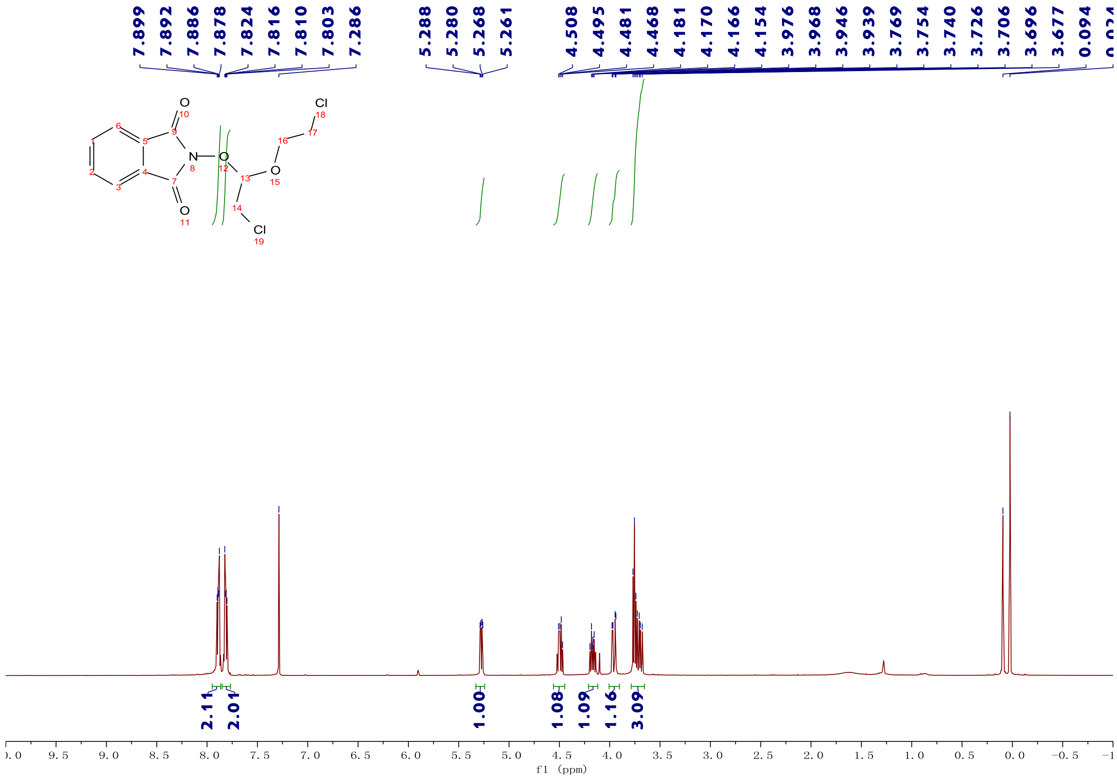

Supplementary Figure 50. ^{13}C NMR Spectra of **27**

28 (^{1}H, $CDCl_3$)

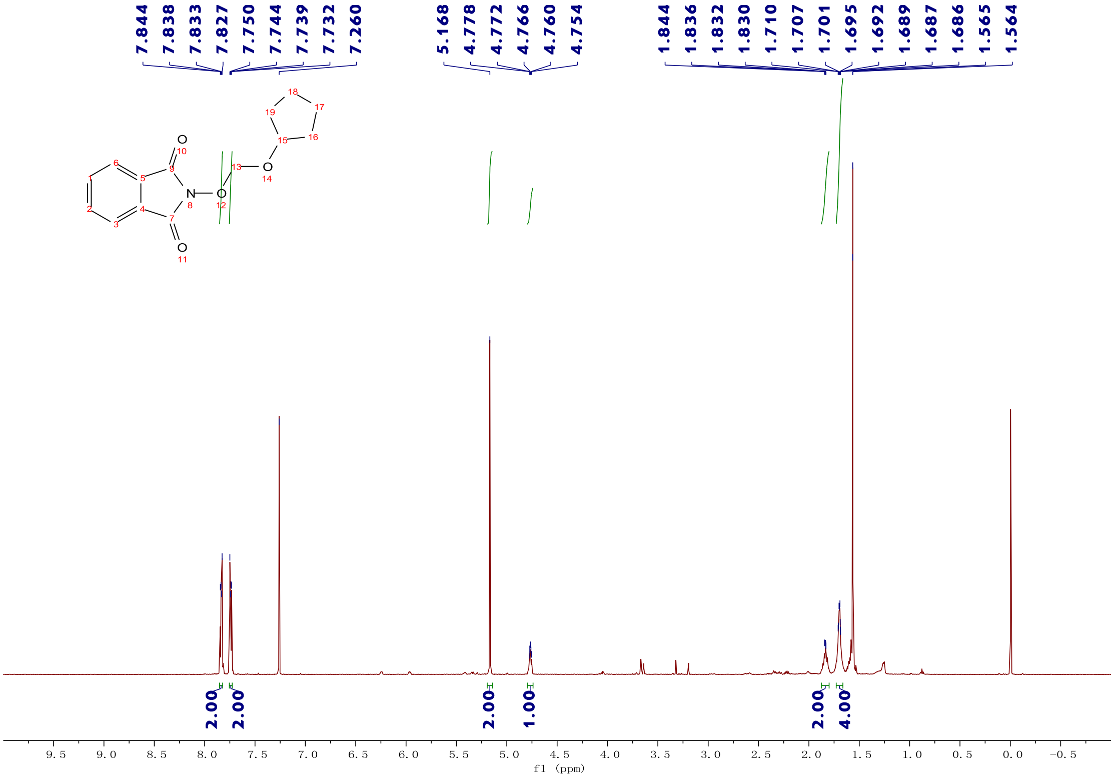

Supplementary Figure 51. ^{1}H NMR Spectra of **28**

28 (^{13}C, $CDCl_3$)

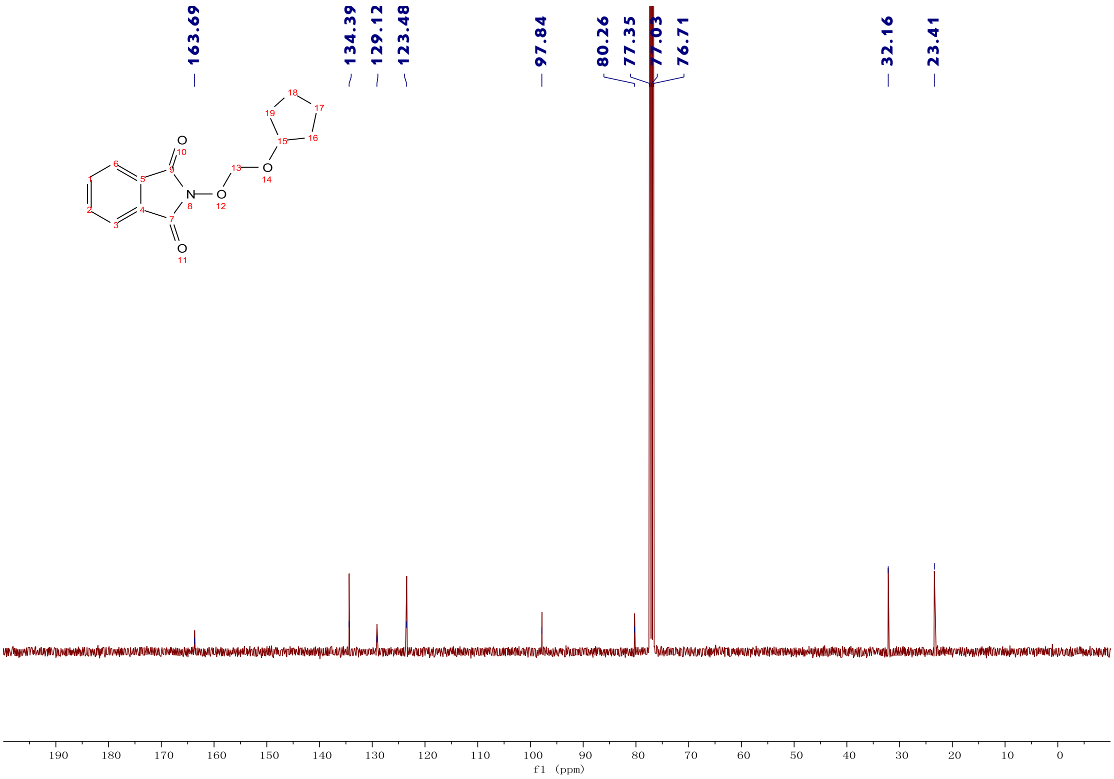

Supplementary Figure 52. ^{13}C NMR Spectra of **28**

29 (^{1}H, $CDCl_3$)

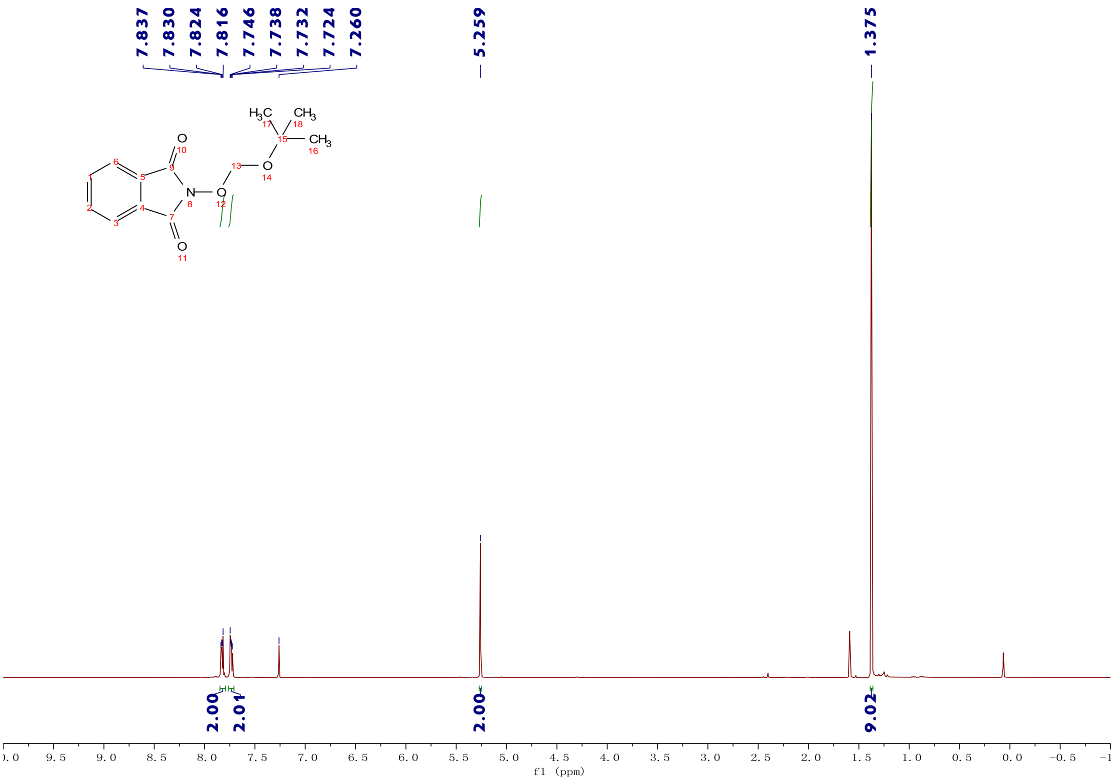

Supplementary Figure 53. ^{1}H NMR Spectra of **29**

29 (^{13}C, $CDCl_3$)

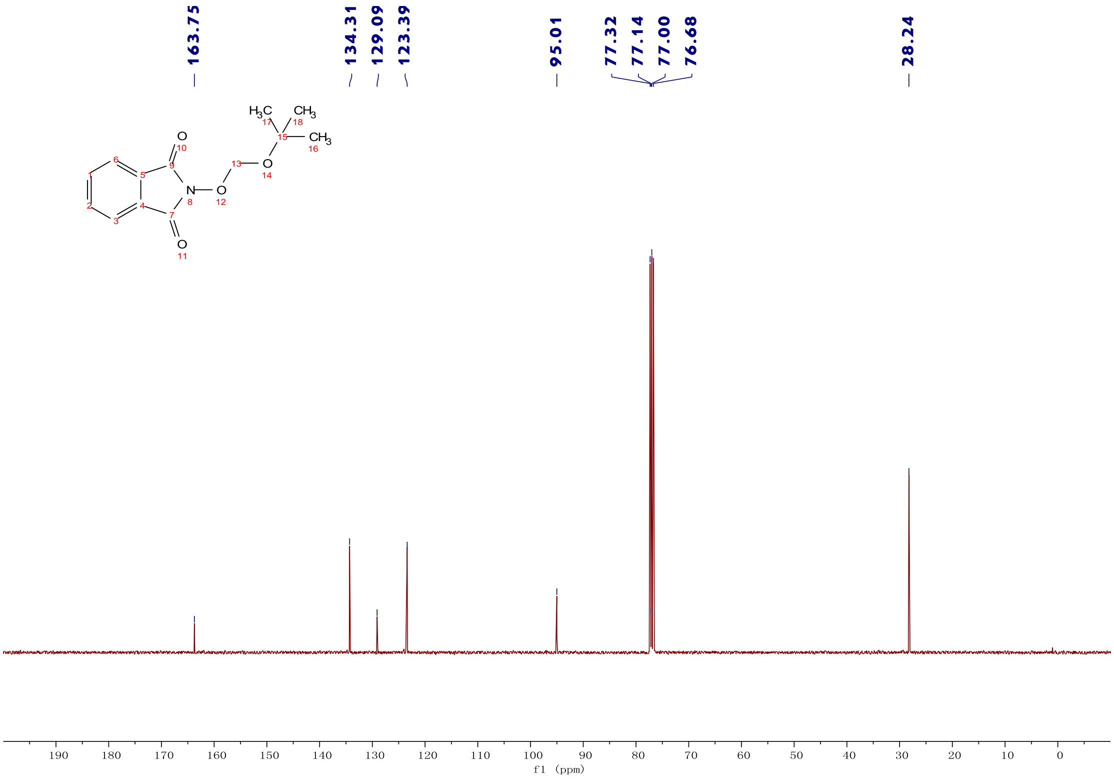

Supplementary Figure 54. ^{13}C NMR Spectra of **29**

30 (^{1}H, $CDCl_3$)

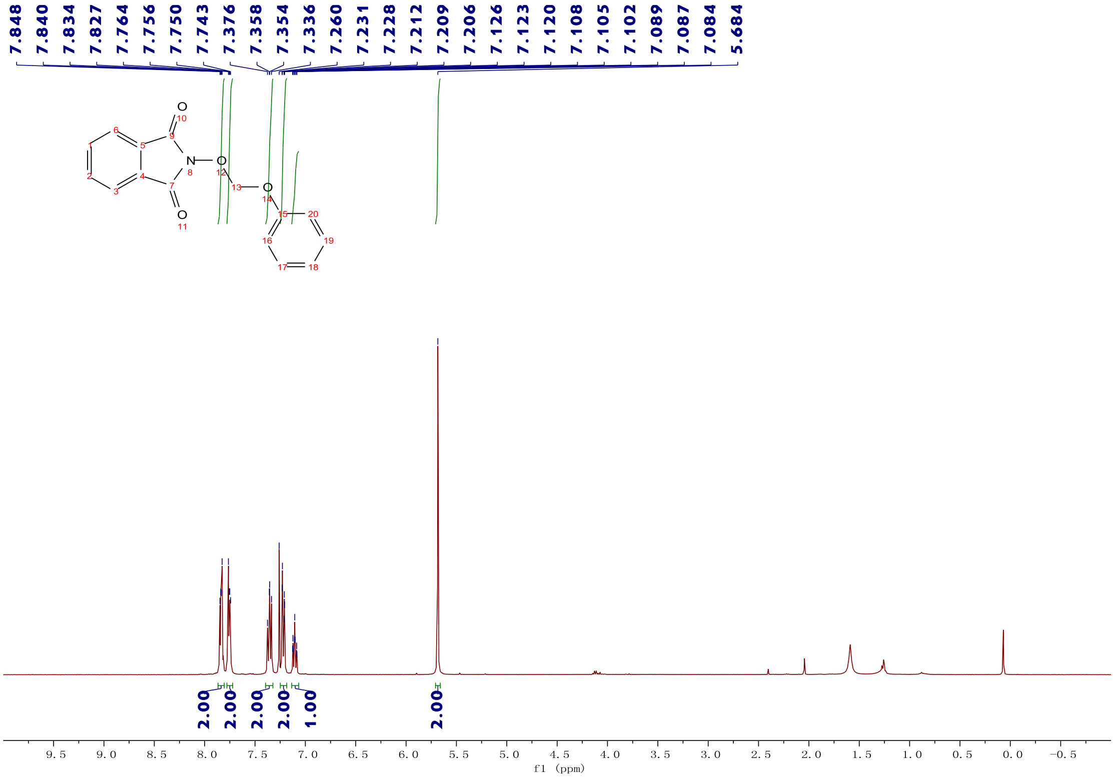

Supplementary Figure 55. ^{1}H NMR Spectra of **30**

30 (^{13}C, $CDCl_3$)

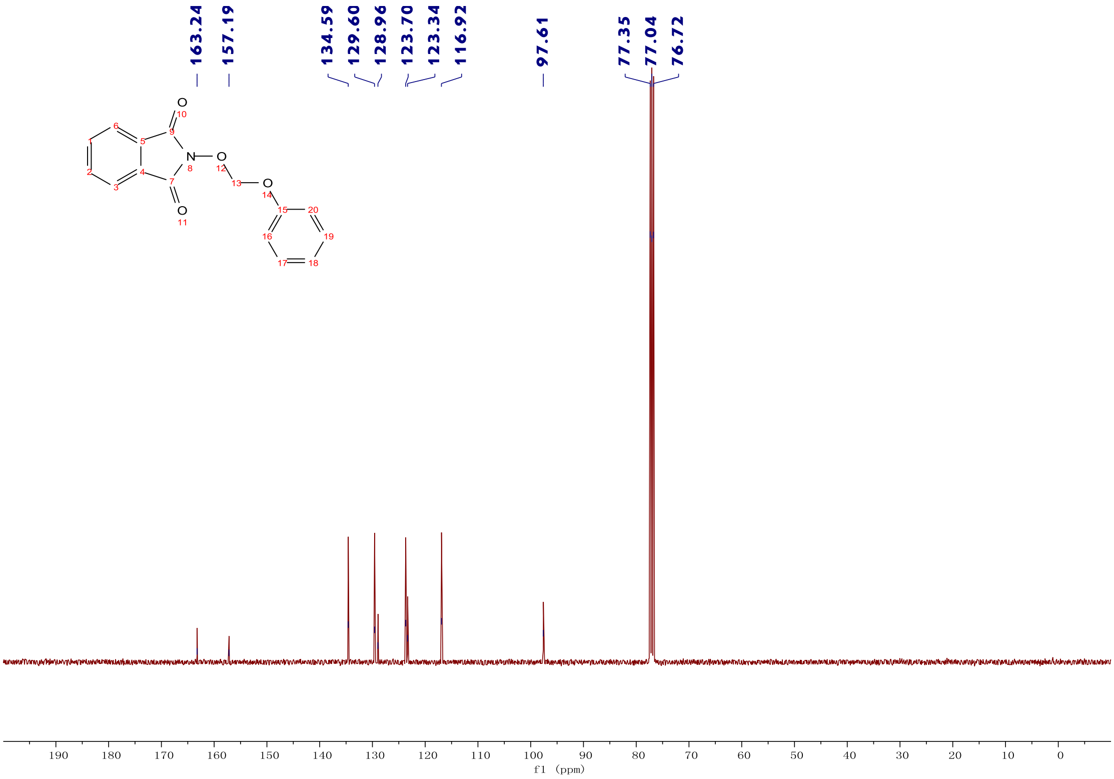

Supplementary Figure 56. ^{13}C NMR Spectra of **30**

31 (^{1}H, $CDCl_3$)

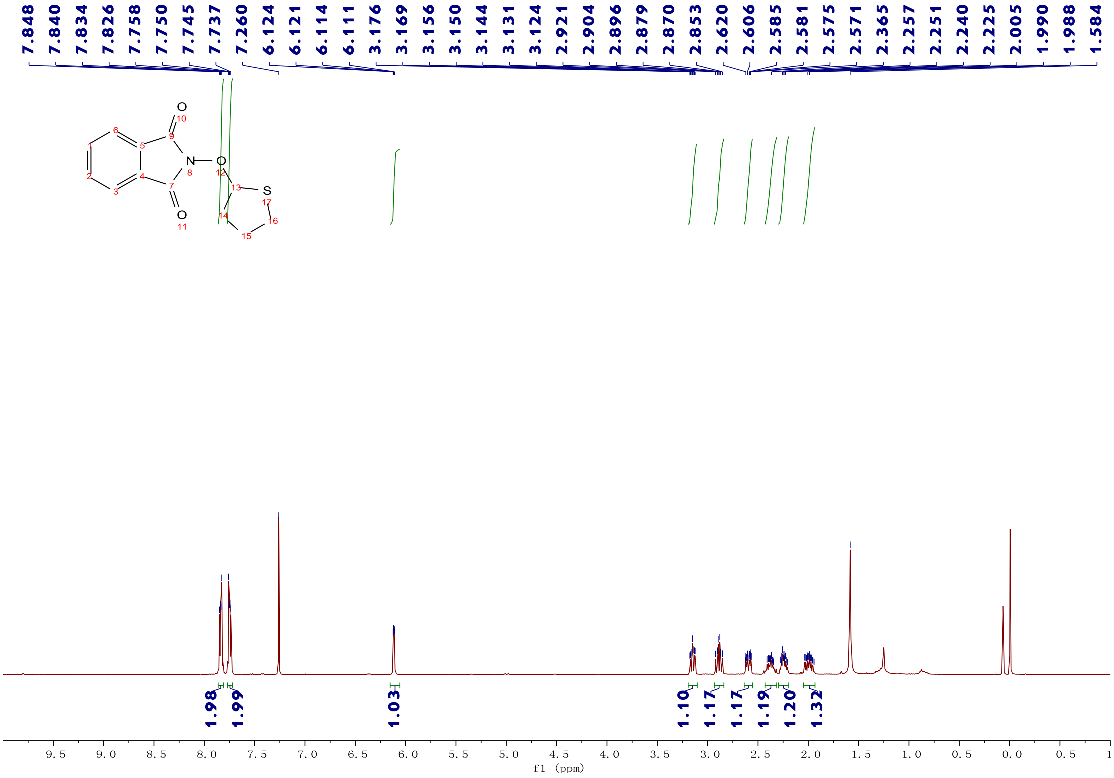

Supplementary Figure 57. ^{1}H NMR Spectra of **31**

31 (^{13}C, $CDCl_3$)

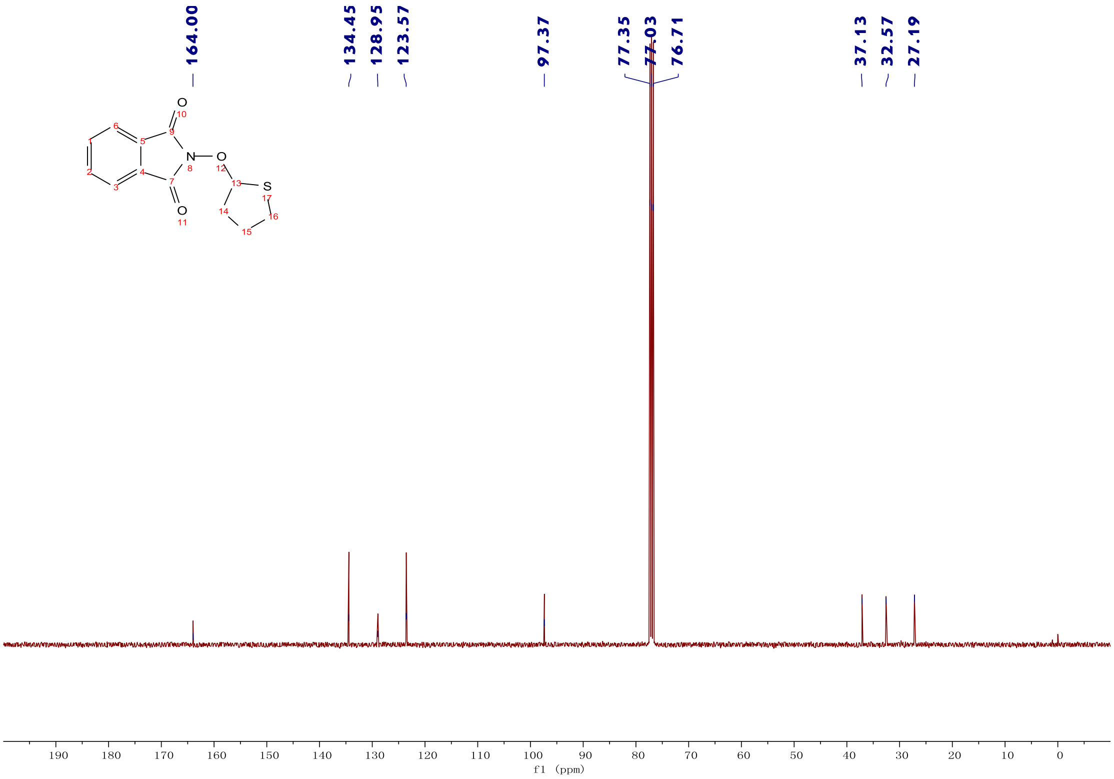

Supplementary Figure 58. ^{13}C NMR Spectra of **31**

32 (^{1}H, $CDCl_3$)

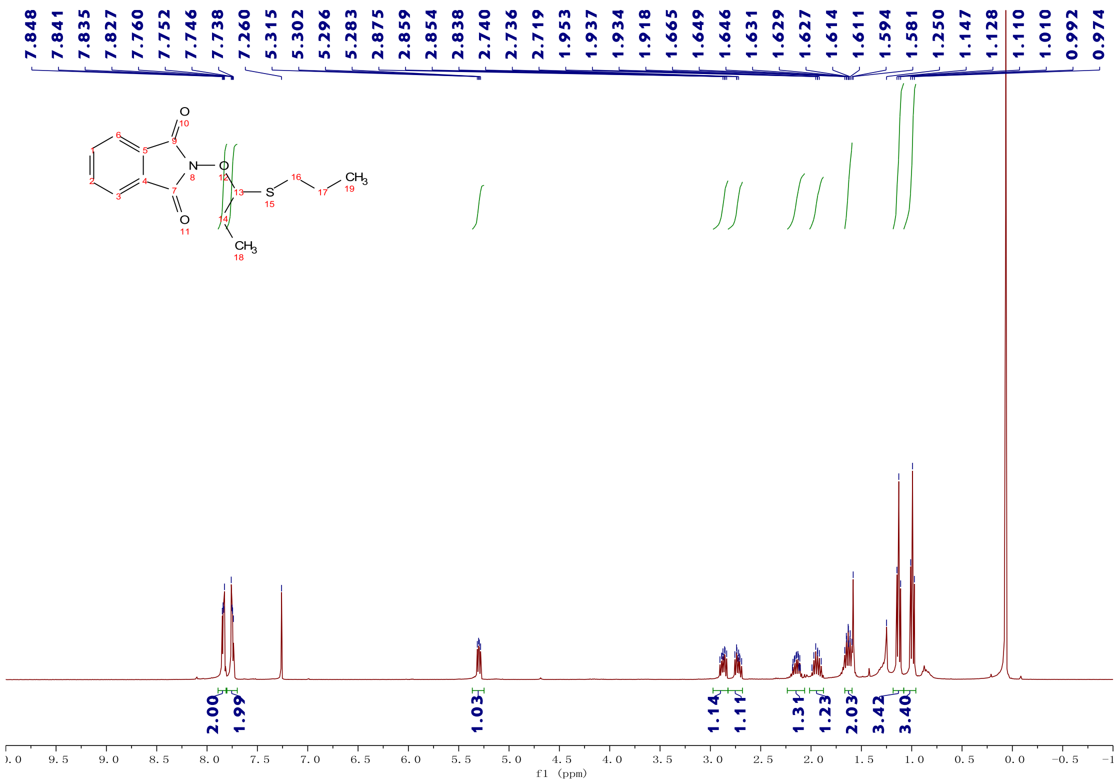

Supplementary Figure 59. ^{1}H NMR Spectra of **32**

32 (^{13}C, $CDCl_3$)

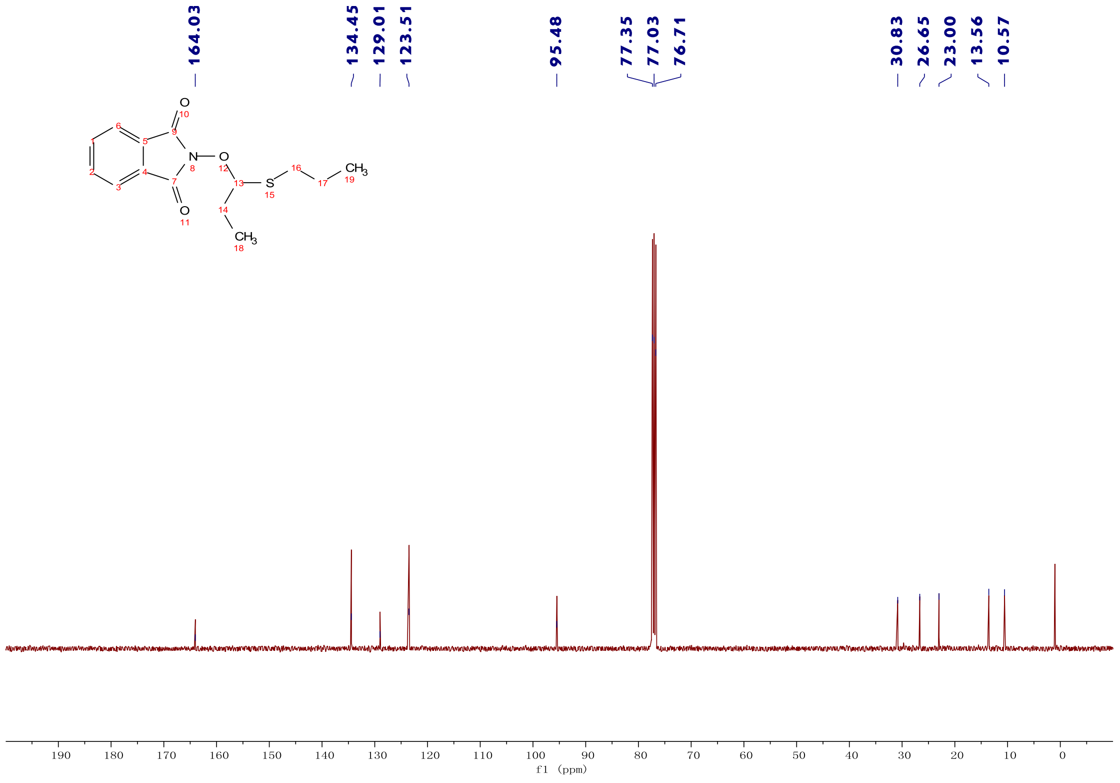

Supplementary Figure 60. ^{13}C NMR Spectra of **32**

33 (^{1}H, $CDCl_3$)

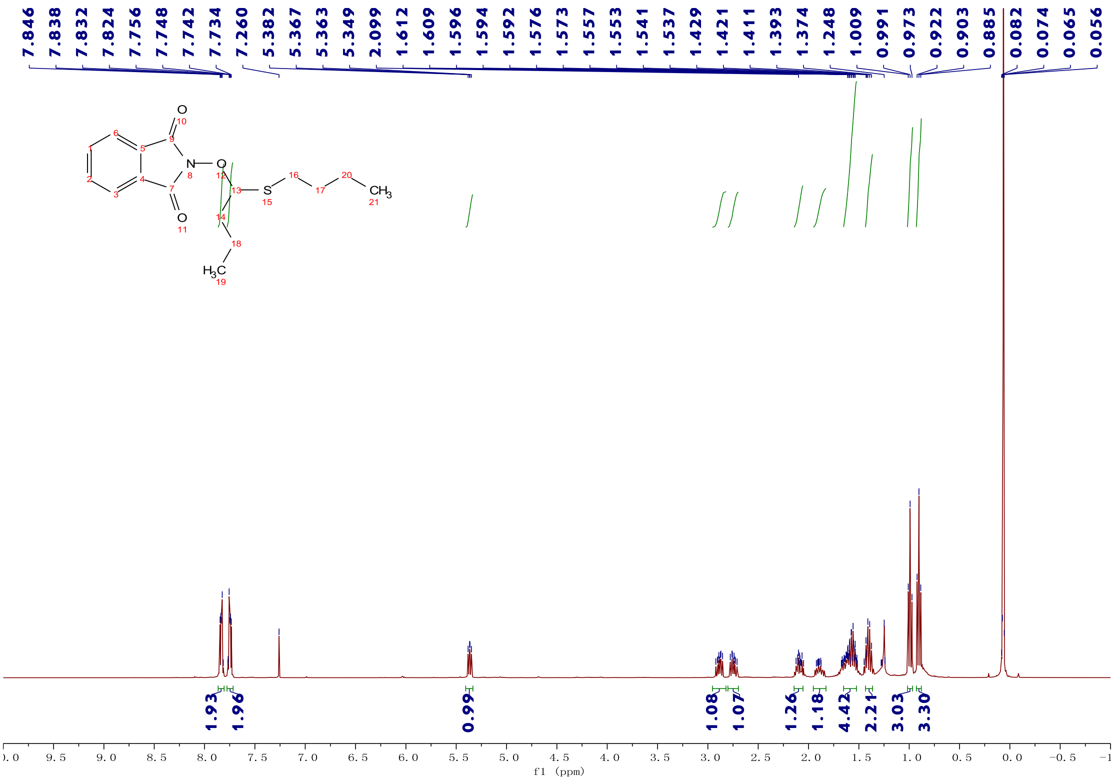

Supplementary Figure 61. ^{1}H NMR Spectra of **33**

33 (^{13}C, $CDCl_3$)

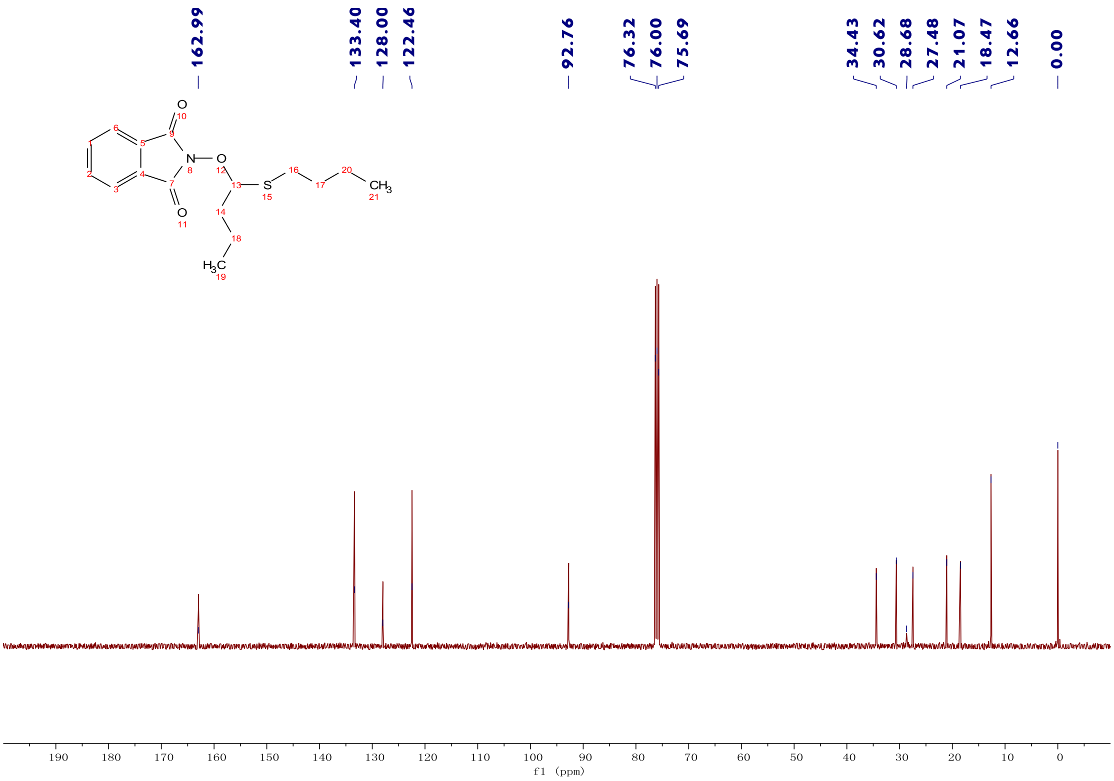

Supplementary Figure 62. ^{13}C NMR Spectra of **33**

34 (^{1}H, $CDCl_3$)

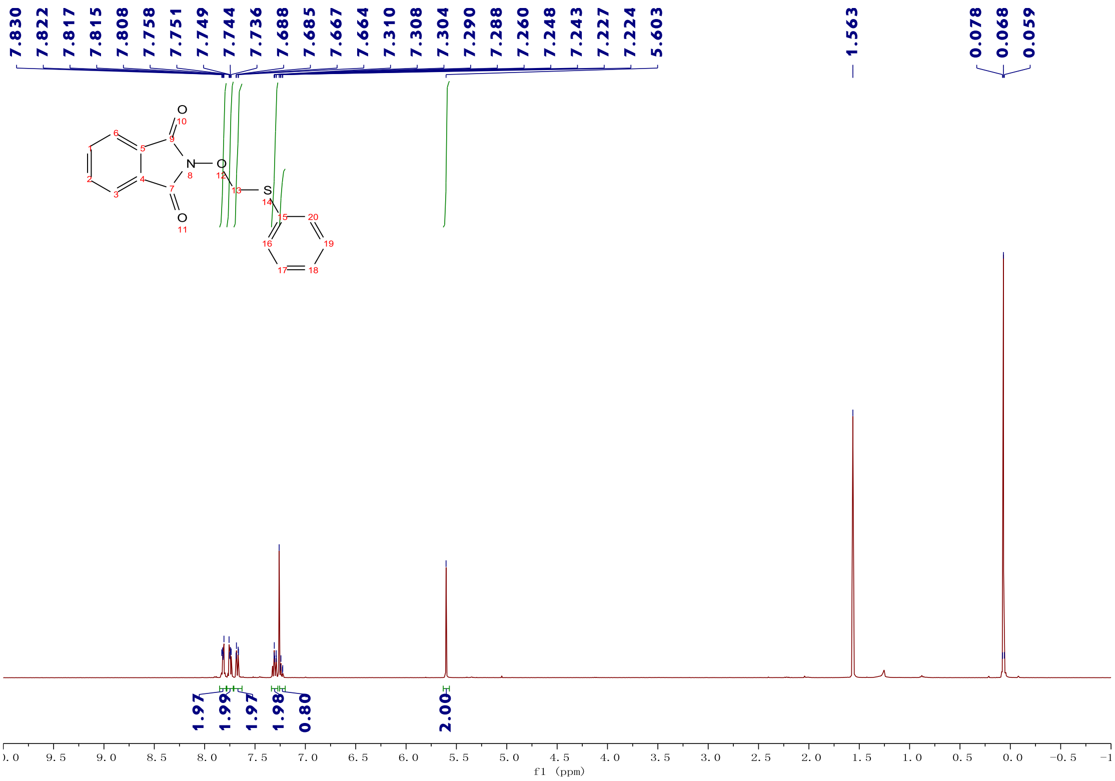

Supplementary Figure 63. ^{1}H NMR Spectra of **34**

34 (^{13}C, $CDCl_3$)

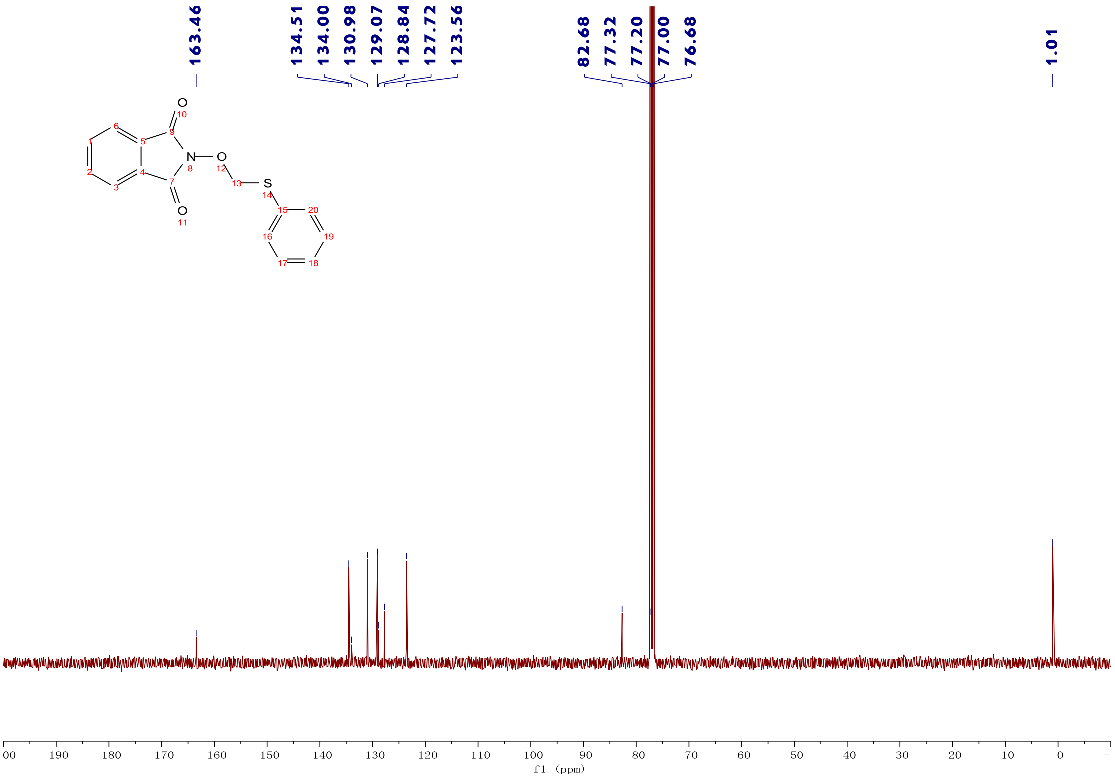

Supplementary Figure 64. ^{13}C NMR Spectra of **34**

35 (^{1}H, $CDCl_3$)

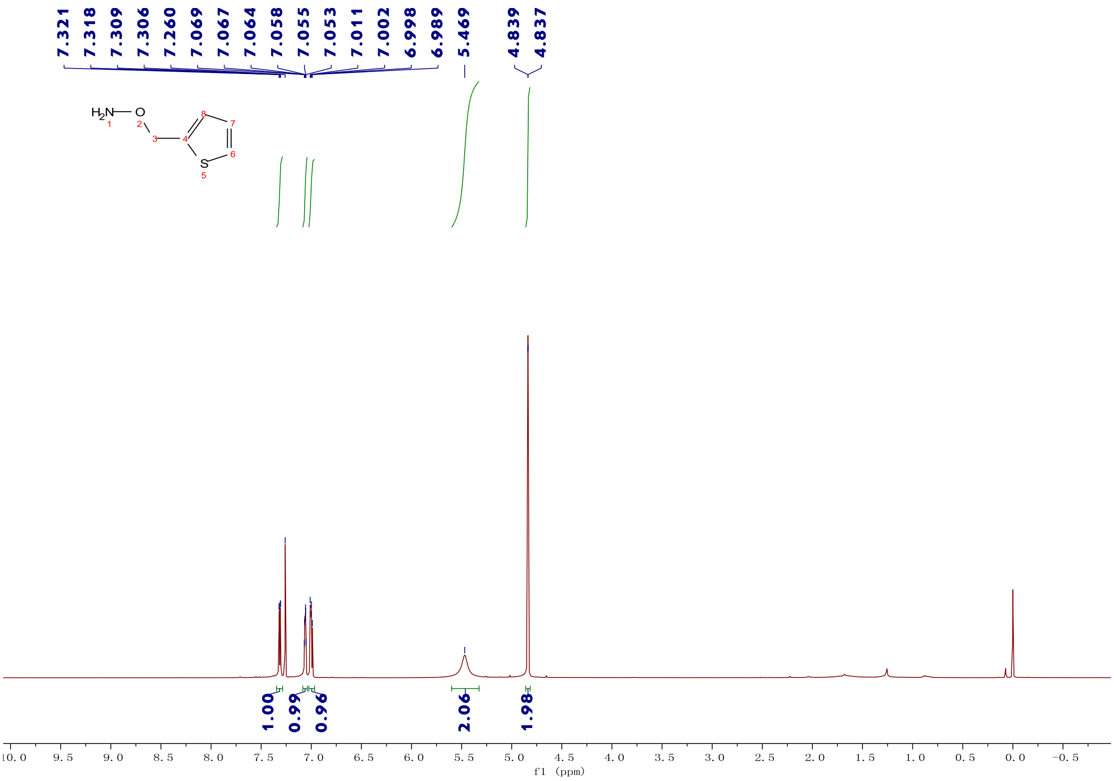

Supplementary Figure 65. ^{1}H NMR Spectra of **35**

35 (^{13}C, $CDCl_3$)

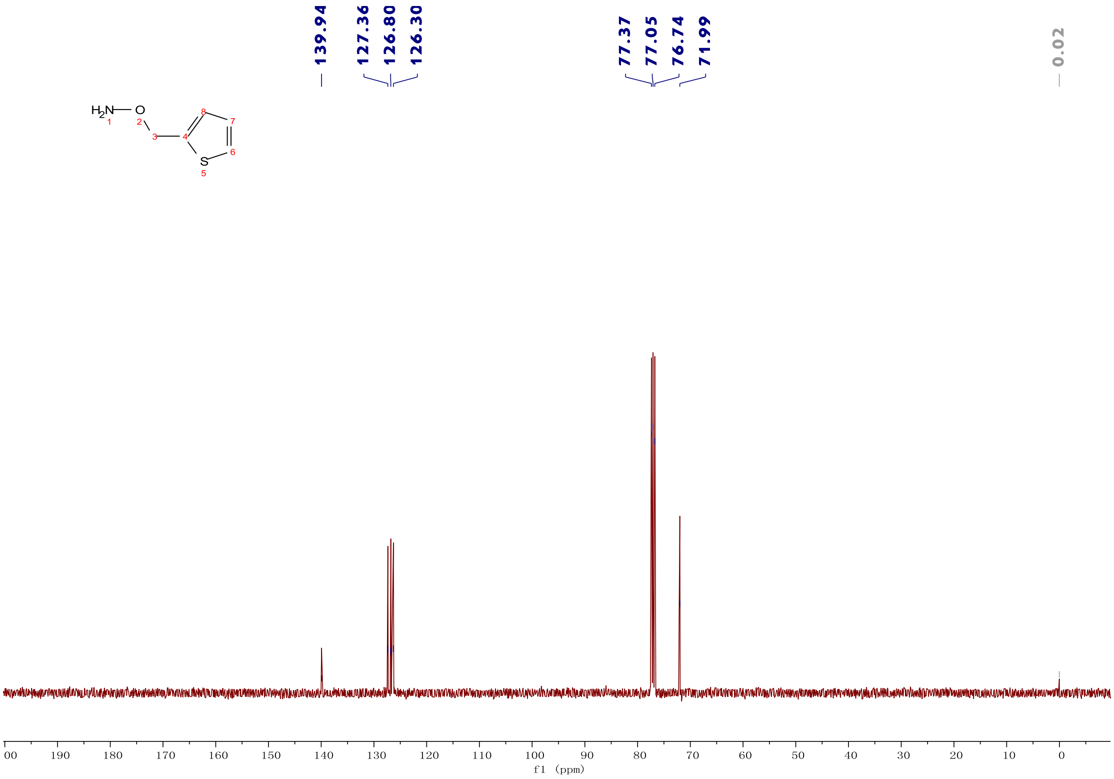

Supplementary Figure 66. ^{13}C NMR Spectra of **35**

Supplementary Note 1: Characterization data

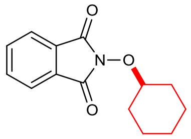

2-(Cyclohexyloxy)isoindoline-1,3-dione (3). White solid; 61% yield; m.p. 116–118 °C; ^{1}H NMR (400 MHz, $CDCl_3$) δ 7.84–7.82 (m, 2H), 7.75–7.73 (m, 2H), 4.26–4.19 (m, 1H), 2.06–2.00 (m, 2H), 1.90–1.83 (m, 2H), 1.63–1.54 (m, 3H), 1.25–1.28 (m, 3H). ^{13}C NMR (101 MHz, $CDCl_3$) δ 164.4, 134.4, 129.0, 123.5, 85.7, 30.8, 25.3, 23.8. ^{1}H NMR and ^{13}C NMR data correspond to the reported values.[1]

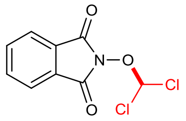

2-(Dichloromethoxy)isoindoline-1,3-dione (4). White solid; 50% yield; m.p.154–156 °C; ^{1}H NMR (400 MHz, $CDCl_3$) δ 7.92 (m, 2H), 7.83 (m, 2H), 7.43 (s, 1H). ^{13}C NMR (101 MHz, $CDCl_3$) δ 162.5, 135.2, 128.7, 124.3, 100.2. ^{1}H NMR and ^{13}C NMR data correspond to the reported values.[2]

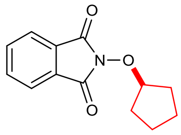

2-(Cyclopentyloxy)isoindoline-1,3-dione (5). White solid; 70% yield; ^{1}H NMR (400 MHz, $CDCl_3$) δ 7.84–7.82 (m, 2H), 7.75–7.73 (m, 2H), 4.94–4.91 (m, 1H), 2.02–1.92 (m, 4H), 1.80–1.74 (m, 2H), 1.64–1.58 (m, 2H). ^{13}C NMR (101 MHz, $CDCl_3$) δ 164.4, 134.4, 129.0, 123.5, 90.4, 31.5, 23.6. ^{1}H NMR and ^{13}C NMR data correspond to the reported values.[3]

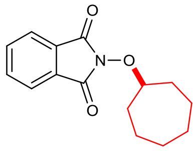

2-(Cycloheptyloxy)isoindoline-1,3-dione (6). White solid; 55% yield; m.p. 112–114 °C; ^{1}H NMR (400 MHz, $CDCl_3$) δ 7.84–7.82 (m, 2H), 7.75–7.73 (2H), 4.44–4.38 (m, 1H), 2.11–2.03 (m, 2H), 1.89–1.73 (m, 4H), 1.60–1.56 (m, 4H), 1.46–1.35 (m, 2H). ^{13}C NMR (101 MHz, $CDCl_3$) δ 163.4, 133.4, 128.0, 122.4, 87.7, 31.4, 27.4, 21.5. HRMS (ESI-TOF, m/z): $(M+H)^+$ calcd for $C_{15}H_{17}NO_3$, 261.1281; found, 261.1287.

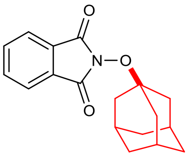

2-(((3s,5s,7s)-Adamantan-1-yl)oxy)isoindoline-1,3-dione (7). White solid; 85% yield; ^{1}H NMR (400 MHz, $CDCl_3$) δ 7.85–7.83 (m, 2H), 7.76–7.74 (m, 2H), 2.24–2.22 (m, 3H), 1.96 (d, J = 3.1 Hz, 6H), 1.64–1.57 (m, 6H). ^{13}C NMR (101 MHz, $CDCl_3$) δ 165.7, 134.4, 129.3, 123.4, 85.7, 41.1, 35.9, 31.0. ^{1}H NMR and ^{13}C NMR data correspond to the reported values.[4]

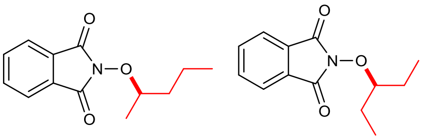

8a **8b**

inseperable 76% (**8a**:**8b** =1.7:1)

2-**(Pentan-2-yloxy)isoindoline-1,3-dione (8a):2-(Pentan-3-yloxy)isoindoline-1,3-dione (8b):** Yellow oil; 80% yield; ^{1}H NMR (400 MHz, $CDCl_3$) δ 7.84–7.81 (m, 2H), 7.75–7.72 (m, 2H), 4.40–4.36 (m, 0.57H), 4.15–4.12 (m, 0.33H), 1.83–1.68 (m, 2H), 1.63–1.47 (m, 2H), 1.33 (d, J = 6.3 Hz,1.71H), 1.04 (t, J = 7.4 Hz, 1.98H), 0.96 (t, J = 7.4 Hz,1.71H). ^{13}C NMR (101 MHz, $CDCl_3$) δ 164.4, 134.4, 134.4, 129.1, 129.0, 123.4, 123.4, 90.4, 84.3, 37.0, 24.7, 18.8, 18.6, 14.0, 9.2. HRMS (ESI-TOF, m/z): $(M+H)^+$ calcd for $C_{13}H_{15}NO_3$, 234.1125; found, 234.1128.

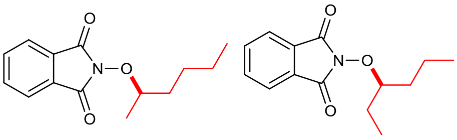

9a **9b**

inseperable 66% (**9a**:**9b** =1:2.4)

2-(Hexan-2-yloxy)isoindoline-1,3-dione (9a):2-(Hexan-3-yloxy)isoindoline-1,3-dione (9b): Yellow oil; 67% yield; ^{1}H NMR (400 MHz, $CDCl_3$) δ 7.84–7.82 (m, 2H), 7.75–7.73 (m, 2H), 4.41–4.34 (m, 0.32H), 4.23–4.17 (m, 0.77H), 1.81–1.63 (m, 4H), 1.56–1.45 (m, 2H), 1.33 (d, J = 6.4 Hz,0.96H), 1.04 (t, J = 7.2 Hz, 2.31H), 0.96–0.92 (m,3.29H); ^{13}C NMR (101 MHz, $CDCl_3$) δ 164.4, 134.4, 134.4, 129.1, 129.0, 123.4, 123.4, 89.2, 84.5, 34.6, 34.0, 27.4, 25.3, 22.7, 18.8, 18.4, 14.2, 14.0, 9.1. HRMS (ESI-TOF, m/z): $(M+H)^+$ calcd for $C_{14}H_{17}NO_3$, 248.1281; found, 248.1286.

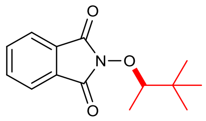

(R)-2-((3,3-Dimethylbutan-2-yl)oxy)isoindoline-1,3-dione (10). Yellow solid; 48% yield; m.p. 98–101 °C; ^{1}H NMR (400 MHz, $CDCl_3$) δ 7.83-7.82 (m, 2H), 7.74–7.72 (m, 2H), 4.09 (q, J = 6.4 Hz, 1H), 1.23 (d, J = 6.4 Hz, 3H), 1.09 (s, 9H). ^{13}C NMR (101 MHz, $CDCl_3$) δ 163.4, 133.3, 128.0, 122.3, 90.0, 33.7, 25.0, 12.8. HRMS (ESI-TOF, m/z): $(M+H)^+$ calcd for $C_{14}H_{17}NO_3$, 248.1281; found, 248.1275.

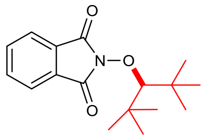

2-((2,2,4,4-Tetramethylpentan-3-yl)oxy)isoindoline-1,3-dione (11). White solid; 88% yield; m.p. 109–111 °C; ^{1}H NMR (400 MHz, $CDCl_3$) δ 7.80–7.76 (m, 2H), 7.72-7.71 (m, 2H), 4.34 (s, 1H), 1.18 (s, 18H). ^{13}C NMR (101 MHz, $CDCl_3$) δ 164.2, 134.3, 129.1, 123.1, 98.6, 38.2, 29.4. HRMS (ESI-TOF, m/z): $(M+Na)^+$ calcd for $C_{17}H_{23}NO_3$, 312.1570; found, 312.1578.

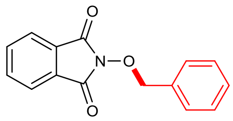

2-(Benzyloxy)isoindoline-1,3-dione (12). White solid; 88% yield; ^{1}H NMR (400 MHz, $CDCl_3$) δ 7.82–7.80 (m, 2H), 7.74–7.72 (m, 2H), 7.55–7.53 (m, 2H), 7.39–7.37 (m, 3H),

5.21 (s, 2H). ^{13}C NMR (101 MHz, $CDCl_3$) δ 163.5, 134.4, 133.7, 130.0, 129.4, 128.9, 128.6, 123.5, 80.0. ^{1}H NMR and ^{13}C NMR data correspond to the reported values.[5]

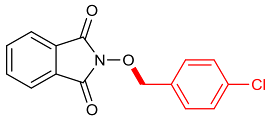

2-((4-Chlorobenzyl)oxy)isoindoline-1,3-dione (13). White solid; 75% yield; ^{1}H NMR (400 MHz, $CDCl_3$) δ 7.82–7.80 (m, 2H), 7.75–7.73 (m, 2H), 7.49–7.49 (m, 2H), 7.36–7.34 (m, 2H), 5.18 (s, 2H). ^{13}C NMR (101 MHz, $CDCl_3$) δ 163.5, 135.4, 134.5, 132.2, 131.2, 128.8, 124.1, 123.6, 79.0. ^{1}H NMR and ^{13}C NMR data correspond to the reported values.[5]

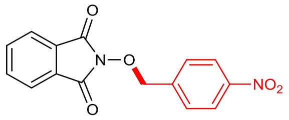

2-((4-Nitrobenzyl)oxy)isoindoline-1,3-dione (14). White solid; 94% yield; m.p. 191–193 ^{o}C; ^{1}H NMR (400 MHz, $CDCl_3$) δ 8.25 (d, J = 8.8 Hz, 2H), 7.84–7.83 (m, 2H), 7.80–7.73 (m, 4H), 5.32 (s, 2H). ^{13}C NMR (101 MHz, $CDCl_3$) δ 163.4, 148.4, 140.9, 134.7, 130.1, 128.7, 123.8, 123.7, 78.3. ^{1}H NMR and ^{13}C NMR data correspond to the reported values.[6]

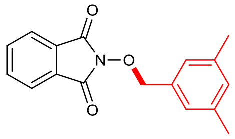

2-((3,5-Dimethylbenzyl)oxy)isoindoline-1,3-dione (15). White solid; 75% yield; ^{1}H NMR (400 MHz, $CDCl_3$) δ 7.83–7.81 (m, 2H), 7.75–7.73 (m, 2H), 7.16 (s, 2H), 7.01 (s, 1H), 5.13 (s, 2H), 2.32 (s, 6H). ^{13}C NMR (101 MHz, $CDCl_3$) δ 163.5, 138.2, 134.4, 133.4, 131.0, 129.0, 127.6, 123.50, 80.1, 21.2. ^{1}H NMR and ^{13}C NMR data correspond to the reported values.[7]

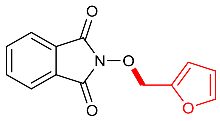

2-(Furan-2-ylmethoxy)isoindoline-1,3-dione (16). Yellow solid; 56% yield; m.p. 140–142 °C; ^{1}H NMR (400 MHz, $CDCl_3$) δ 7.82–7.80 (m, 2H), 7.74–7.73 (m, 2H), 7.48 (dd, J = 1.9, 0.8 Hz, 1H), 6.49 (dd, J = 3.3, 0.8 Hz, 1H), 6.34 (dd, J = 3.3, 1.8 Hz, 1H), 5.16 (s, 2H); ^{13}C NMR (101 MHz, $CDCl_3$) δ 163.3, 148.0, 144.5, 134.4, 128.8, 123.5, 113.3, 110.8, 70.3. HRMS (ESI-TOF, m/z): $(M+Na)^+$ calcd for $C_{13}H_9NO_4$, 266.0424; found, 266.0418.

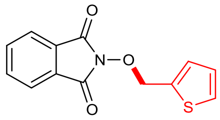

2-(Thiophen-2-ylmethoxy)isoindoline-1,3-dione (17). Yellow solid; 93% yield; m.p. 118–120 °C; ^{1}H NMR (400 MHz, $CDCl_3$) δ 7.82–7.80 (m, 2H), 7.75–7.73 (m, 2H), 7.40 (dd, J = 5.1, 1.2 Hz, 1H), 7.19 (dd, J = 3.5, 1.1 Hz, 1H), 6.99 (dd, J = 5.1, 3.5 Hz, 1H), 5.37 (s, 2H); ^{13}C NMR (101 MHz, $CDCl_3$) δ 163.4, 135.3, 134.5, 130.4, 128.8, 128.6, 127.1, 123.6, 73.0. HRMS (ESI-TOF, m/z): $(M+Na)^+$ calcd for $C_{13}H_9NO_3S$, 282.0195; found, 282.0202.

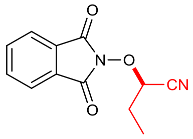

2-((1,3-Dioxoisoindolin-2-yl)oxy)butanenitrile (18). White solid; 78% yield; m.p. 146–148°C; ^{1}H NMR (400 MHz, $CDCl_3$) δ 7.90–7.88 (m, 2H), 7.82–7.79 (m, 2H), 5.02 (t, J = 6.6 Hz, 1H), 2.18–2.14 (m, 2H), 1.26 (t, J = 7.5 Hz, 1H). ^{13}C NMR (101 MHz, $CDCl_3$) δ 162.9, 135.0, 134.4, 128.6, 124.1, 123.6, 116.2, 76.4, 25.3, 9.0. HRMS (ESI-TOF, m/z): $(M+H)^+$ calcd for $C_{12}H_{10}N_2O_3$, 231.0764; found, 231.0764.

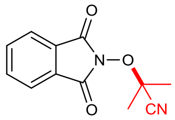

2-((1,3-Dioxoisoindolin-2-yl)oxy)-2-methylpropanenitrile (19). White solid. 86% yield; m.p. 148–150 °C; ^{1}H NMR (400 MHz, $CDCl_3$) δ 7.92–7.90 (m, 2H), 7.82–7.80 (m, 2H), 1.85 (s, 6H). ^{13}C NMR (101 MHz, $CDCl_3$) δ 164.2, 135.0, 129.0, 124.1, 119.3,

79.3, 26.0. HRMS (ESI-TOF, m/z): $(M+Na)^+$ calcd for $C_{12}H_{10}N_2O_3$, 253.0584; found, 253.0581.

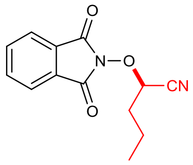

2-((1,3-Dioxoisoindolin-2-yl)oxy)pentanenitrile (20). Yellow oil; 45% yield; ^1H NMR (400 MHz, $CDCl_3$) δ 7.86–7.84 (m, 2H), 7.78–7.76 (m, 2H), 4.44–4.36 (m, 1H), 2.90–2.82 (m, 1H), 2.77–2.58 (m, 1H), 2.13–1.96 (m, 2H), 1.42 (d, J = 6.4 Hz, 3H). ^{13}C NMR (101 MHz, $CDCl_3$) δ 164.2, 134.7, 128.8, 123.7, 119.6, 82.8, 29.7, 19.0, 13.5. HRMS (ESI-TOF, m/z): $(M+Na)^+$ calcd for $C_{13}H_{12}N_2O_3$, 267.0740; found, 267.0742.

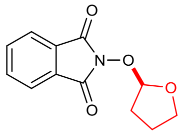

2-((Tetrahydrofuran-2-yl)oxy)isoindoline-1,3-dione (21). White solid; 62% yield; m.p. 132-133 ℃; ^1H NMR (400 MHz, $CDCl_3$) δ 7.84–7.81 (m, 2H), 7.74–7.72 (m, 2H), 5.78 (d, J = 4.8 Hz, 1H), 4.38–4.34 (m, 1H), 4.07–4.00 (m, 1H), 2.34–2.20 (m, 2H), 2.15–2.07 (m, 1H), 1.99–1.90 (m, 1H). ^{13}C NMR (101 MHz, $CDCl_3$) δ 163.9, 134.3, 129.1, 123.4, 108.8, 69.2, 30.8, 22.6. ^1H NMR and ^{13}C NMR data correspond to the reported values.[5]

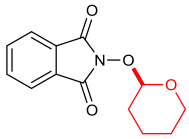

2-((Tetrahydro-2H-pyran-2-yl)oxy)isoindoline-1,3-dione (22). White solid; 86% yield; m.p. 123–124 ℃; ^1H NMR (400 MHz, $CDCl_3$) δ 7.84–7.82 (m, 2H), 7.74–7.72 (m, 2H), 5.42–5.41 (m, 1H), 4.51 (td, J = 11.1, 3.8 Hz, 1H), 3.68–3.64 (m, 1H), 2.13–2.09 (m, 1H), 1.94–1.88 (m, 1H), 1.87–1.82 (m, 1H), 1.74–1.66 (m, 3H). ^{13}C NMR

(101 MHz, $CDCl_3$) δ 163.8, 134.2, 129.2, 123.4, 103.1, 62.3, 27.7, 24.8, 17.6. ^{1}H NMR and ^{13}C NMR data correspond to the reported values.[7]

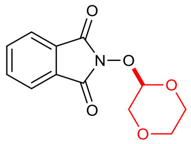

2-((1,4-Dioxan-2-yl)oxy)isoindoline-1,3-dione (23). White solid; 89% yield; m.p. 185–187 ℃; ^{1}H NMR (400 MHz, $CDCl_3$) δ 7.86–7.84 (m, 2H), 7.76–7.74 (m, 2H), 5.24 (d, J = 2.4 Hz, 1H), 4.85 (dt, J = 11.7, 3.2 Hz, 1H), 4.16 (d, J = 12.8 Hz, 1H), 3.92–3.88 (m, 1H), 3.83 (d, J = 2.4 Hz, 1H), 3.81–3.80 (m, 1H), 3.55 (dd, J = 11.9, 2.1 Hz, 1H). ^{13}C NMR (101 MHz, $CDCl_3$) δ 163.6, 134.5, 129.0, 123.6, 99.5, 66.2, 66.0, 60.9. ^{1}H NMR and ^{13}C NMR data correspond to the reported values.[1]

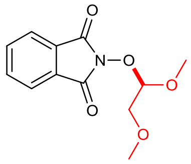

2-(1,2-Dimethoxyethoxy)isoindoline-1,3-dione (24). White solid. 83% yield; ^{1}H NMR (400 MHz, $CDCl_3$) δ 7.86–7.84 (m, 2H), 7.77–7.75 (m, 2H), 5.14 (dd, J = 6.2, 3.8 Hz, 1H), 3.82–3.78 (m, 1H), 3.77 (s, 3H), 3.61 (dd, J = 10.7, 6.2 Hz, 1H), 3.42 (s, 3H). ^{13}C NMR (101 MHz, $CDCl_3$) δ 164.0, 134.5, 129.0, 123.6, 108.3, 71.7, 59.5, 57.1. ^{1}H NMR and ^{13}C NMR data correspond to the reported values.[8]

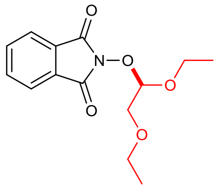

2-(1,2-Diethoxyethoxy)isoindoline-1,3-dione(25). White solid; 67% yield; m.p. 120–122 °C; ^{1}HNMR (400 MHz, $CDCl_3$) δ 7.85–7.83 (m, 2H), 7.77–7.74 (m, 2H), 4.26–4.18 (m, 1H), 3.95–3.91 (m, 1H), 3.86 (dd, J = 10.9, 4.1 Hz, 1H), 3.63 (dd, J = 10.9, 4.1 Hz, 1H), 3.59–3.54 (m, 2H), 1.2 (t, J = 7.1 Hz, 3H), 1.17 (t, J = 7.1 Hz, 3H). ^{13}C

NMR (101 MHz, $CDCl_3$) δ 164.1, 134.5, 129.1, 123.5, 107.4, 70.2, 67.2, 65.9, 65.4, 15.0. HRMS (ESI-TOF, m/z): $(M+Na)^+$ calcd for $C_{14}H_{17}NO_5$, 302.0999; found, 302.0996.

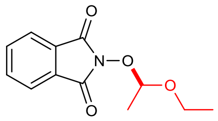

2-(1-Ethoxyethoxy)isoindoline-1,3-dione (26). Yellow oil; 78% yield; ^{1}H NMR (400 MHz, $CDCl_3$) δ 7.85–7.83 (m, 2H), 7.76–7.74 (m, 2H), 5.30 (q, J = 5.4 Hz, 1H), 4.21–4.13 (m, 1H), 3.86–3.78 (m, 1H), 1.53 (d, J = 5.4 Hz, 3H), 1.24 (t, J = 6.8 Hz, 3H). ^{13}C NMR (101 MHz, $CDCl_3$) δ 164.4, 134.4, 129.2, 123.5, 106.4, 63.6, 18.9, 15.0. ^{1}H NMR and ^{13}C NMR data correspond to the reported values.[7]

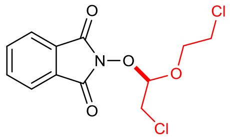

2-(2-Chloro-1-(2-chloroethoxy)ethoxy)isoindoline-1,3-dione (27). White solid; 92% yield; ^{1}H NMR (400 MHz, $CDCl_3$) δ 7.87–7.85 (m, 2H), 7.80–7.78 (m, 2H), 5.24 (dd, J = 7.7, 3.1 Hz, 1H), 4.49–4.39 (m, 1H), 4.17–4.07 (m, 1H), 3.93 (dd, J = 11.9, 3.1 Hz, 1H), 3.74–3.65 (m, 3H). ^{13}C NMR (101 MHz, $CDCl_3$) δ 164.0, 134.8, 128.9, 123.8, 108.0, 70.00, 42.5, 42.4. ^{1}H NMR and ^{13}C NMR data correspond to the reported values.[8]

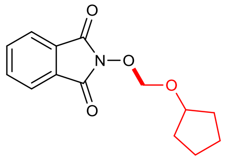

2-((Cyclopentyloxy)methoxy)isoindoline-1,3-dione (28). White solid; 57% yield; ^{1}H NMR (400 MHz, $CDCl_3$) δ 7.85–7.83 (m, 2H), 7.75–7.32 (m, 2H), 5.17 (s, 2H), 4.79–4.75 (m, 1H), 1.88–1.81 (m, 2H), 1.74–1.67 (m, 4H), 1.61–1.58 (m, 2H). ^{13}C NMR (101 MHz, $CDCl_3$) δ 163.7, 134.4, 129.1, 123.5, 97.8, 80.3, 32.2, 23.4. ^{1}H NMR and ^{13}C NMR data correspond to the reported values.[1]

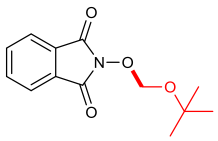

2-(Tert-butoxymethoxy)isoindoline-1,3-dione (29). White solid; 64% yield; ^{1}H NMR (400 MHz, $CDCl_3$) δ 7.84–7.82 (m, 2H), 7.75–7.72 (m, 2H), 5.26 (s, 2H), 1.38 (s, 9H). ^{13}C NMR (101 MHz, $CDCl_3$) δ 163.8, 134.3, 129.1, 123.4, 95.0, 77.1, 28.24. ^{1}H NMR and ^{13}C NMR data correspond to the reported values.[1]

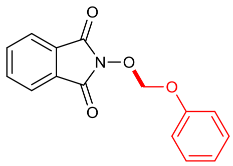

2-(Phenoxymethoxy)isoindoline-1,3-dione (30). Yellow solid; 77% yield; m.p. 100–102 °C; ^{1}H NMR (400 MHz, $CDCl_3$) δ 7.85–7.83 (m, 2H), 7.76–7.74 (m, 2H), 7.38–7.34 (m, 2H), 7.23–7.21 (m, 2H), 7.13–7.08 (m, 1H), 5.68 (s, 2H). ^{13}C NMR (101 MHz, $CDCl_3$) δ 163.2, 157.2, 134.6, 129.6, 129.0, 123.7, 123.3, 116.9, 97.6. HRMS (ESI-TOF, m/z): calcd for $C_{15}H_{11}NO_4$ $(M+H)^+$, 270.0761, found 270.0756. ^{1}H NMR and ^{13}C NMR datas correspond to the reported values.[9]

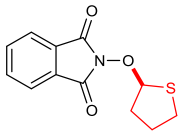

(S)-2-((Tetrahydrothiophen-2-yl)oxy)isoindoline-1,3-dione (31). Yellow solid; 65% yield; ^{1}H NMR (400 MHz, $CDCl_3$) δ 7.85–7.83 (m, 2H), 7.76–7.74 (m, 2H), 6.12 (dd, J = 4.0, 1.2 Hz, 1H), 3.18–3.12 (m, 1H), 2.92–2.85 (m, 1H), 2.62–2.57 (m, 1H), 2.41–2.34 (m, 1H), 2.27–2.21 (m, 1H), 2.04–1.94 (m, 1H). ^{13}C NMR (101 MHz, $CDCl_3$) δ 164.0, 134.4, 129.0, 123.6, 97.4, 37.1, 32.6, 27.2. ^{1}H NMR and ^{13}C NMR data correspond to the reported values.[7]

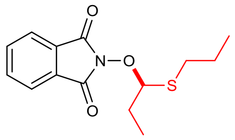

2-(1-(Propylthio)propoxy)isoindoline-1,3-dione (32). Yellow oil; 69% yield; ^{1}H NMR (400 MHz, CDCl$_3$) δ 7.85–7.83 (m, 2H), 7.76–7.74 (m, 2H), 5.30 (dd, J = 7.6, 5.2 Hz, 1H), 2.91–2.84 (m, 1H), 2.76–2.69 (m, 1H), 2.18–2.11 (m, 1H), 1.99–1.90 (m, 1H), 1.66–1.59 (m, 2H), 1.13 (t, J = 7.4 Hz, 3H), 0.99 (t, J = 7.3 Hz, 3H). ^{13}C NMR (101 MHz, CDCl$_3$) δ 164.0, 134.4, 129.0, 123.5, 95.5, 30.8, 26.6, 23.0, 13.6, 10.6. HRMS (ESI-TOF, m/z): $(M+H)^+$ calcd for $C_{14}H_{17}NO_3S$, 280.1002; found, 280.1001.

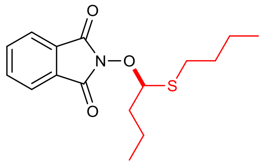

2-(1-(Butylthio)butoxy)isoindoline-1,3-dione (33). Yellow oil; 86% yield; ^{1}H NMR (400 MHz, CDCl$_3$) δ 7.85–7.82 (m, 2H), 7.77–7.73 (m, 2H), 5.37 (dd, J = 7.6, 5.8 Hz, 1H), 2.91 (m, J = 12.1, 8.4, 6.5 Hz, 1H), 2.89–2.85 (m, 1H), 2.76–2.74 (m, 1H), 2.10–2.05 (m, 1H), 1.92–1.88 (m, 1H), 1.57–1.52 (m, 4H), 1.45–1.37 (m, 2H), 0.99 (t, J = 7.4 Hz, 3H), 0.90 (t, J = 7.3 Hz, 3H). ^{13}C NMR (101 MHz, CDCl$_3$) δ 163.0, 133.4, 128.0, 122.5, 92.8, 34.4, 30.6, 28.7, 27.5, 21.1, 18.5, 12.7. ^{1}H NMR and ^{13}C NMR data correspond to the reported values.[1]

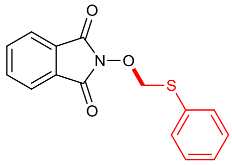

2-((Phenylthio)methoxy)isoindoline-1,3-dione (34). White solid; 76% yield; m.p. 118–120 °C; ^{1}H NMR (400 MHz, CDCl$_3$) δ 7.83–7.81 (m, 2H), 7.76–7.74 (m, 2H), 7.69–7.66 (m, 2H), 7.31–7.29(m, 2H), 7.25–7.22 (s, 1H), 5.60 (s, 2H). ^{13}C NMR (101 MHz, CDCl$_3$) δ 163.5, 134.5, 131.0, 129.1, 128.8, 127.7, 123.6, 82.7, 77.2. HRMS (ESI-TOF, m/z): $(M+Na)^+$ calcd for $C_{15}H_{11}NO_3S$, 308.0352; found, 308.0348.

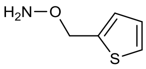

O-(thiophen-2-ylmethyl)hydroxylamine (35). Brown solid; 91% yield; ^{1}H NMR (400 MHz, $CDCl_3$) δ 7.31 (dd, J = 5.0, 1.2 Hz, 1H), 7.06 (dd, J = 3.5, 1.2 Hz, 1H), 7.01–6.99 (m, 1H), 5.47 (s, 2H), 4.84 (s, 2H). ^{13}C NMR (101 MHz, $CDCl_3$) δ 139.9, 127.4, 126.8, 126.3, 72.0.

Supplementary References

1 Xu, X. *et al.* Copper nitrate-catalyzed oxidative coupling of unactivated C(sp3)–H bonds of ethers and alkanes with N-hydroxyphthalimide: synthesis of N-hydroxyimide esters. *Organic & Biomolecular Chemistry* **15**, 9875-9879, doi:10.1039/C7OB02249B (2017).

2 Calder, A., Forrester, A. R. & Thomson, R. H. Nitroxide radicals. Part IV. The oxidation of cyclic N-hydroxy-imides. *Journal of the Chemical Society C: Organic*, 512-516, doi:10.1039/J39690000512 (1969).

3 Zhang, J., Li, Y., Xu, R. & Chen, Y. Donor–Acceptor Complex Enables Alkoxyl Radical Generation for Metal-Free C(sp3)–C(sp3) Cleavage and Allylation/Alkenylation. *Angewandte Chemie International Edition* **56**, 12619-12623, doi:10.1002/anie.201707171 (2017).

4 Chen, X. *et al.* Catalyst-Free Decarboxylation of Carboxylic Acids and Deoxygenation of Alcohols by Electro-Induced Radical Formation. *Chemistry – A European Journal* **26**, 3226-3230, doi:10.1002/chem.201905224 (2020).

5 Krylov, I. B., Lopat'eva, E. R., Budnikov, A. S., Nikishin, G. I. & Terent'ev, A. O. Metal-Free Cross-Dehydrogenative C–O Coupling of Carbonyl Compounds with N-Hydroxyimides: Unexpected Selective Behavior of Highly Reactive Free Radicals at an Elevated Temperature. *The Journal of Organic Chemistry* **85**, 1935-1947, doi:10.1021/acs.joc.9b02656 (2020).

6 Wang, M.-Z. *et al.* Design, synthesis and antifungal activities of novel pyrrole alkaloid analogs. *European Journal of Medicinal Chemistry* **46**, 1463-1472, doi:https://doi.org/10.1016/j.ejmech.2011.01.031 (2011).

7 Jiang, H. *et al.* Ultrasound accelerated synthesis of O-alkylated hydroximides under solvent- and metal-free conditions. *Organic & Biomolecular Chemistry* **17**, 10223-10227, doi:10.1039/C9OB02245G (2019).

8 Guo, Z., Jin, C., Zhou, J. & Su, W. Copper(ii)-catalyzed cross dehydrogenative coupling reaction of N-hydroxyphthalimide with alkanes and ethers via unactivated C(sp3)–H activation at room temperature. *RSC Advances* **6**, 79016-79019, doi:10.1039/C6RA14697J (2016).

9 Michigami, K., Murakami, H., Nakamura, T., Hayama, N. & Takemoto, Y. Catalytic asymmetric aza-Michael addition of fumaric monoacids with multifunctional thiourea/boronic acids. *Organic & Biomolecular Chemistry* **17**, 2331-2335, doi:10.1039/C9OB00045C (2019).
