# Supplementary material for: Iodine(III) promotes cross-dehydrogenative coupling of N-hydroxyphthalimide and unactivated C(sp3)–H bonds
Source: Commun Chem. 2021 Mar 31;4:46. doi: 10.1038/s42004-021-00480-8 (PMC9814821; doi:10.1038/s42004-021-00480-8)
Supplement: Supplementary file 2 — Description of Additional Supplementary Files [file 42004_2021_480_MOESM2_ESM.pdf]

Description of Additional Supplementary Files

File Name: Supplementary Data 1
Description: crystallographic cif data of 4, 7and 11

File Name: Supplementary Data 2
Description: The calculated results and XYZ co-ordinates for all optimized structures.

File Name: Supplementary Data 3
Description: The crystallographic information of compound 4.

File Name: Supplementary Data 4
Description: The crystallographic information of compound 11.

File Name: Supplementary Data 5
Description: The crystallographic information of compound 17.
