# Supplementary material for: Iodine(III) promotes cross-dehydrogenative coupling of N-hydroxyphthalimide and unactivated C(sp3)–H bonds
Source: Commun Chem. 2021 Mar 31;4:46. doi: 10.1038/s42004-021-00480-8 (PMC9814821; doi:10.1038/s42004-021-00480-8)
Supplement: Supplementary file 3 — Supplementary Data 1 [file 42004_2021_480_MOESM3_ESM.pdf]

Crystal Data and Structural Refinement of Compounds 4, 11, and 17

Sample preparation: The product **4** was separated by silica gel column chromatography with the eluent of petroleum ether/ethyl acetate = 20:1, then solution was slowly volatilized into crystal **4**.

Experimental

Single crystals of $C_9H_5O_3Cl_2N$[**4**]were[]. A suitable crystal was selected and[]on aXtaLAB Synergy, Single source at offset/far, HyPixdiffractometer. The crystal was kept at293(2) K during data collection. Using Olex2 [1], the structure was solved with the ShelXT [2] structure solution program using Intrinsic Phasing and refined with the olex2.refine [3] refinement package using Gauss-Newton minimisation.

1. Dolomanov, O.V., Bourhis, L.J., Gildea, R.J, Howard, J.A.K. & Puschmann, H. (2009), J. Appl. Cryst. 42, 339-341.
2. Sheldrick, G.M. (2015). Acta Cryst. A71, 3-8.
3. Bourhis, L.J., Dolomanov, O.V., Gildea, R.J., Howard, J.A.K., Puschmann, H. (2015). Acta Cryst. A71, 59-75.

Crystal structure determination of [**4**]

Crystal Data for $C_9H_5O_3Cl_2N$ (M=246.050 g/mol): monoclinic, space group $P2_1/c$ (no. 14),a= 8.2779(5)Å,b= 15.0012(9)Å,c= 8.0661(4)Å,β= 90.474(5),V= 1001.6(1)$Å^3$,Z= 4,T=293(2)K, μ(Mo Kα)= 0.631 mm^{-1},$Dcalc$= 1.632g/cm^3, 3441 reflections measured ($4.92 \leq 2\Theta \leq 57.52$), 2100 unique ($R_{int}$= 0.0202, R_{sigma}= 0.0409) which were used in all calculations. The final$R_1$was 0.0459 (I>=2u(I)) and$wR_2$was 0.1523 (all data).

Refinement model description

Number of restraints - 0, number of constraints - 10.

Details:

1. Fixed Uiso

At 1.2 times of:

All C(H) groups

2.a Ternary CH refined with riding coordinates:

C00D(H00D)

2.b Aromatic/amide H refined with riding coordinates:

C00C(H00C), C00E(H00E), C00F(H00F), C00B(H00B)

This report has been created with Olex2, compiled on 2019.09.11 svn.r3662 for OlexSys. Pleaselet us knowif there are any errors or if you would like to have additional features.

Supplementary Table 1 Crystal data and structure refinement for 4.

Identification code	**4**
Empirical formula	$C_9H_5NO_3Cl_2$
Formula weight	246.050
Temperature/K	293(2)
Crystal system	monoclinic
Space group	$P2_1/c$
a/Å	8.2779(5)
b/Å	15.0012(9)
c/Å	8.0661(4)
α/°	90
β/°	90.474(5)
γ/°	90
Volume/Å^3	1001.6(1)
Z	4
ρ_{calc}g/cm^3	1.632
μ/mm^{-1}	0.631
F(000)	497.4
Radiation	MoKα (λ = 0.71073)
2Θ range for data collection/°	4.922 to 57.524
Index ranges	$-5 \leq h \leq 10$, $-9 \leq k \leq 18$, $-10 \leq l \leq 6$
Reflections collected	3441
Independent reflections	2100 [R_{int}= 0.0202, R_{sigma}= 0.0409]
Data/restraints/parameters	2100/0/136
Goodness-of-fit on F^2	0.972
Final R indexes [I>=2σ (I)]	R_1= 0.0459, wR_2 = 0.1338
Final R indexes [all data]	R_1= 0.0640, wR_2 = 0.1523
Largest diff. peak/hole / e Å^{-3}	0.35/-0.50

Sample preparation: The product **11** was separated by silica gel column chromatography with the eluent of petroleum ether/ethyl acetate = 20:1, then solution was slowly volatilized into crystal **11**.

Experimental

Single crystals of $C_{17}H_{23}NO_3$ [**11**] were []. A suitable crystal was selected and[]on aXtaLAB Synergy, Single source at offset/far, HyPixdiffractometer. The crystal was kept at293(2)K during data collection. Using Olex2 [1], the structure was solved with the ShelXT [2] structure solution program using Intrinsic Phasing and refined with the olex2.refine [3] refinement package using Gauss-Newton minimisation.

1. Dolomanov, O.V., Bourhis, L.J., Gildea, R.J, Howard, J.A.K. & Puschmann, H. (2009), J. Appl. Cryst. 42, 339-341.
2. Sheldrick, G.M. (2015). Acta Cryst. A71, 3-8.
3. Bourhis, L.J., Dolomanov, O.V., Gildea, R.J., Howard, J.A.K., Puschmann, H. (2015). Acta Cryst. A71, 59-75.

Crystal structure determination of [**11**]

Crystal Datafor $C_{17}H_{23}NO_3$(M=289.377 g/mol): monoclinic, space group C2/c (no. 15),a= 24.868(1)Å,b= 6.5294(2)Å,c= 20.2949(9)Å,β= 95.254(4),V= 3281.5(2)Å^3,Z= 14,T=293(2)K, μ(Mo Kα)= 0.139 mm^{-1},$Dcalc$= 2.050 g/cm^3, 10099 reflections measured (4.04 ≤ 2Θ ≤ 58.06), 3730 unique (R_{int}= 0.0217, R_{sigma}= 0.0236) which were used in all calculations. The final$R_1$was 0.0453 (I>=2u(I)) and$wR_2$was 0.1386 (all data).

Refinement model description

Number of restraints - 0, number of constraints - 34.

Details:

Fixed Uiso

At 1.2 times of:

All C(H) groups

At 1.5 times of:

All C(H,H,H) groups

2.a Ternary CH refined with riding coordinates:

C005(H005)

2.b Aromatic/amide H refined with riding coordinates:

C00C(H00C), C00E(H00E), C00G(H00G), C00D(H00D)

2.c Idealised Me refined as rotating group:

C00F(H00a,H00b,H00f), C00H(H00h,H00i,H00j), C00I(H00k,H00l,H00m), C00J(H00n,H00o,H00p), C00K(H00q,H00r,H00s), C00L(H00t,H00u,H00v)

This report has been created with Olex2, compiled on 2019.09.11 svn.r3662 for OlexSys. Pleaselet us knowif there are any errors or if you would like to have additional features.

Supplementary Table 2 Crystal data and structure refinement for 11

Identification code	**11**
Empirical formula	$C_{17}H_{23}NO_3$
Formula weight	289.377
Temperature/K	293(2)
Crystal system	monoclinic
Space group	C2/c
a/Å	24.868(1)
b/Å	6.5294(2)
c/Å	20.2949(9)
α/°	90
β/°	95.254(4)
γ/°	90
Volume/Å^3	3281.5(2)
Z	14
ρ_{calc}g/cm^3	2.050

μ/mm^{-1}	0.139
F(000)	2185.1
Crystal size/mm^3	0.02 × 0.01 × 0.01
Radiation	Mo Kα (λ = 0.71073)
2Θ range for data collection/°	4.04 to 58.06
Index ranges	$-25 \leq h \leq 31$, $-7 \leq k \leq 8$, $-25 \leq l \leq 21$
Reflections collected	10099
Independent reflections	3730 [R_{int}= 0.0217, R_{sigma}= 0.0236]
Data/restraints/parameters	3730/0/196
Goodness-of-fit on F^2	1.028
Final R indexes [I>=2σ (I)]	R_1= 0.0453, wR_2= 0.1268
Final R indexes [all data]	R_1= 0.0453, wR_2= 0.1268
Largest diff. peak/hole / e $Å^{-3}$	0.20/-0.24

Sample preparation: The product **17** was separated by silica gel column chromatography with the eluent of petroleum ether/ethyl acetate = 20:1, then solution was slowly volatilized into crystal **17**.

Experimental

Single crystals of $C_{13}H_9NO_3$ [**17**] were []. A suitable crystal was selected and[]on aBruker Smart Apex CCDdiffractometer. The crystal was kept at 293(2) K during data collection. Using Olex2 [1], the structure was solved with the ShelXT [2] structure solution program using Intrinsic Phasing and refined with the olex2.refine [3] refinement package using Gauss-Newton minimisation.

1. Dolomanov, O.V., Bourhis, L.J., Gildea, R.J, Howard, J.A.K. & Puschmann, H. (2009), J. Appl. Cryst. 42, 339-341.
2. Sheldrick, G.M. (2015). Acta Cryst. A71, 3-8.

3. Bourhis, L.J., Dolomanov, O.V., Gildea, R.J., Howard, J.A.K., Puschmann, H. (2015). Acta Cryst. A71, 59-75.

Crystal structure determination of[**17**]

Crystal Datafor $C_{13}H_9NO_3S$ (M=259.287 g/mol): monoclinic, space group P2$_1$(no. 4),a= 6.6945(4)Å,b= 4.8532(2)Å,c= 18.8568(11)Å,β= 96.539(5),V= 608.67(6)Å^3,Z= 2,T= 293(2) K, μ(Mo Kα)= 0.264 mm^{-1},$Dcalc$= 1.415g/cm^3, 3674 reflections measured (4.34 ≤ 2Θ ≤ 57.66), 2593 unique (R_{int}= 0.0213, R_{sigma}= 0.0503) which were used in all calculations. The final$R_1$was 0.0450 (I>=2u(I)) and$wR_2$was 0.1085 (all data).

Refinement model description

Number of restraints - 1, number of constraints - 17.

Details:

1. Fixed Uiso

At 1.2 times of:

All C(H) groups, All C(H,H) groups

2.a Secondary CH2 refined with riding coordinates:

C00I(H00a,H00i)

2.b Aromatic/amide H refined with riding coordinates:

C00F(H00F), C00E(H00E), C00C(H00C), C00B(H00B), C00D(H00D), C00G(H00G),

C00H(H00H)

This report has been created with Olex2, compiled on 2019.09.11 svn.r3662 for OlexSys. Pleaselet us know if there are any errors or if you would like to have additional features.

Supplementary Table 3 Crystal data and structure refinement for 17

Identification code	**17**
Empirical formula	$C_{13}H_9NO_3S$
Formula weight	259.287

Temperature/K	293(2)
Crystal system	monoclinic
Space group	$P2_1$
a/Å	6.6945(4)
b/Å	4.8532(2)
c/Å	18.8568(11)
α/°	90
β/°	96.539(5)
γ/°	90
Volume/Å^3	608.67(6)
Z	2
ρ_{calc}g/cm^3	1.415
μ/mm^{-1}	0.264
F(000)	268.4
Radiation	MoKα (λ = 0.71073)
2Θ range for data collection/°	4.348 to 57.662
Index ranges	$-6 \le h \le 8, -6 \le k \le 6, -23 \le l \le 22$
Reflections collected	3674
Independent reflections	2593 [R_{int}= 0.0213, R_{sigma}= 0.0503]
Data/restraints/parameters	2593/1/163
Goodness-of-fit on F^2	1.033
Final R indexes [I>=2σ (I)]	R_1= 0.0450, wR_2= 0.0941
Final R indexes [all data]	R_1= 0.0648, wR_2= 0.1085
Largest diff. peak/hole / e Å^{-3}	0.23/-0.29
