# Supplementary material for: Iodine(III) promotes cross-dehydrogenative coupling of N-hydroxyphthalimide and unactivated C(sp3)–H bonds
Source: Commun Chem. 2021 Mar 31;4:46. doi: 10.1038/s42004-021-00480-8 (PMC9814821; doi:10.1038/s42004-021-00480-8)
Supplement: Supplementary file 4 — Supplementary Data 2 [file 42004_2021_480_MOESM4_ESM.pdf]

The calculated results and XYZ co-ordinates for all optimized structures

Supplementary Table 4. The calculated results

	Sum of electronic and zero-point Energies= (Hatree)	Sum of electronic and thermal Enthalpies= (Hatree)	Sum of electronic and thermal Free Energies= (Hatree)	BDE (kJ/mol)	ΔH (kJ/mol)	ΔrG (kJ/mol)
NHPI	-587.945664	-587.935491	-587.980161	-	-	-
PINO radical	-587.331633	-587.322099	-587.366246	-	-	-
Cyclohexane	-235.650608	-235.644013	-235.677687	-	-	-
Cyclohexane radical	-234.99982	-234.992855	-235.029591	-	-	-
DCM	-959.618621	-959.614026	-959.644938	-	-	-
DCM radical	-958.962118	-958.957469	-958.989551	-	-	-
H radical	-0.500273	-0.497912	-0.510927	-	-	-
$PhI(OAc)_2$	-699.488887	-699.471004	-699.538385	-	-	-
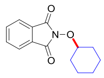	-822.407933	-822.391989	-822.451542	-	-	-
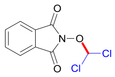	-1546.372479	-1546.358995	-1546.413432	-	-	-
PhI	-242.883837	-242.87709	-242.915565	-	-	-
AcOH	-228.943942	-228.938451	-228.971058	-	-	-
Cyclohexane-H—> Cyclohexane radical + H				402.3	-	-
DCM—> DCM radical+ H				416.5	-	-
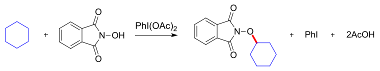				-	-250.7	-296.6
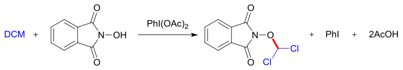				-	-242.8	-282.6

Data presented here correspond to:

Atomic number, x_i, y_i, z_i

(NHPI)

C	1.689545	1.425491	-0.000960
C	0.503597	0.703402	0.004604
C	0.503401	-0.703251	0.004835
C	1.689242	-1.425630	-0.000651
C	2.894160	-0.701793	-0.012868
C	2.894301	0.701320	-0.012987
H	1.681102	2.509654	0.000358

H	1.680548	-2.509798	0.000568
H	3.838624	-1.235316	-0.023023
H	3.838894	1.234612	-0.023144
C	-0.904077	-1.187949	0.020220
C	-0.903844	1.188124	0.020045
O	-1.347477	2.338114	-0.001249
O	-1.347916	-2.337761	-0.001568
O	-3.055583	-0.000051	-0.177925
H	-3.511318	-0.000146	0.694459
N	-1.673999	0.000043	0.094756

(PINO radical)

C	-1.642142	1.425548	-0.000177
C	-0.452993	0.704546	-0.000288
C	-0.453004	-0.704568	-0.000020
C	-1.642173	-1.425510	0.000183
C	-2.845471	-0.702327	0.000230
C	-2.845475	0.702413	0.000077
H	-1.633617	2.509573	-0.000328
H	-1.633741	-2.509536	0.000183
H	-3.790060	-1.235318	0.000394
H	-3.790073	1.235382	0.000155
C	0.938872	-1.201964	-0.000180
C	0.938838	1.202036	-0.000034
O	1.381331	2.347111	-0.000102
O	1.381117	-2.347237	-0.000388
O	3.058641	-0.000100	0.000439
N	1.757151	0.000095	0.000178

(Cyclohexane)

C	1.275087	0.736172	0.231032
H	1.332324	-0.769218	-1.330010
H	1.332324	0.769218	1.330010
H	2.168066	1.251733	-0.146260
C	0.000000	1.472343	-0.231032
H	0.000000	2.503467	0.146260
H	0.000000	1.538436	-1.330010
C	-1.275087	0.736172	0.231032
H	-1.332324	0.769218	1.330010
H	-2.168066	1.251733	-0.146260
C	-1.275087	-0.736172	-0.231032
H	-2.168066	-1.251733	0.146260
H	-1.332324	-0.769218	-1.330010
C	-0.000000	-1.472343	0.231032

H	-0.000000	-2.503467	-0.146260
H	2.168066	-1.251733	0.146260
H	0.000000	-1.538436	1.330010
C	1.275087	-0.736172	-0.231032

(Cyclohexane radical)

C	-0.049197	-0.796716	1.294743
H	-1.087854	1.112979	1.304587
H	0.990420	-1.131485	1.489417
H	-0.645373	-1.165426	2.139234
C	-0.541232	-1.375215	-0.000000
H	-1.084313	-2.317028	-0.000000
C	-0.049197	-0.796716	-1.294743
H	0.990420	-1.131485	-1.489417
H	-0.645373	-1.165426	-2.139234
C	-0.049197	0.753899	-1.272576
H	0.451872	1.139953	-2.169843
H	-1.087854	1.112979	-1.304587
C	0.631760	1.295961	0.000000
H	0.611025	2.393854	0.000000
H	0.451872	1.139953	2.169843
H	1.692710	1.000456	-0.000000
C	-0.049197	0.753899	1.272576

(DCM)

C	-0.000000	0.000000	0.804765
H	-0.907756	0.000000	1.397554
H	0.907756	-0.000000	1.397554
Cl	-0.000000	1.543100	-0.224226
Cl	-0.000000	-1.543100	-0.224226

(DCM radical)

C	0.013325	0.735059	-0.000000
H	-0.533020	1.668377	-0.000000
Cl	0.013325	-0.178786	1.535188
Cl	0.013325	-0.178786	-1.535188

(PhI(OAc)$_2$)

C	0.984695	-3.333566	0.710456
C	0.998103	-1.929902	0.719527
C	0.000186	-1.285807	-0.000007
C	-0.997553	-1.930186	-0.719534
C	-0.983736	-3.333845	-0.710456
C	0.000582	-4.030400	-0.000003
H	1.747783	-3.869923	1.264132

H	1.756412	-1.370499	1.253536
H	-1.756036	-1.370983	-1.253507
H	-1.746675	-3.870422	-1.264121
H	0.000737	-5.115221	-0.000007
I	-0.000121	0.920128	0.000025
O	2.055465	0.778865	0.706861
C	3.220877	0.938927	-0.001846
O	4.287456	0.611186	0.526153
O	-2.055702	0.778272	-0.706829
C	-3.221163	0.938351	0.001780
O	-4.287700	0.610636	-0.526322
C	-3.133441	1.528039	1.394004
H	-2.559086	0.877388	2.063419
H	-2.641741	2.507185	1.378072
H	-4.141294	1.642400	1.793307
C	3.133020	1.528771	-1.393990
H	2.558621	0.878192	-2.063435
H	2.641293	2.507895	-1.377865
H	4.140831	1.643237	-1.793349

(PhI)

C	-2.670326	-1.210447	0.000000
C	-1.268986	-1.217902	0.000003
C	-0.583641	-0.000029	-0.000009
C	-1.268966	1.217887	-0.000004
C	-2.670277	1.210476	0.000006
C	-3.372225	0.000013	-0.000003
H	-3.207051	-2.153877	0.000004
H	-0.728230	-2.156631	0.000003
H	-0.728145	2.156579	-0.000004
H	-3.207016	2.153899	0.000011
H	-4.457212	0.000051	-0.000005
H	1.572343	-0.000000	0.000000

(AcOH)

C	1.397411	-0.132856	0.000019
H	1.674025	-0.719187	-0.882210
H	1.673594	-0.721031	0.881056
H	1.938063	0.813218	0.000978
C	-0.078072	0.131749	0.000014
O	-0.629071	1.234473	0.000002
O	-0.800684	-1.048470	0.000027
H	-1.763680	-0.854376	-0.000252
